# Discovery and Structure-Based Design of Potent Covalent PPARγ Inverse-Agonists **BAY-4931** and **BAY-0069**

**DOI:** 10.1021/acs.jmedchem.2c01379

**Published:** 2022-10-21

**Authors:** Douglas L. Orsi, Elisabeth Pook, Nico Bräuer, Anders Friberg, Philip Lienau, Christopher T. Lemke, Timo Stellfeld, Ulf Brüggemeier, Vera Pütter, Hanna Meyer, Maria Baco, Stephanie Tang, Andrew D. Cherniack, Lindsay Westlake, Samantha A. Bender, Mustafa Kocak, Craig A. Strathdee, Matthew Meyerson, Knut Eis, Jonathan T. Goldstein

**Affiliations:** † Center for the Development of Therapeutics, Broad Institute of MIT and Harvard, Cambridge, Massachusetts 02142, United States; ‡ Research and Development, Pharmaceuticals, Bayer AG, 13353 Berlin, Germany; § Nuvisan ICB GmbH, 13353 Berlin, Germany; ∥ Cancer Program, Broad Institute of MIT and Harvard, Cambridge, Massachusetts 02142, United States; ⊥ Department of Medical Oncology, Dana-Farber Cancer Institute, Boston, Massachusetts 02215, United States; # Center for Cancer Genomics, Dana-Farber Cancer Institute, Boston, Massachusetts 02215, United States; ∇ Department of Genetics and Medicine, Harvard Medical School, Boston, Massachusetts 02115, United States

## Abstract

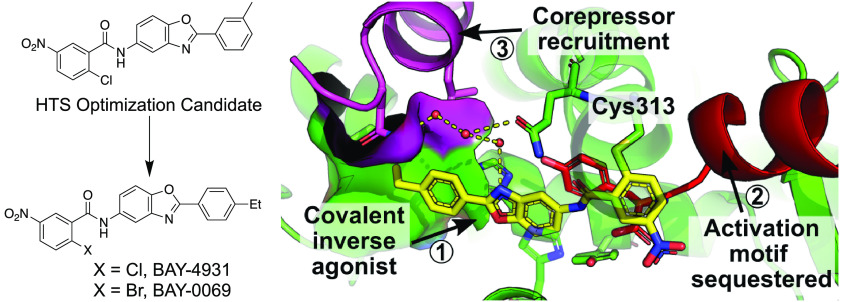

The ligand-activated nuclear receptor peroxisome-proliferator-activated receptor-γ (PPARG or PPARγ) represents a potential target for a new generation of cancer therapeutics, especially in muscle-invasive luminal bladder cancer where PPARγ is a critical lineage driver. Here we disclose the discovery of a series of chloro-nitro-arene covalent inverse-agonists of PPARγ that exploit a benzoxazole core to improve interactions with corepressors NCOR1 and NCOR2. *In vitro* treatment of sensitive cell lines with these compounds results in the robust regulation of PPARγ target genes and antiproliferative effects. Despite their imperfect physicochemical properties, the compounds showed modest pharmacodynamic target regulation *in vivo*. Improvements to the *in vitro* potency and efficacy of **BAY-4931** and **BAY-0069** compared to those of previously described PPARγ inverse-agonists show that these compounds are novel tools for probing the *in vitro* biology of PPARγ inverse-agonism.

## Introduction

**Figure 1:**
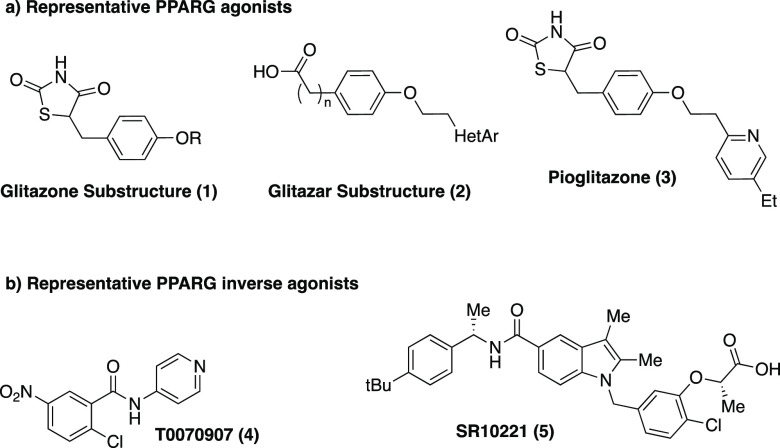
Representative PPARγ modulators. (A) Representative PPARγ agonists with clinical utility. (B) Representative PPARγ inverse-agonists.

Peroxisome-proliferator-activated receptor gamma (PPARG or PPARγ) is a ligand-activated nuclear receptor and master regulator of adipogenesis.^1^ PPARγ is the target of the glitazone (**1**) and glitazar (**2**) (Figure 1A) families of drugs used clinically for the treatment of lipid and glucose dysregulation associated with type 2 diabetes.^2^ Activation of PPARγ through somatic alterations in the *PPARG* gene and its partner protein *RXRA* are oncogenic,^3−5^ and high *PPARG* gene expression is the top predictive biomarker of *PPARG* genetic dependency in large-scale genome-wide genetic perturbation studies in the Cancer Dependency Map (DepMap.org);^6^ the cell lines with *PPARG* focal gene amplification (UM-UC-9 and 5637) and the *RXRA* p.S427F hotspot mutation (HT1197) are outliers of particular interest. Additionally, pharmacological PPARγ agonists, including pioglitazone (**3**), are associated with an increased risk of bladder cancer.^7−9^ Pharmacological antagonism of PPARγ *in vitro* has been shown to lead to antiproliferative effects in PPARγ-activated cancer cell lines^3^ and to promote osteogenesis,^10^ and genetic experiments suggest that PPARγ inverse agonists may induce proinflammatory effects in the tumor environment.^4^

While most described PPARγ modulators are agonists, there are a small handful of described inverse-agonists, including T0070907 (**4**) and SR10221 (**5**)^10,11^ (Figure 1B), though none have progressed to clinical evaluation. To test the therapeutic hypothesis that inducing a repressive PPARγ complex using an inverse-agonist might be clinically beneficial for patients, we set out to identify potent and selective inverse-agonists with favorable pharmacokinetic and pharmacodynamic properties. Here we report the discovery of new potent and efficacious PPARγ covalent inverse-agonists. While these compounds are limited in terms of their *in vivo* pharmacokinetic properties, they are valuable tools for *in vitro* studies.

## Results and Discussion

**Figure 2:**
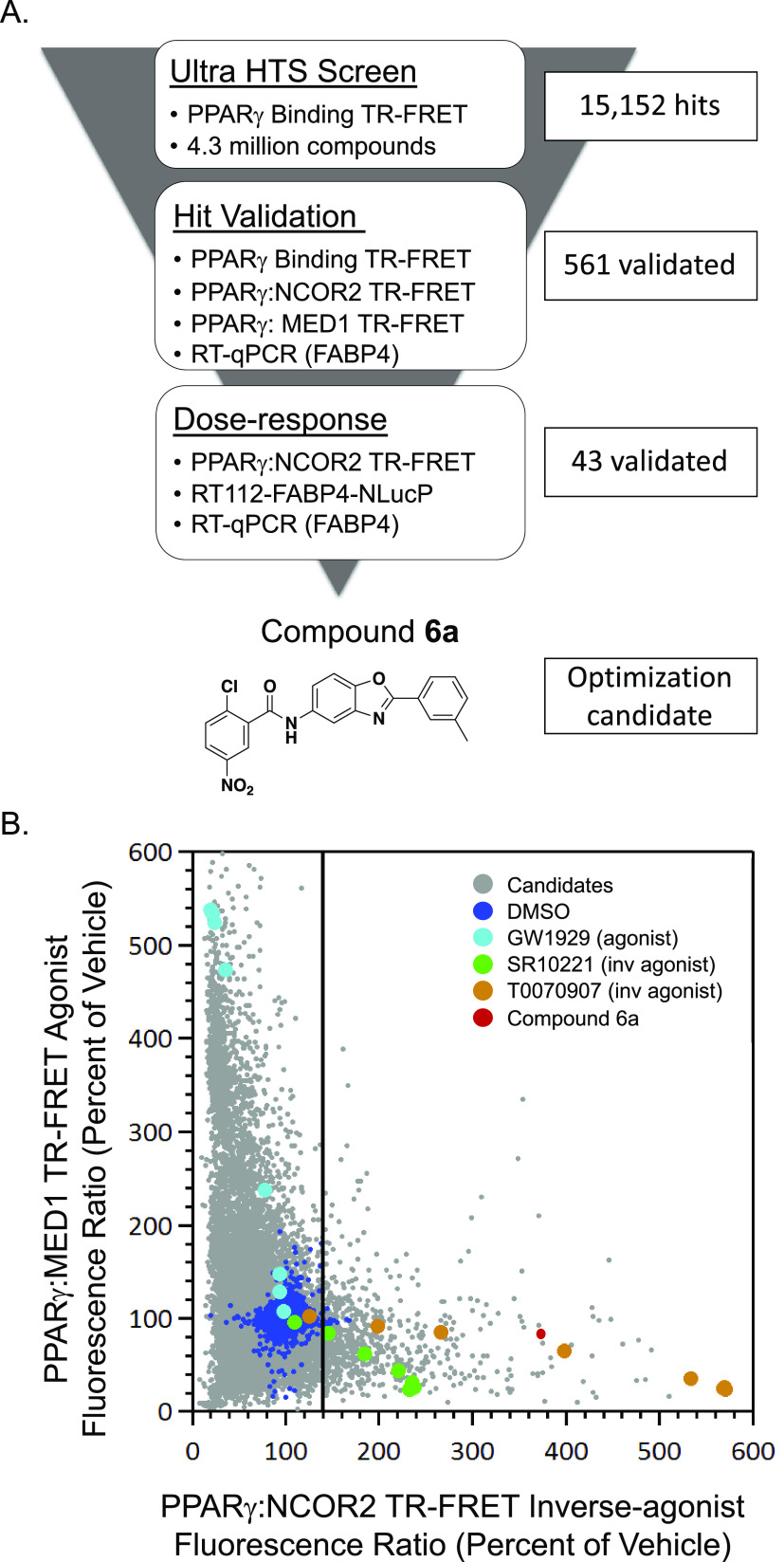
Discovery of PPARγ inverse-agonists. (A) Representation of the hit-finding screening cascade from an ultrahigh-throughput competitive ligand-binding assay of 4.3 million compounds, single-concentration hit validation, and dose–response curves used to identify candidate hit **6a**. (B) Hit validation comparing the fluorescent signal from PPARg:MED1 coactivator recruitment and PPARg:NCOR2 corepressor recruitment (CRR) LanthaScreen TR-FRET interaction assays for candidate compounds tested at 10 μM. PPARg probe compounds were added in dose titration to benchmark assay performance. Probe compounds included GW1929 (agonist), SR10221 (inverse-agonist), and T0070907 (inverse-agonist).

A high-throughput screening campaign for PPARγ inverse-agonists was undertaken as outlined in Figure 2A. A chemical library containing more than 4 million compounds was interrogated using an ultrahigh-throughput PPARγ biochemical competitive binding assay to identify binders of the PPARγ ligand binding domain (LBD) that were competitive the fluorescent-labeled PPARγ ligand, *i.e.*, fluormone. Compounds that bind to the binding pocket of the PPARγ LBD lead to a decrease in TR-FRET signal between fluormone and labeled PPARγ. Compounds were screened in duplicate at a single concentration (10 μM). Candidate binders identified in the primary screen were validated by retesting them in the PPARγ binding assay, and there were a total of 15 152 primary hits and 11 421 confirmed hits for an overall positive rate of 0.3% (Chart S1) for PPARγ binders.

To deconvolute the functional activity and prioritize confirmed binders, functional biochemical and cellular assays were performed at a single dose (10 μM) in a panel of assays. A PPARγ:MED1 TR-FRET biochemical coactivator interaction assay was used to measure the effects of compounds on the interaction between the PPARγ LBD and a peptide containing the “LxxLL” nuclear receptor interaction motif from the coactivator, *i.e.*, MED1 (TRAP220/DRIP-205). A signal increase is indicative of a potential agonist, and known PPARγ agonist GW1929^12^ responded accordingly. To investigate candidate inverse-agonists, the assay was adapted to use a peptide containing the interaction motif from the corepressor, *i.e.*, NCOR2 (Smrt ID2)^13^ in place of the MED1 peptide. Compound-induced interactions between the PPARγ LBD and the peptide from the corepressor NCOR2 would be indicative of inverse-agonists. Known inverse-agonists T0070907^11^ and SR10221^10^ responded accordingly.

A large proportion of the initially confirmed high-throughput screening (HTS) candidates did not show activity in the coregulator recruitment assays (bottom-left quadrant of Figure 2B) and were likely simple binders or neutral antagonists. Another large fraction overlapped with the PPARγ agonist GW1929 in terms of the directionality of the assay signal (upper-left quadrant of Figure 2B). PPARγ inverse-agonists T0070907 and SR10221 demonstrated a concentration-dependent increase in signal in the NCOR2 biochemical assay. A small number of candidates led to a signal increase in the corepressor recruitment assay (lower-right quadrant of Figure 2B), indicating potential inverse-agonists. Interestingly, agonists led to decreased signals in the inverse-agonist biochemical assay with NCOR2 peptide, and inverse-agonists conversely decreased the signal in the agonist assay with the MED1 peptide. This indicates that inverse-agonists may induce a structural conformation that disrupts basal interactions between PPARγ and coactivators, further shifting the equilibrium from activation to repression. Whether this also occurs in the cellular context is unclear. To eliminate possible assay artifacts and verify activity in a cellular context, high-throughput RT-qPCR monitoring of the mRNA transcripts of the canonical PPARγ target gene *FABP4* was performed. The effects of compounds on the expression of *FABP4* in RT112 cells were also monitored using a high-throughput nanoluciferase reporter assay (RT112-FABP4-NLucP^3^) to evaluate and triage candidate compounds based on cellular activity with a single dose of 10 μM.

Subsequent predictive filtering of chemical structures to eliminate undesirable structures refined the list to 561 preferred candidates for testing in a dose–response format. Preferred hits were evaluated using a corepressor recruitment TR-FRET assay that measured ligand-dependent changes in the interaction between PPARγ and a peptide from NCOR2 in addition to RT-qPCR of the canonical PPARγ target gene *FABP4*. Compounds were also tested in a previously described cellular reporter assay for PPARγ transactivation, *i.e.*, the RT112-FABP4_NLucP reporter assay.^3^

To select the most suitable starting point for the candidate optimization effort, we started with an absolute requirement for confirmed binding in the competitive binding assay, in addition to a dose-dependent increase in signal in the PPARγ:NCOR2 interaction assay. Candidates were further triaged by a requirement for dose-dependent inverse-agonist activity in one or more of the cellular assays in PPARγ-activated bladder cancer cell lines, including RT-qPCR in RT112 cells, or RT112-FABP4-NLucP. Five favored chemotypes were identified. One promising candidate, **6a**, which has the same chloro-nitro-benzamide covalent warhead as T0070907 (compound 4), was selected for optimization.

**Figure 3:**
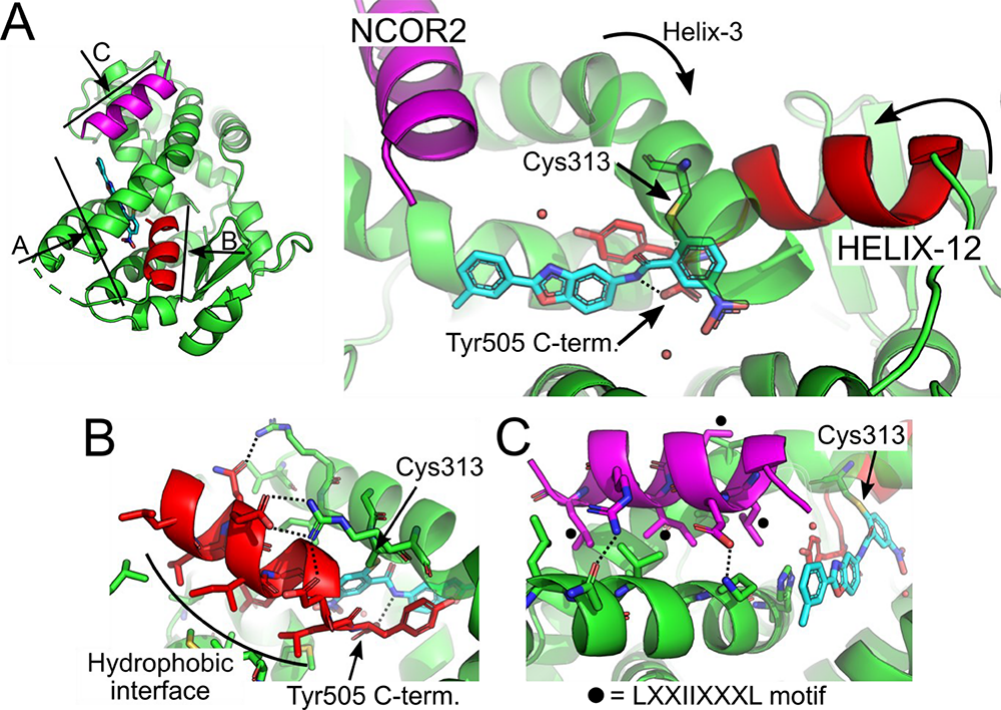
Crystal structure of PPARγ bound to the NCOR2 corepressor peptide and the inverse-agonist compound **6a** (PDB ID 8AQM). (A) Inset showing the complete trimeric complex with the views of the different panels indicated by lines and arrows (left). Overview of the PPARγ (green) cocomplex with the C-terminal Helix-12 (red) bound behind the kinked Helix-3, the NCOR2 peptide (magenta), and compound **6a** (cyan) covalently bound to Cys313 (right). The C-terminus, Tyr505, interacts directly with **6a**. (B) Intramolecular interactions of Helix-12 when sequestered in the canonical ligand binding site. (C) NCOR2 corepressor binding interactions to PPARγ.

To help guide medicinal chemistry efforts, **6a** was cocrystallized with the PPARγ LBD bound to a peptide derived from the corepressor NCOR2 (Figure 3). **6a** covalently modified C313 of PPARγ isoform 2 in a manner similar to T0070907.^14^ Crystal structures of **6a** and T0070907 show that Helix-12, an essential interaction surface for coactivator proteins, is sequestered in the canonical ligand binding site and is thus unavailable to recruit the coactivators (Figure 3B). The conserved residues of the hydrophobic receptor interaction motif (LXXIIXXXL) of NCOR2 are key to the the interaction with PPARγ bound to the inverse-agonists (Figure 3C). Intriguingly, the C-terminal residue (Y475 in PPARγ isoform 1 NP_005028 or Y505 in PPARγ isoform 2 NP_056953.2) interacts directly with the amide linker of the inverse-agonist.

**Scheme 1:**
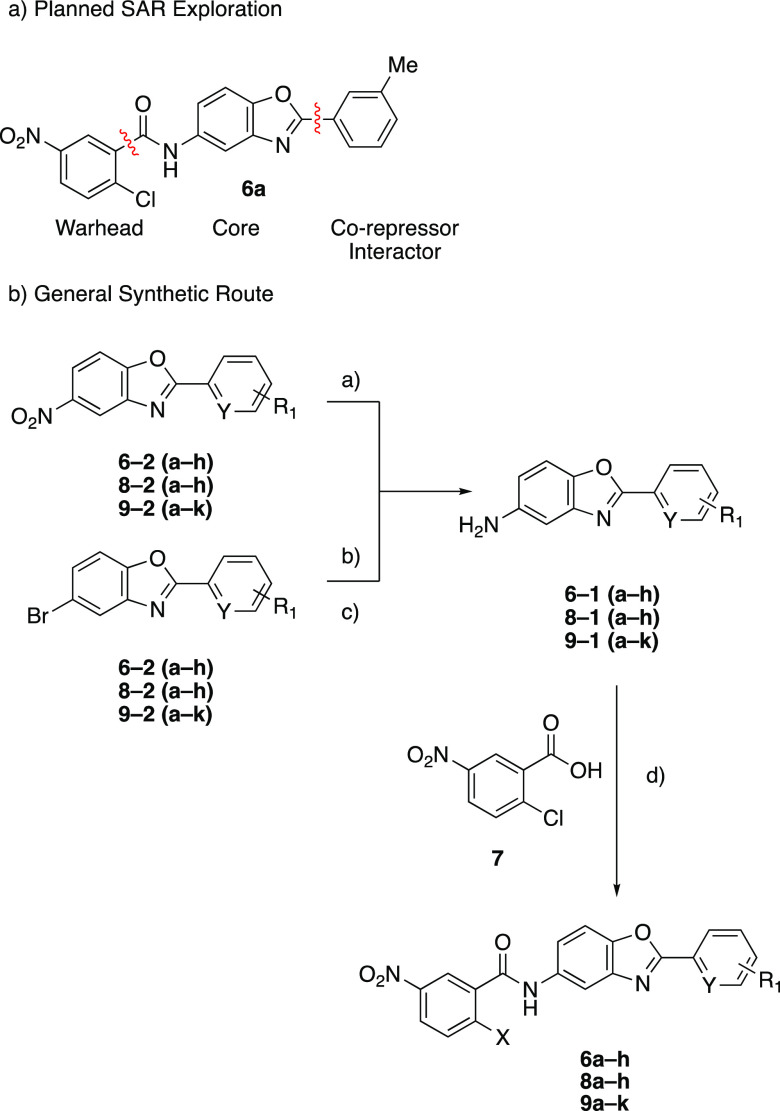
SAR Strategy and General Synthesis of Target Compounds Reaction conditions are as follows: (a) SnCl_2_ (4 equiv), EtOH, reflux. (b) *tert*-butyl carbamate (1.2 equiv), NaO(*tert*-butyl) (2 equiv), *tert*-butyl-XPhos (0.1 equiv), Pd(dba)_2_ (0.03 equiv), PhMe, 60 °C. (c) 4 M HCl in 1,4-dioxane, 25 °C. (d) (i) **7** (1.03 equiv), SOCl_2_ (excess), 80 °C, concentrated; (ii) **6–1** (**a–h**) (1 equiv), Et_3_N (5 equiv), THF, 25 °C.

Compared to known inverse-agonist T0070907, the lipophilic tail of **6a**, consisting of the benzoxazole and a 3-tolyl group, extends further toward the lipophilic corepressor binding surface. The binding region is relatively narrow, with the potential to interact with the solvent around the central benzoxazole ring. To systematically investigate the insights gleaned from this crystal structure, the structure–activity relationship (SAR) for the compound was evaluated within three structural regions (Scheme 1), *i.e.*, the NCOR-interacting ring, the potentially solvent-interacting core, and the covalent warhead.

## SAR

Initial efforts were focused on improving the interactions between compounds and NCOR2. Compounds with variable NCOR-interacting rings were synthesized according to Scheme 1. Synthesis of the necessary anilines was achieved by either reduction of the corresponding nitro-arene under SnCl_2_ conditions or Buchwald–Hartwig coupling of the corresponding aryl-bromide with *tert*-butyl carbamate, followed by Boc deprotection with HCl in 1,4-dioxane. The resulting anilines were then reacted with the aryl chloride of the corresponding covalent warhead to provide the desired products.

A variety of small substituents on the aryl ring at different positions showed strong preferences for *meta*- or *para*-substitution (**6a** and **6c** (**BAY-4931**)), while *ortho*-substitution (**6b**) dramatically reduced both potency and maximal efficacy in the cellular assays (Table 1). Incorporation of electron-poor rings (**6d** and **6e**) significantly improved the activity, though combining bulk at the *para*-position with an electron deficient ring did not provide an additive effect (**6f**). Substituted 3-pyridines maintained activity in the corepressor recruitment assay (**6g-h**) (Table 1).

**Table 1:**
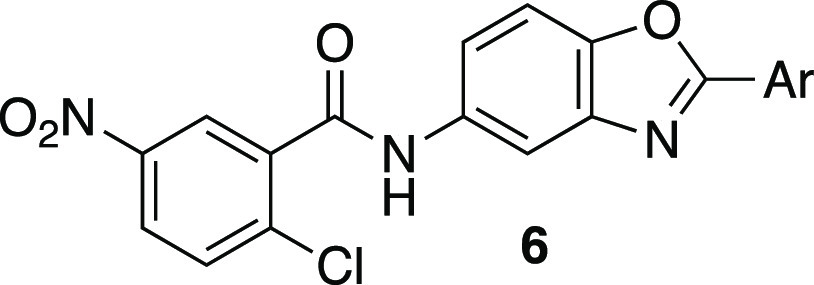

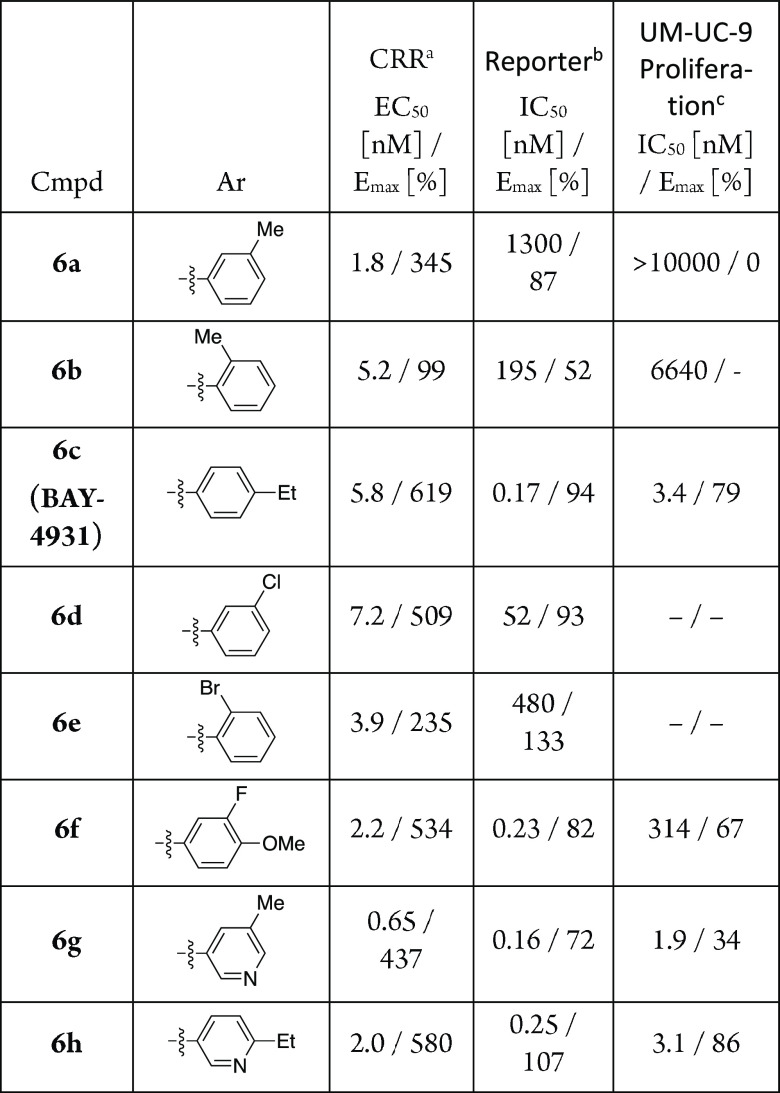
SAR of the Co-Repressor-Interacting Aryl Group

a LanthaScreen TR-FRET PPARγ corepressor recruitment (CRR) assay with PPARγ-LBD and the NCOR2 (Smrt ID2) peptide. Refer to the Experimental Section for assay conditions. EC_50_ and *E*_max_ values represent the values from one or more experiment performed with samples in duplicate.

b RT112-FABP4-NLucP cellular reporter assay for the transactivation of PPARγ. Refer to the Experimental Section for assay conditions. IC_50_ and *E*_max_ values represent the mean from two or more experiments.

c UM-UC-9 proliferation assay. Refer to the Experimental Section for assay conditions. IC_50_ and *E*_max_ values represent mean one or more experiments read out at day 7.

Functional activity was evaluated by testing candidate compounds in the NCOR2 recruitment assay as well as the previously described RT112-FABP4-NLucP cellular reporter assay for PPARγ transactivation.^3^ Again, *para*-substitution was strongly preferred (**BAY-4931**), while the combination of the 3-pyridyl and 4-ethyl substitutions led to favorable effects in cellular assays (**6h**). Notably, the variability in maximal efficacy (*E*_max_) in the cellular assays was less pronounced than that in the biochemical assay. To profile the antiproliferative effect of functional inverse-agonists, compounds were evaluated in UM-UC-9 bladder cancer cells, which are exquisitively sensitive to PPARγ modulation and possess a focal amplification of the *PPARG* gene to greater than approximately 25 copies.^3^ Again, *para*-substitution was strongly preferred, as only **BAY-4931** and **6h** demonstrated compelling antiproliferative potency and efficacy (Table 1). Interestingly, **6g** maintained potency but had poor efficacy in the UM-UC-9 proliferation assay, indicative of a partial inverse-agonist.

**Figure 4:**
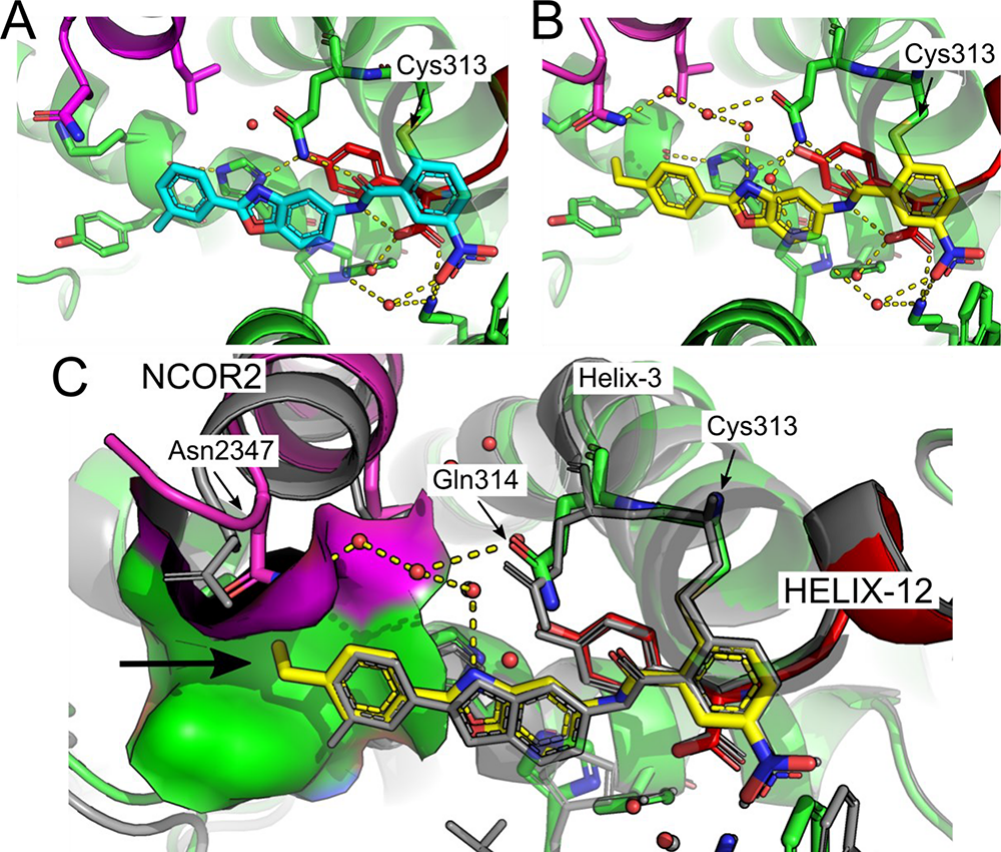
Structural details of the interactions between PPARγ or NCOR2 and **6a** (PDB ID 8AQN) and the optimized inverse-agonist BAY-4931 (PDB ID 8AQN). (A) Co-crystal structure of PPARγ (green), the NCOR2 peptide (magenta), and **6a** (cyan). Polar interactions are highlighted with yellow dashes. (B) Co-crystal structure of PPARγ (green), the NCOR2 peptide (magenta), and BAY-4931 (yellow). (C) Comparison of the crystal structures with **6a** (gray) and BAY-4931 (colored as in panel B). A water-mediated interaction among BAY-4931, Gln314, and Asn2347 of the NCOR2 peptide is highlighted. The additional hydrophobic interaction of the *para*-ethyl group of BAY-4931 is indicated by a black arrow (lower left).

The increased *E*_max_ observed in the corepressor recruitment assay for *para*-substituted compounds may be explained by increased or stabilized interactions with the NCOR2 peptide. To test this hypothesis, **BAY-4931** was cocrystallized with PPARγ (Figure 4). The covalently bound inverse-agonist **6a** extends toward the NCOR2 peptide and is located slightly underneath the N-terminus of the corepressor (Figure 4A). *para*-Substituted **BAY-4931** shows hints of the stabilized recruitment of the NCOR2 peptide (Figure 4B). The NCOR2 peptide exhibited a modest shift in position, and two additional N-terminal residues of the peptide could be modeled with confidence. The introduced ethyl substitution fits well into a niche formed between the receptor and the corepressor. Addtionally, with BAY-4931, a more extensive water-network was observed that extended all the way from PPARγ–BAY-4931 to the NCOR2 peptide. Comparison of the two complexes otherwise shows minimal changes in the ligand binding mode and protein conformations (Figure 4C). Extending the N-terminus of the NCOR2 peptide did not change any of the observed interactions or increase the number of resolved residues. Taken together, this is in line with the biochemical and cellular data of the two compounds (Table 1), showing **BAY-4931** is more potent and efficacious than **6a**. The hypothesis that a stabilized binding mode of the inverse-agonist is important for increased efficacy fits with the finding that T0070907 exhibits conformational dynamics and a different binding mode when bound to PPARγ prior to recruitment of NCOR2, as described by Shang et al.^14^ (PDB ID 6C1I). The observed flipped binding mode of T0070907 does not allow for any interactions to NCOR2, and Helix-12 is also not sequestered (Figure S4).

Selected active compounds from Table 1 were profiled for metabolic stability, Caco-2 permeability, aqueous solubility, glutathione (GSH) stability, and extent of the covalent modification of the PPARγ ligand binding domain (Table 2). All compounds tested were poorly soluble and highly metabolized in rat liver hepatocytes (≥70% of LBF in rats assuming 4.2 L/h/kg^15^) but moderately stable in human liver microsomes (LBF in human of ∼1.3 L/h/kg^16^) (Table 2). Permeability ranged from low (≤10 nm/s, **6c**) to favorable (≥70 nm/s, **6g**), and none of the compounds tested showed signs of P-glycoprotein (P-gp) mediated efflux (Table 2). Despite the improved human microsomal stability and permeability for pyridines **6g** and **6h**, these compounds did not lead to an improvement in rat hepatocyte stability or solubility compared to **BAY-4931**. Thus, **BAY-4931** was selected as the preferred compound for further optimization.

**Table 2:** ADME of Selected NCOR2-Interacting Ring Modifications

cmpd	Cl_h, mic_ (L/h/kg)^a^	Cl_r,hep_ (L/h/kg)^b^	GSH stability (% recovery at 1/2/4/24 h)^c^	Cys stability (% recovery at 1/2/4/24 h)^d^	Caco-2 permeability A–B (nm/s)/efflux ratio^e^	solubility (mg/L)^f^
**6a**	0.45	4.2	100/100/17/13	100/85/88/15	12/0.36	<0.1
**BAY-4931**	0.82	3.7	93/76/68/21	87/78/67/12	1.34/0	0.27
**6f**	0.81	4.1	100	100	18/0.10	<0.1
**6g**	0.31	4.2	100	100	170/0.41	<0.1
**6h**	0.61	4.1				<0.1

a Human microsomal stability determined by the incubation of 1 μM compound with human liver microsomes for 1 h. Refer to the Experimental Section for assay conditions. Clearance value represents one experiment.

b Rat hepatocyte stability assay determined by the incubation of 1 μM compound with rat liver hepatocytes for 1.5 h. Refer to the Experimental Section for assay conditions. Clearance value represents one experiment.

c Compound stability in buffer containing 500 μM glutathione. Refer to the Experimental Section for assay conditions. % recovery represents one experiment.

d Compound stability in buffer containing 500 μM cysteine. Refer to the Experimental Section for assay conditions. % recovery represents one experiment.

e Caco-2 permeability assay. Refer to the Experimental Section for assay conditions. Permeability and efflux ratio represent a single experiment.

f Thermodynamic solubility of compound in pH 6.5 PBS buffer from a DMSO stock. Refer to the Experimental Section for assay conditions.

We next turned our attention to the potentially solvent exposed central core to improve the physicochemical and ADME properties. Compounds with various central cores were synthesized according to Scheme 1b.

Inverting the benzoxazole regiochemistry (**8a**) retained corepressor recruitment, whereas making the benzoxazole the NCOR-interactor and the aryl the linker ablated corepressor recruitment (**8g** and **8h**) (Table 3). Benzannulated heteroaryl cores provided mixed results, as N-Me benzimidazoles reduced corepressor recruitment (**8b** and **8c**) while an imidazo-pyridine and a benzotriazole retained corepressor recruitment (**8d** and **8e**, respectively) (Table 3). Efforts to replace the amide linker with a sulfonamide failed to retain the activity (**8f**). Modifications that retained the biochemical activity resulted in unacceptable losses in efficacy and potency in the cellular assays.

**Table 3:**
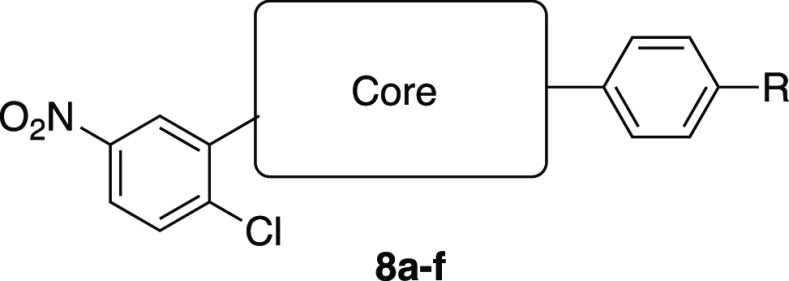

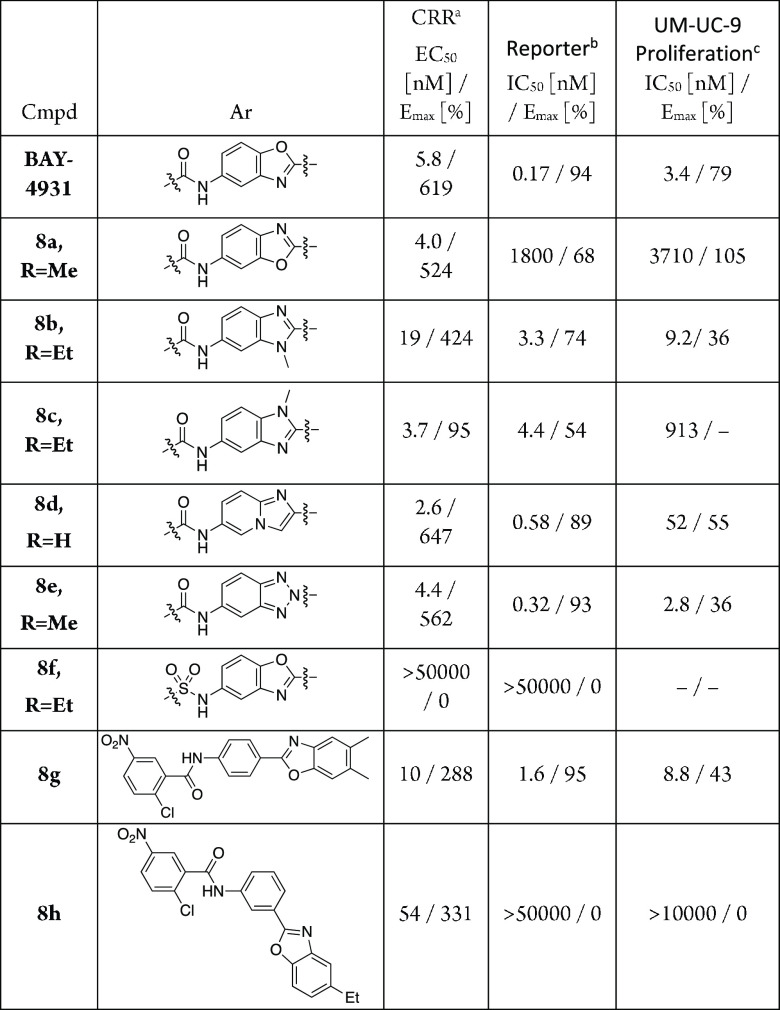
Central Core SAR

a LanthaScreen TR-FRET PPARγ corepressor recruitment assay with the NCOR2 (Smrt ID2) peptide. Refer to the Experimental Section for assay conditions. EC_50_ and *E*_max_ values represent the mean from at least two experiments, with points tested in duplicate.

b RT112-FABP4-NLucP cellular reporter assay for the transactivation of PPARγ. Refer to the Experimental Section for assay conditions. IC_50_ and *E*_max_ values represent the mean from two or more experiments, with points tested in quadruplicate.

c UM-UC-9 proliferation assay. Refer to the Experimental Section for assay conditions. IC_50_ and *E*_max_ values represent mean one or more experiments read out at day 7.

**Figure 5:**
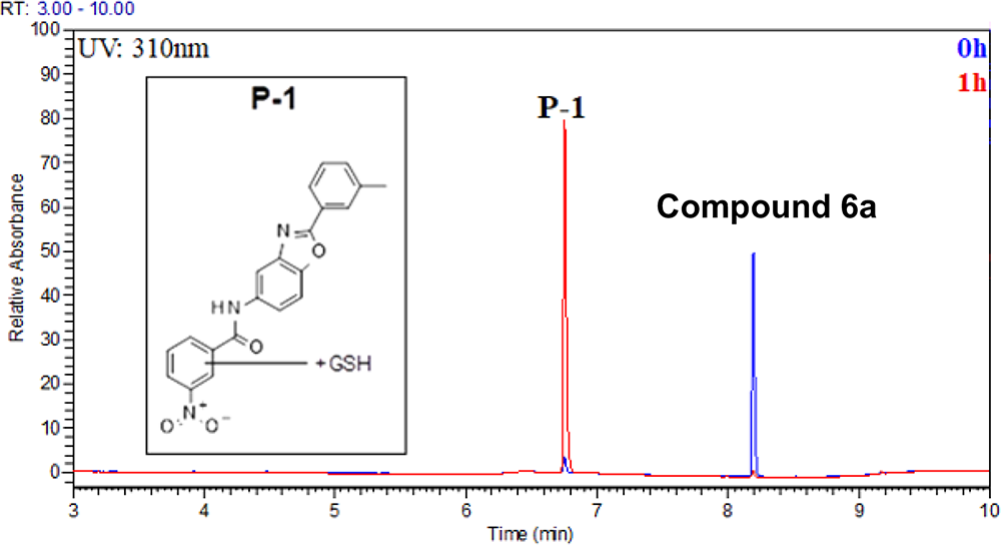
Identification of metabolic reaction products of compound **6a** by incubation with rat hepatocytes. UV chromatogram of **6a** and its metabolite P-1 before (blue) and after 1 h incubation (red) in rat hepatocytes and proposed structure of the metabolite P-1 based on the exact masses of the metabolite P-1 and its MS-MS fragments

Despite the lack of improvement in cellular assays, selected compounds from Table 3 were profiled in tier 1 ADME to understand the impact of increased polarity on pharmacokinetic properties. Notably, the more polar *N*-heteroaryl cores failed to improve the solubility or metabolic stability of the parent compound, though **8c** and **8d** demonstrated improved permeability (Table 4). Since no modifications made thus far improved the hepatocyte stability, compound **6a** was subjected to metabolite identification studies. The sole identified metabolite of **6a** results from GSH displacing the Ar–Cl warhead (Figure 5). Believing that improving the metabolic stability was essential to continue to progress the chemical series, we next modulated the reactivity of the covalent warhead.

**Table 4:** Tier 1 ADME of Selected Compounds from Table 3

cmpd	CL_b,hmic_ (L/h/kg)^a^	CL_b,rhep_ (L/h/kg)^b^	GSH stability (% remaining at 1/2/4/24 h)^c^	Cys stability (% remaining at 1/2/4/24 h)^d^	Caco-2 permeability A–B (nm/s)/efflux ratio^e^	solubility (mg/L)^f^
**BAY-4931**	0.82	3.7	93/76/68/21	87/78/67/12	1.3/0	0.27
**8b**	0.61	4.2	100/83/67/38	100/92/77/62		<0.1
**8c**	0.81	4.1	100	100	94/0.36	<0.1
**8d**	0.31	4.2	100	100	71/0.32	<0.1
**8e**	0.61	4.1	97/82/63/7.8	93/74/71/4.7	3.7/0	N/A

a Human microsomal stability determined by the incubation of 1 μM compound with human liver microsomes for 1 h. Refer to the Experimental Section for assay conditions. The clearance value represents one experiment.

b Rat hepatocyte stability assay determined by the incubation of 1 μM compound with rat liver hepatocytes for 1 h. Refer to the Experimental Section for assay conditions. The clearance value represents one experiment.

c Compound stability in buffer containing 500 μM glutathione. Refer to the Experimental Section for assay conditions. % recovery represents one experiment.

d Compound stability in buffer containing 500 μM cysteine. Refer to the Experimental Section for assay conditions. % recovery represents one experiment.

e Caco-2 permeability assay. Refer to the Experimental Section for assay conditions. Permeability and efflux ratio represent a single experiment.

f Thermodynamic solubility of the compound in pH 6.5 PBS buffer from a DMSO stock. Refer to the Experimental Section for assay conditions.

Less-reactive warheads decreased activity in PPARγ activity assays (Table 5). Changing the activating nitro group to a variety of less electron-withdrawing groups (**9a**–**d**) caused significant or full activity loss, though nitrile **9d** remained active in the corepressor recruitment assay. Removing or positionally shifting the covalent warhead resulted in inactive compounds (**9e** and **9f**, respectively). Warheads exploiting alternate chemistry, such as heteroarenes and acrylamides, were inactive (**9g** and **9h**, respectively). Aryl halides maintained *in vitro* activities similar to that of the parent chloride **BAY-4931** (**9i–k**). However, these substitutions failed to improve the metabolic stability (Table 6). Despite their poor aqueous solubility, low metabolic stability, and low permeability (see Table 6), chloride **BAY-4931** and bromide **9j** (**BAY-0069**) possessed the best overall *in vitro* profiles; thus, both were characterized *in vivo*.

**Table 5:**
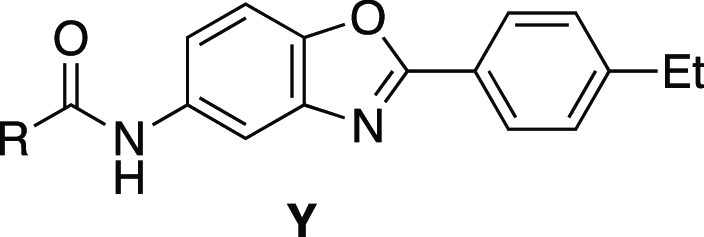

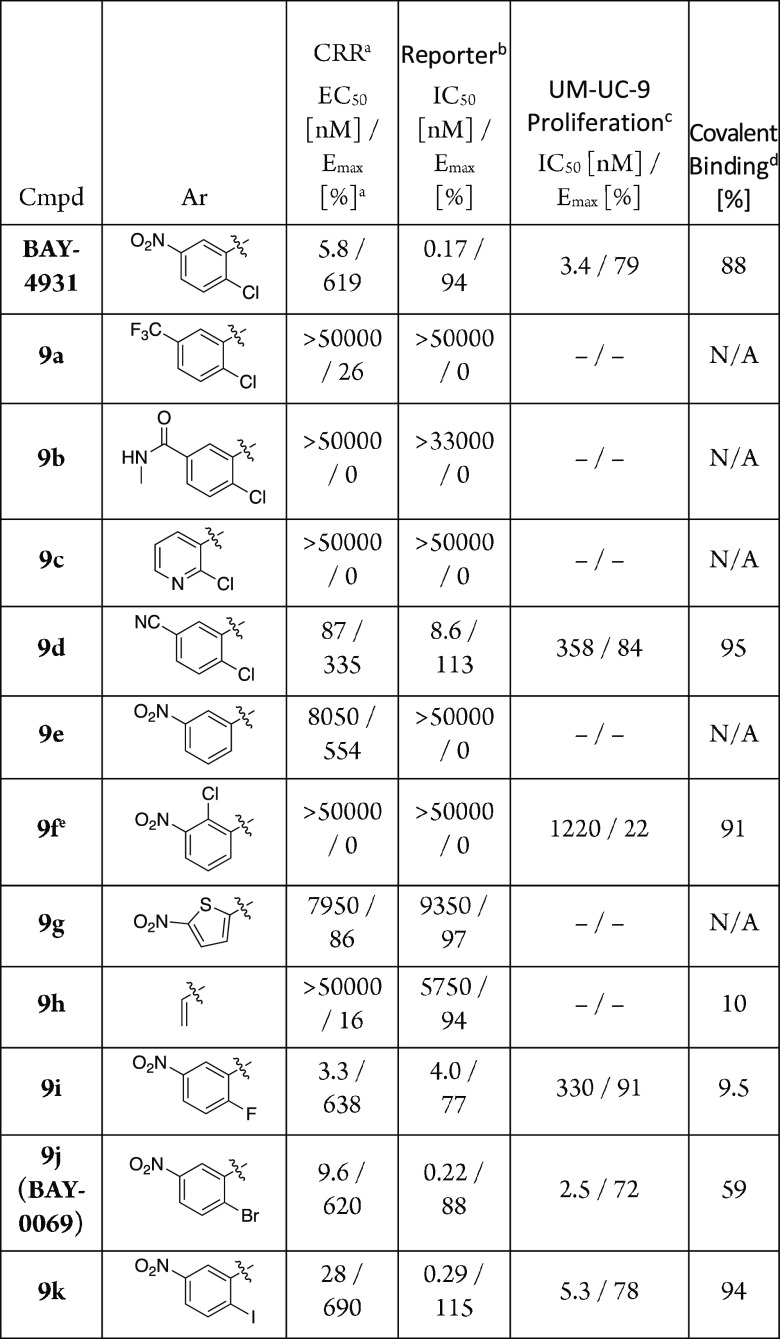
Warhead SAR

a CRR LanthaScreen TR-FRET PPARγ:NCOR2 assay with the NCOR2 (Smrt ID2) peptide. Refer to the Experimental Section for assay conditions. EC50 and *E*_max_ values represent the mean from at least two experiments.

b RT112-FABP4-NLucP cellular reporter assay for the transactivation of PPARγ. Refer to the Experimental Section for assay conditions. IC_50_ and *E*_max_ values represent the mean from at least two experiments.

c UM-UC-9 proliferation assay. Refer to the Experimental Section for assay conditions. IC_50_ and *E*_max_ values represent the mean of one or more experiments.

d Relative covalent binding assay. Refer to the Experimental Section for assay conditions. % represents relative amount of protein that showed a covalent adduct MW shift.

e With the 3-methylphenyl NCOR2-interacting ring.

**Table 6:** ADME Profile of Selected Warheads

cmpd	CL_b,hmic_ (L/h/kg)^a^	CL_b,rhep_ (L/h/kg)^b^	GSH stability (% remaining at 1/2/4/24 h)^c^	Cys stability (% remaining at 1/2/4/24 h)^d^	Caco-2 permeability A–B (nm/s)/efflux ratio^e^	solubility (mg/L)^f^
**BAY-4931**	0.82	3.7	93/76/68/21	87/78/67/12	1.3/0	0.27
**9d**	0.80	3.5			0/0	<0.1
**9i**	0.78	3.9	73/36/17/3.7	100/100/78/52		<0.1
**BAY-0069**	0.47	3.9	62/36/17/3.7	84/46/12/–	2.6/0.24	<0.1
**9k**	0.67	3.9			3.3/0	<0.1

a Human microsomal stability determined by the incubation of 1 μM compound with human liver microsomes for 1 h. Refer to the Experimental Section for assay conditions. The clearance value represents one experiment.

b Rat hepatocyte stability assay determined by the incubation of 1 μM compound with rat liver hepatocytes for 1 h. Refer to the Experimental Section for assay conditions. The clearance value represents one experiment.

c Compound stability in buffer containing 500 μM glutathione. Refer to the Experimental Section for assay conditions. % recovery represents one experiment.

d Compound stability in buffer containing 500 μM cysteine. Refer to the Experimental Section for assay conditions. % recovery represents one experiment.

e Caco-2 permeability assay. Refer to the Experimental Section for assay conditions. Permeability and efflux ratio represent a single experiment.

f Thermodynamic solubility of the compound in pH 6.5 PBS buffer from a DMSO stock. Refer to the Experimental Section for assay conditions.

## Selectivity Profiles of BAY-4931 and BAY-0069

*PPARG* is a member of a family of lipid-activated nuclear receptors, which encompasses *PPARA* and *PPARD*, with additional high homology with PXR. As such, **BAY-4931** and **BAY-0069** were tested in cellular reporter activity assays for mouse Pparγ and human PPARγ to determine cross-species reactivity, as well as assays for PPARA, PPARD, and PXR to determine selectivity (Table 7). **BAY-0069** only acted as an inverse-agonist at PPARγ, with little activity at the homologous PXR. When tested for CYP inhibition across a panel of the most relevant CYP enzymes for drug–drug interactions, both **BAY-4931** and **BAY-0069** only inhibited CYP2C8 (Table 7).

**Table 7:** Selectivity and Metabolic Liability Profiles of **BAY-4931** and **BAY-0069**

	GAL4-NHR-FLUC IC_50_ (nM)/*E*_max_ (%)^a^				CYP inhibition IC_50_ (μM)^b^					
compound	mouse Pparγ	PPARγ	PPARA	PPARD	CYP1A2	CYP2C8	CYP2C9	CYP2D6	CYP3A4	PXR NOEL^c^ (μM)
**BAY-4931**	0.14/82	0.40/100	>50000/0	>50000/0	>10	7.0	>10	>10	>10	>50
**BAY-0069**	24/25	6.3/72	7500/63	9000/84	>5	4.3	>5	>5	>5	41

a GAL4-NHR-LBD one hybrid reporter assay with the GAL4 DNA binding domain fused to a nuclear hormone receptor ligand binding domain (NHR-LBD). Refer to the Experimental Section for assay conditions. IC_50_ and *E*_max_ (% inhibition of luciferase reporter activity) or activation (+) values are from one experiment with a 10 point dose–response curve, with four replicates per point for mouse Pparγ, human PPARγ, PPARA, and PPARD.

b IC50 (μM) values for CYP inhibition assays, with the specific isoform indicated. Data represent the mean from one experiment performed in duplicate.

c PXR (NR1I3) no-effect level IC_50_.

## *In Vitro* Cell Profiling

**Figure 6:**
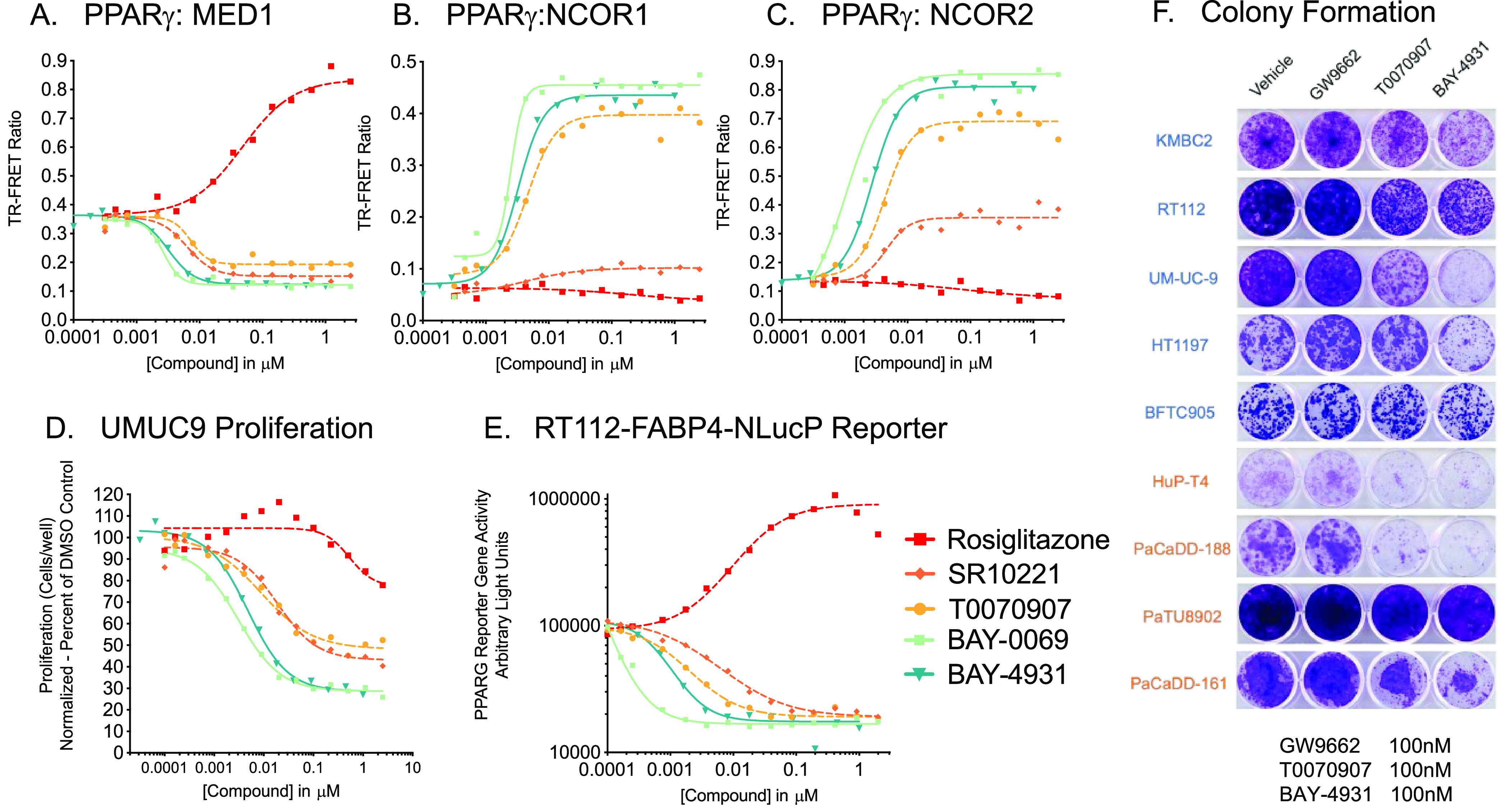
**BAY-4931** and **BAY-0069** are selective inverse-agonists of PPARγ. Biochemical TR-FRET evaluation of ligand-dependent changes in the interaction between the PPARγ ligand-binding domain and interacting peptide fragments from (A) coactivator MED1, (B) corepressor NCOR1, and (C) corepressor NCOR2. (D) Dose–response effects of the compound on the proliferation of UMUC9-H2B-GFP cells. (E) Dose–response effects of the compound on reporter activity in RT112-FABP4-NLucP. (F) Crystal violet colony formation assays comparing the effects of PPARγ modulators, with bladder cell lines highlighted in blue and pancreatic in orange. Samples were tested in triplicate, and representative wells are shown. See the Supporting Information for additional data.

Compounds **BAY-4931** and **BAY-0069** were compared to probe compounds T0070907 and SR10221 in dose–response tests across a variety of cell lines. These studies highlight subtle the differences of the *in vitro* profiles of these newly described compounds (Figure 6A–E). A dose-dependent increase in the interaction between PPARγ and corepressor peptides from NCOR1 and NCOR2 was observed as expected for inverse-agonists. A dose-dependent decrease in the interaction with the peptide from coactivator MED1 was also observed even in the absence of an exogenous agonist, indicating the destabilizing effect of inverse-agonists on basal interactions between PPARγ and coactivators. **BAY-4931** and **BAY-0069** led to antiproliferative effects in the *PPARG*-amplified cell line UM-UC-9, while agonist rosiglitazone led to a modest, but reproducible, increase in proliferation in this cell line.

During kinetic proliferation profiling in sensitive bladder cell lines,^3^ we noticed a delay of 2–5 days after initial dosing prior to the observation of changes in the proliferation rate and eventual cytostasis. Therefore, we tested the effects of T0070907 and **BAY-4931** on proliferation in a 12-day multiplexed cell line panel screen to screen for potentially sensitive cell lines (PRISM, Broad Institute, Figure S1).^17^ Candidate cell lines from this study and other *PPARG*-dependent cell lines from DepMap were selected for further evaluation in colony formation assays to enable extended treatment with compounds for 7–14 days (Figures 6F and S2). We compared the antiproliferative effects of inverse-agonists T0070907 and **BAY-4931** to the neutral antagonist GW9662. At a concentration of 100 nM, **BAY-4931** showed antiproliferative effects across the majority of the cell lines selected for predicted sensitivity. At the same concentration, T0070907 also showed antiproliferative effects in these cell lines, but to a more modest degree. UM-UC-9, HuP-T4, and PaCaDD-188 were among the most sensitive cell lines in the colony formation assays treated with **BAY-4931**. BFTC905, a bladder cell line, was chosen as a control, as it was not identified as sensitive to compounds in the PRISM panel or the genetic perturbation of *PPARG* in DepMap and compounds had minimal effect; taken together with antiproliferative effects in the predicted sensitive cell lines, the lack of growth inhibition in BFTC905 by the inverse-agonists suggests compound selectivity for the subset of *PPARG*-dependent cell lines.

## *In Vitro* Effects on Gene Expression

The effects of PPARγ modulators on global mRNA regulation were evaluated by RNA sequencing in the *RXRA* p.S427F hotspot mutation bladder cell line HT-1197 (Figure S3). Comparing treatment effects of inverse-agonist T0070907 at a cocentration of 500 nM to those of **BAY-4931** at a concentration of 200 nM, it was observed that the same genes were regulated by T0070907 and **BAY-4931**, with the same directionality of the effect. The gene expression effects anticorrelate with the effects of the PPARγ agonist rosiglitazone (Figure S3a). Furthermore, the entire gene set was regulated proportionately more with **BAY-4931** (Figure S3b) under the conditions tested. This indicates that at maximal receptor occupancy **BAY-4931** drives the equilibrium more toward repression than T0070907, which is analogous to expected effects of a partial agonist compared to a full agonist.

## *In Vivo* Profiling

**Figure 7:**
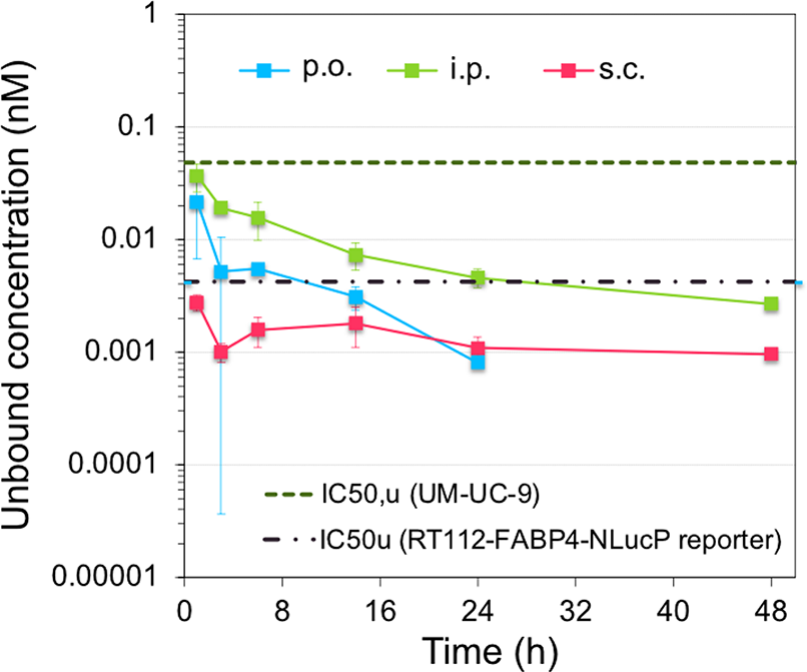
Exposure evaluation of **BAY-0069**. Unbound plasma concentrations of BAY-0069 after the administration of 100 mg/kg compound using *p.o.*, *i.p.*, and *s.c*. administration routes versus (*n* = 3 mice per group) *in vitro* antiproliferative IC_50_ (unbound) in UM-UC-9 cells and IC_50_ (unbound) in the RT112-FABP4-NLucP reporter assay. See Table S2 for detailed pharmacokinetic parameters.

Based on the strong *in vitro* effects and high selectivity of **BAY-0069** in addition to slightly improved microsomal stability over **BAY-4931** (Table 6), we elected to profile **BAY-0069***in vivo*. Unfortunately, the low intrinsic solubility of **BAY-0069** prevented profiling by *i.v*. administration; however, compound exposure was assessed by oral, intraperitoneal, and subcutaneous administration, all at 100 mg/kg (Figure 7). Despite the high dose administered, the obtained exposure was found to be very low by all three routes, with *i.p*. administration showing the best AUC_0-tlast_ of 0.26 h×mg/L and a *C*_max_ of 59 nM (see Table S2). Corrected for protein binding, the unbound *C*_max_ of **BAY-0069** covered the IC_50_ from the FABP4-NLucP reporter gene assay but failed to exceed the antiproliferative IC_50,u_ from UM-UC-9 cell lines, though intraperitoneal administration came closest with a *C*_max__,u_/IC_50__,u_ of 0.77.

**Figure 8:**
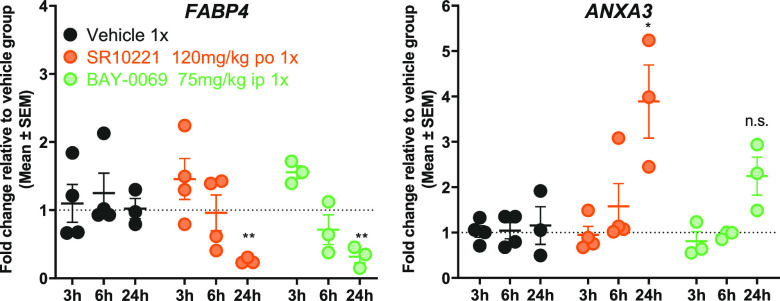
Pharmacodynamic regulation of PPARγ target gene expression in UM-UC-9 xenograft-bearing mice. The expression of *FABP4* and *ANXA3* was normalized to the housekeeping gene, *PPIA*, and reported as the normalized expression relative to the vehicle control. Mice were treated as indicated, and three or four were sacrificed at the respective time points. Statistical analysis was performed using one-way ANOVA with Dunnett’s multiple testing correction; *p ≤ 0.05, **p ≤ 0.01.

Despite this limited overall exposure, the *in vivo* effect of **BAY-0069** on gene expression was examined in RT112 xenograft tumors in comparison with the noncovalent inverse-agonist SR10221. We focused on *FABP4* and *ANXA3* gene expression as biomarkers of inverse agonism, as these PPARγ target genes showed strong regulatory effects in *in vitro* mRNA expression studies,^3^ with *FABP4* being strongly downregulated and *ANXA3* being upregulated by inverse-agonists. *In vivo*, **BAY-0069** demonstrated a modest downregulation of *FABP4* expression, comparable with that of SR10221, despite more robust *in vitro* inverse agonism in NCOR2 recruitment, RT112-FABP4-NLucP luciferase repression, and UM-UC-9 proliferation (Figure 8). Upregulation of *ANXA3* expression was statistically significant by SR10221 but not by **BAY-0069.** Note that for **BAY-0069** the maximum tolerable dose was reached and repeat dosing was not possible.

## Conclusions

Herein we disclose the discovery of a halo-nitroarene series of covalent PPARγ inverse-agonists. Chemical optimization through structure-informed design led to discovery of potent and selective PPARγ inverse-agonists. The extended benzoxazole core brought a lipophilic arene in close contact with the lipophilic surface of the corepressor NCOR2, while the binding of the chloro-nitroarene warhead sequestered Helix-12, an essential binding surface for the coactivator MED1, deep inside the protein.

Target engagement was demonstrated in a series of biochemical assays used to evaluate PPARγ binding and function. Cell-based assays confirmed that the compounds were cellularly active and modulated the function of PPARγ. Antiproliferative activity was observed in a panel of cell lines selected based on their genetic dependency to *PPARG* deletion.

The combination of poor solubility, low permeability, and rapid hepatic clearance by GSH adduct formation for **BAY-0069** resulted in a low exposure by all routes of administration. However, **BAY-4931** and **BAY-0069** show very robust *in vitro* effects in biochemical and cellular assays and provide novel tools for further studying PPARγ structure and function.

## Experimental Section

### General Chemical Methods and Materials

Commercially available reagents and anhydrous solvents were used as supplied without further purification. All air- and moisture-sensitive reactions were carried out in oven-dried (at 120 °C) glassware under an inert atmosphere of argon. A Biotage Initiator Classic microwave reactor was used for reactions conducted in a microwave oven. Reactions were monitored by TLC and UPLC analysis with a Waters Acquity UPLC-MS Single Quad system using an Acquity UPLC BEH C18 1.7 μm and 50 × 2.1 mm column. Basic conditions were as follows: eluent A, H_2_O + 0.2 vol % aq NH_3_ (32%); eluent B, MeCN; and gradient, 0–1.6 min 1–99% B and 1.6–2.0 min 99% B at a flow of 0.8 mL/min. Acidic conditions were as follows: eluent A, H_2_O + 0.1 vol % formic acid (99%), eluent B, MeCN; gradient, 0–1.6 min 1–99% B and 1.6–2.0 min 99% B at a flow of 0.8 mL/min; temperature, 60 °C; and DAD scan, 210–400 nm. Analytical TLC was carried out on aluminum-backed plates coated with Merck Kieselgel 60 F254, with visualization under UV light at 254 nm. Flash chromatography was carried out using a Biotage Isolera One system with 200–400 nm variable detector. Preparative HPLC was carried out with a Waters AutoPurification MS Single Quad system using a Waters XBridge C18 5 μm and 100 × 30 mm column. Basic conditions were as follows: eluent A, H_2_O + 0.2 vol % aq NH_3_ (32%); eluent B, MeCN; gradient, 0–0.5 min 5% B at a flow of 25 mL/min, 0.51–5.50 min 10–100% B at a flow of 70 mL/min, and 5.51–6.5 min 100% B at a flow of 70 mL/min. Acidic conditions were as follows: eluent A, H_2_O + 0.1 vol % formic acid (99%); eluent B, MeCN; gradient, 0–0.5 min 5% B at a flow of 25 mL/min, 0.51–5.50 min 10–100% B at a flow of 70 mL/min, and 5.51–6.5 min 100% B at a flow of 70 mL/min; temperature, 25 °C; and DAD scan, 210–400 nm. Regioisomers were separated by preparative chiral SFC using a Sepiatec Prep SFC100 insrtrument with a Chiralpak IA 5 μm and 250 × 30 mm column under the following conditions: eluent A, CO_2_; eluent B, methanol +0.2 vol % aq. NH_3_ (32%); isocratic, 35% B at a flow of 100 mL/min; temperature, 40 °C; BPR, 150 bar; and UV, 280 nm. Analytical chiral SFC was carried out using an Agilent 1260 Aurora SFC-module with a Chiralpak IA 5 μm 100 × 4.6 mm column under the following conditions: eluent A, CO_2_; eluent B, methanol +0.2 vol % aq NH3 (32%); isocratic, 35% B at a flow of 4 mL/min; temperature, 37.5 °C; BPR, 100 bar; and UV, 280 nm. NMR spectra were recorded at rt (22 ± 1 °C), unless otherwise noted, on Bruker Avance III HD spectrometers. ^1^H NMR spectra were obtained at 400 or 500 MHz and referenced to the residual solvent signal (7.26 ppm for CDCl_3_, 2.50 ppm for DMSO-*d*_6_). ^1^H NMR data are reported as follows: chemical shift (δ) in ppm, multiplicity (s = singlet, d = doublet, t = triplet, q = quartet, br = broad, and m = multiplet), integration, and assignment. Low-resolution mass spectra (electrospray ionization, ESI) were obtained via HPLC-MS (ESI) using a Waters Acquity UPLC system equipped with an SQ 3100 Mass Detector and an Acquity UPLC BEH C18 1.7 μm and 50 × 2.1 mm column. Basic conditions were as follows: eluent A, H_2_O + 0.2 vol % aq NH_3_ (32%); eluent B, MeCN; gradient, 0–1.6 min 1–99% B and 1.6–2.0 min 99% B at a flow of 0.8 mL/min. Acidic conditions were as follows: eluent A, H_2_O + 0.1 vol % formic acid (99%); eluent B, MeCN; gradient, 0–1.6 min 1–99% B and 1.6–2.0 min 99% B at a flow of 0.8 mL/min; temperature, 60 °C; and DAD scan, 210–400 nm. Low-resolution mass spectra were also obtained using an Agilent 1290 UPLCMS 6230 TOF system with a BEH C 18 1.7 μm and 50 × 2.1 mm column under the following conditions: eluent A, water + 0.05% formic acid (99%); eluent B, acetonitrile + 0.05% formic acid (99%); gradient, 0–1.7 2–90% B and 1.7–2.0 90% B at a flow of 1.2 mL/min; temperature, 60 °C; and DAD scan, 190–400 nm. Analysis and separation of mixtures of regioisomers were performed using chiral SFC on an Agilent 1260 instrument using Aurora SFC-Modul with a Chiralpak IA 5 μm and 100 × 4.6 mm column under the following conditions: eluent A, CO_2_; eluent B: Methanol + 0.2 vol % aqueous ammonia (32%); isocratic, 35% B at a flow of 4 mL/min; temperature, 37.5 °C; BPR, 100 bar; and UV, 280 nm. Analysis and separation of mixtures of regioisomers were also performed on a Sepiatec Prep SFC100 instrument with a Chiralpak IA 5 μm and 250 × 30 mm column under the following conditions: eluent A, CO_2_; eluent B: methanol + 0.2 vol % aqueous ammonia (32%); isocratic, 35% B at a flow of 100 mL/min; temperature, 40 °C; BPR, 150 bar; and UV, 280 nm. The purity of all target compounds was at least 95% as determined by UPLC-MS, with the exception of **6h** (93%). Compound names were generated using ICS software.

#### 2-(3-Methylphenyl)-5-nitro-1,3-benzoxazole (**6a-2**)

2-Amino-4-nitrophenol (1.80 g, 11.7 mmol) was dissolved in 90 mL of toluene, then to the solution was slowly added 1.0 equiv of 3-methylbenzoyl chloride (1.5 mL, 12 mmol). This mixture was refluxed for 20 h. Then, 0.25 equiv of *p*-toluenesulfonic acid (555 mg, 2.92 mmol) was added, and the mixture was refluxed for six more hours. The reaction mixture was allowed to cool to r.t. A dark precipitate formed that was collected by filtration and washed with toluene. The filtrate was evaporated under reduced pressure to give 3.54 g of the title compound (83% yield) as crude material, which was used directly in the next step.

^1^H NMR (400 MHz, DMSO-*d*_6_) δ ppm 8.67 (d, *J* = 2.28 Hz, 1 H), 8.35 (dd, *J* = 8.87, 2.28 Hz, 1 H), 8.01–8.10 (m, 3 H), 7.49–7.58 (m, 2 H), 2.45 (s, 3 H).

#### 2-(3-Methylphenyl)-1,3-benzoxazol-5-amine (**6a-1**)

2-(3-Methylphenyl)-5-nitro-1,3-benzoxazole (3.54 g, 13.9 mmol) was dissolved in 130 mL of ethanol, then to the solution was added 4.0 equiv of tin(II) chloride dihydrate (12.6 g, 55.6 mmol). This mixture was refluxed for 2 h. The mixture was adjusted with sodium carbonate solution (*w* = 10%) to pH 9 and then extracted three times with DCM, washed with water, dried over sodium sulfate, filtered, and evaporated to give 2.48 g (64% yield) of the crude material of the title compound, which was directly used in the next step.

UPLC-MS (Waters Acquity, acidic conditions): *t*_R_ = 0.98 min. MS (ESI+): *m*/*z* = 225.2 [M + H]^+^.

#### 2-Chloro-*N*-[2-(3-methylphenyl)-1,3-benzoxazol-5-yl]-5-nitrobenzamide (**6a**)

2-(3-Methylphenyl)-1,3-benzoxazol-5-amine (200 mg, 892 μmol), 1.2 equiv of 2-chloro-5-nitrobenzoic acid (216 mg, 1.07 mmol), and 1.3 equiv of propanephosphonic acid anhydride (*w* = 50% in DMF) were dissolved in 12 mL of DMF under an argon atmosphere. The mixture was stirred at r.t. for 18 h. DMF was evaporated under reduced pressure. Aqueous NaHCO_3_ solution and ethyl acetate were added to the mixture, and the layers were separated. The aqueous layer was extracted multiple times with ethyl acetate. The combined organic layers were washed with a saturated NaCl solution, dried over sodium sulfate, filtered, and evaporated to give the title compound as crude material. Purification by flash chromatography (silica gel, hexane/ethyl acetate gradient) gave 187 mg of the raw material. This material was suspended in DCM/MeOH (9:1), filtered, and washed again with DCM/MeOH (9:1) to give 31.8 mg (9% yield, 100% purity) of the title compound **6a**.

UPLC-MS (Waters Acquity, acidic conditions): *t*_R_ = 1.37 min. MS (ESI+): *m*/*z* = 408.1 [M + H]^+^.

^1^H NMR (400 MHz, DMSO-*d*_6_) δ ppm 10.91 (s, 1 H), 8.53 (d, *J* = 2.79 Hz, 1 H), 8.36 (dd, *J* = 8.87, 2.79 Hz, 1 H), 8.24 (d, *J* = 1.77 Hz, 1 H), 8.05 (s, 1 H), 7.98–8.03 (m, 1 H), 7.92 (d, *J* = 8.87 Hz, 1 H), 7.80 (d, *J* = 8.87 Hz, 1 H), 7.66 (dd, *J* = 8.87, 2.03 Hz, 1 H), 7.44–7.55 (m, 2 H), 2.44 (s, 3 H).

#### *tert*-Butyl [2-(2-Methylphenyl)-1,3-benzoxazol-5-yl]carbamate (**6b-2**)

A mixture of 5-bromo-2-(2-methylphenyl)-1,3-benzoxazole (1.02 g, 3.54 mmol), 1.2 equiv of *tert*-butyl carbamate (498 mg, 4.25 mmol), 0.1 equiv of *tert*-butyl-XPhos (150 mg, 354 μmol), 0.03 equiv of bis(dibenzylidenaceton)palladium(0) (61 mg, 110 μmol), and 2.0 equiv of sodium *tert*-butylate (680 mg, 7.08 mmol) in 180 mL of toluene was stirred at 60 °C for 8 days. To the mixture were added water and DCM. The phases were separated, and the organic phase was evaporated to dryness to give 1.28 g of the crude material. Purification by flash chromatography (silica gel, hexane/ethyl acetate gradient) gave 140 mg of the impure title compound (5% yield) alongside 983 mg of recovered starting material. The crude material was used without further purification in the next step.

UPLC-MS (Waters Acquity, acidic conditions): *t*_R_ = 1.48 min. MS (ESI+): *m*/*z* = 325.5 [M + H]^+^.

#### 2-(2-Methylphenyl)-1,3-benzoxazol-5-amine (**6b-1**)

A mixture of *tert*-butyl [2-(2-methylphenyl)-1,3-benzoxazol-5-yl]carbamate (156 mg, 480 μmol) and 1.2 mL of 4 M HCl in dioxane (10 equiv , 4.8 mmol) was stirred at r.t. for 18 h. The reaction mixture was evaporated to dryness to give 155.8 mg (36% yield) of the title compound, which was directly used in the next step.

UPLC-MS (Waters Acquity, acidic conditions): *t*_R_ = 0.95 min. MS (ESI+): *m*/*z* = 225.1 [M + H]^+^.

#### 2-Chloro-*N*-[2-(2-methylphenyl)-1,3-benzoxazol-5-yl]-5-nitrobenzamide (**6b**)

A mixture of 2-chloro-5-nitrobenzoic acid (145 mg, 719 μmol) and 20 equiv of thionyl chloride (1.0 mL, 14 mmol) was stirred at 80 °C for 2.5 h. The reaction mixture was evaporated to dryness and immediately used in the next step. A mixture of 2-(2-methylphenyl)-1,3-benzoxazol-5-amine (156 mg, 695 μmol), 1 equiv of 2-chloro-5-nitrobenzoyl chloride (153 mg, 695 μmol), and 5.0 equiv of triethylamine (480 μL, 3.5 mmol) in 3.2 mL of THF was stirred at r.t. for 4 days. The mixture was evaporated to dryness, and the remaining material was purified by preparative HPLC (acidic conditions) to give 21.0 mg (7% yield, 96% purity) of the title compound **6b**.

UPLC-MS (Waters Acquity, acidic conditions): *t*_R_ = 1.3 min. MS (ESI+): *m*/*z* = 408.2 [M + H]^+^.

^1^H NMR (400 MHz, DMSO-*d*_6_) δ ppm 10.91 (s, 1 H), 8.53 (d, *J* = 2.79 Hz, 1 H), 8.37 (dd, *J* = 8.87, 2.79 Hz, 1 H), 8.27 (d, *J* = 2.03 Hz, 1 H), 8.15 (dd, *J* = 7.86, 1.27 Hz, 1 H), 7.92 (d, *J* = 8.87 Hz, 1 H), 7.81 (d, *J* = 8.87 Hz, 1 H), 7.66 (dd, *J* = 8.87, 2.03 Hz, 1 H), 7.41–7.56 (m, 3 H), 2.77 (s, 3 H).

#### 5-Bromo-2-(4-ethylphenyl)-1,3-benzoxazole (**6c-3**)

4-Ethylbenzoyl chloride (18 mL, 120 mmol) was dissolved in 200 mL of toluene, and to the solution was added 2.0 equiv of sodium bicarbonate dissolved in 200 mL water. Under vigorous stirring, 1.0 equiv of 2-amino-4-bromophenol (23.4 g, 125 mmol) were added portion-wise, and the mixture stirred overnight at r.t. Ethyl acetate was added, and the mixture extracted with water three times. The organic phase was evaporated to dryness and taken up again in 200 mL toluene. 1.0 equiv of *p*-toluenesulfonic acid (21.4 g, 125 mmol) was added, and the reaction mixture stirred overnight at 95 °C. After cooling to r.t., to the mixture was added MTBE, then the reaction mixture washed three times with an aqueous sodium bicarbonate solution (*w* = 10%). The organic phase was evaporated to dryness, and the resulting crude material by flash chromatography (silica gel, hexane/ethyl acetate gradient) to give 23.0 g (61% yield) of the title compound.

^1^H NMR (400 MHz, DMSO-*d*_6_) δ ppm 8.05–8.28 (m, 2 H), 7.95–8.05 (m, 1 H), 7.68–7.85 (m, 1 H), 7.51–7.62 (m, 1 H), 7.33–7.50 (m, 2 H), 2.62–2.87 (m, 2 H), 1.23 (s, 3 H).

#### *tert*-Butyl [2-(4-Ethylphenyl)-1,3-benzoxazol-5-yl]carbamate (**6c-2**)

A mixture of 5-bromo-2-(4-ethylphenyl)-1,3-benzoxazole (22.7 g, 75.1 mmol) and 1.2 equiv of *tert*-butyl carbamate (13.2 g, 113 mmol) in toluene was purged with nitrogen gas. 0.1 equiv of bis(dibenzylidenaceton)palladium(0) (4.32 g, 7.5 mmol), 0.3 equiv of *tert*-butyl-XPhos (9.57 g, 22.5 mmol), and 3.0 equiv of sodium *tert*-butylate (21.7 g, 225 mmol) were added and the reaction mixture stirred at 80 °C for 6 h. The reaction mixture was evaporated to dryness, then water and DCM were added and the phases were separated. The combined organic phases were evaporated to dryness again, and the resulting crude material purified by flash chromatography (silica gel, hexane/ethyl acetate gradient) to give 19.6 g (77% yield) of the title compound.

^1^H NMR (400 MHz, DMSO-*d*_6_) δ ppm 9.40–9.58 (m, 1 H), 8.03–8.17 (m, 2 H), 7.84–7.97 (m, 1 H), 7.59–7.69 (m, 1 H), 7.38–7.52 (m, 3 H), 2.63–2.77 (m, 2 H), 1.50 (s, 9 H), 1.19–1.26 (m, 3 H).

#### 2-(4-Ethylphenyl)-1,3-benzoxazol-5-amine (**6c-1**)

A mixture of *tert*-butyl [2-(4-ethylphenyl)-1,3-benzoxazol-5-yl]carbamate (7.20 g, 21.3 mmol) and 53 mL of a HCl solution (4 N in dioxane, 10.0 equiv) was stirred at r.t. for 16 h. The reaction mixture was evaporated to dryness. To the mixture were added a diluted aqueous NaOH solution and DCM. The phases were separated, and the aqueous phase extracted multiple time with DCM. The combined organic phases were again evaporated to dryness to give 4.90 g (97% yield) of the title compound.

UPLC-MS (Waters Acquity, acidic conditions): *t*_R_ = 1.10 min. MS (ESI+): *m*/*z* = 239.3 [M + H]^+^.

#### 2-Chloro-*N*-[2-(4-ethylphenyl)-1,3-benzoxazol-5-yl]-5-nitrobenzamide (**6c**, **BAY-4931**)

A mixture of 2-(4-ethylphenyl)-1,3-benzoxazol-5-amine (250 mg, 1.05 mmol), 1.5 equiv of 2-chloro-5-nitrobenzoic acid (317 mg, 1.57 mmol), 2 equiv of HATU (798 mg, 2.10 mmol), and 5.0 equiv of DIPEA (910 μL, 5.2 mmol) in 4.6 mL of DMF was stirred at r.t. °C for 12 h. Water and a saturated NaHCO_3_ solution were added, and the mixture extracted with ethyl acetate, washed with brine, dried via Na_2_SO_4_, and evaporated to dryness. The remaining crude material was purified by flash chromatography (silica gel, hexane/ethyl acetate gradient) to give 190 mg (43% yield, 100% purity) of the title compound **6c**.

^1^H NMR (400 MHz, DMSO-*d*_6_) δ ppm 10.90 (s, 1 H), 8.53 (d, J = 2.79 Hz, 1 H), 8.36 (dd, J = 8.87, 2.79 Hz, 1 H), 8.22 (d, J = 1.77 Hz, 1 H), 8.09–8.16 (m, 2 H), 7.92 (d, J = 8.87 Hz, 1 H), 7.79 (d, J = 8.87 Hz, 1 H), 7.65 (dd, J = 8.87, 2.03 Hz, 1 H), 7.47 (d, J = 8.62 Hz, 2 H), 2.72 (q, J = 7.60 Hz, 2 H), 1.24 (t, J = 7.60 Hz, 3 H).

UPLC-MS (Waters Acquity, acidic conditions): *t*_R_ = 1.42 min. MS (ESI+): *m*/*z* = 422.2 [M + H]^+^.

Refer to Figure S6 for the HPLC trace of **6c** (**BAY-4931**)

#### 5-Bromo-2-(3-chlorophenyl)-1,3-benzoxazole (**6d-3**)

A mixture of 2-amino-4-bromophenol (2.00 g, 10.6 mmol) and 1.2 equiv of 3-chlorobenzoyl chloride (2.05 g, 12 mmol) in 40 mL of toluene was stirred at 80 °C for 3 days. 1.0 equiv of *p*-toluenesulfonic acid (1.83 g, 10.6 mmol) was added, and the reaction mixture stirred another 3 days at 80 °C. The mixture was evaporated to dryness, then water and ethyl acetate added and the phases were separated. The aqueous phase was extracted multiple times with ethyl acetate. The combined organic phases were washed with a diluted aq NaOH solution, dried with Na_2_SO_4_, and evaporated to dryness again. The remaining crude material was purified by flash chromatography (silica gel, hexane/ethyl acetate gradient) to give 3.0 g (91% yield) of the title compound.

UPLC-MS (Waters Acquity, acidic conditions): *t*_R_ = 1.61 min. MS (ESI+): *m*/*z* = 310.0 [M + H]^+^.

#### *tert*-Butyl [2-(3-Chlorophenyl)-1,3-benzoxazol-5-yl]carbamate (**6d-2**)

A mixture of 5-bromo-2-(3-chlorophenyl)-1,3-benzoxazole (3.00 g, 9.72 mmol), 2.0 equiv of *tert*-butyl carbamate (2.28 g, 19.4 mmol), 0.1 equiv of bis(dibenzylidenaceton)palladium(0) (0.56 g, 0.97 mmol), 0.1 equiv of *tert*-butyl-XPhos (413 mg, 0.97 mmol), and 2.0 equiv of sodium *tert*-butylate (1.87 g, 19.4 mmol) in 220 mL of toluene was stirred at 90 °C for 6 days. The reaction mixture was evaporated to dryness, water and ethyl actetate were added, and the phases separated. The aqueous layer was extracted with ethyl acetate. The combined organic phases were washed with a saturated NaCl solution, dried over Na_2_SO_4_, and evaporated to dryness. The resulting crude material was purified by flash chromatography (silica gel, hexane/ethyl acetate gradient) to give 1.66 g (50% yield) of the title compound.

UPLC-MS (Waters Acquity, acidic conditions): *t*_R_ = 1.51 min. MS (ESI+): *m*/*z* = 345.2 [M + H]^+^.

#### 2-(3-Chlorophenyl)-1,3-benzoxazol-5-amine (**6d-1**)

A mixture of *tert*-butyl [2-(3-chlorophenyl)-1,3-benzoxazol-5-yl]carbamate (1.66 g, 4.81 mmol) and 30 mL of a HCl solution (4 N in dioxane, 25.0 equiv) was stirred at r.t. for 24 h. The reaction was mioxture was evaporated to dryness to give the title compound, which was used in the next step without further purification.

UPLC-MS (Waters Acquity, acidic conditions): *t*_R_ = 1.06 min. MS (ESI+): *m*/*z* = 246.0 [M + H]^+^.

#### 2-Chloro-*N*-[2-(3-chlorophenyl)-1,3-benzoxazol-5-yl]-5-nitrobenzamide (**6d**)

A mixture of 2-(3-chlorophenyl)-1,3-benzoxazol-5-amine (100 mg, 409 μmol), 1.4 equiv of 2-chloro-5-nitrobenzoic acid (115 mg, 572 μmol), 1.3 equiv of HATU (202 mg, 531 μmol), and 3.0 equiv of DIPEA (210 μL, 1.2 mmol) in 1.8 mL of DMF was stirred at r.t. until complete conversion. To the mixture were added water and ethyl acetate, and the phases separated. The aqueous phase was extracted multiple times with ethyl acetate, washed with a saturated NaCl solution, dried with Na_2_SO_4_, and evaporated to dryness. The remaining crude material was purified by preparative HPLC (acidic conditions) to give 29 mg (16% yield, 98% purity) of the title compound **6d**.

UPLC-MS (Waters Acquity, acidic conditions): *t*_R_ = 1.37 min. MS (ESI+): *m*/*z* = 428.0 [M + H]^+^.

^1^H NMR (400 MHz, DMSO-*d*_6_) δ ppm 10.94 (s, 1 H), 8.53 (d, *J* = 2.79 Hz, 1 H), 8.37 (dd, *J* = 8.87, 2.79 Hz, 1 H), 8.27 (d, *J* = 1.77 Hz, 1 H), 8.15–8.21 (m, 2 H), 7.92 (d, *J* = 8.87 Hz, 1 H), 7.83 (d, *J* = 8.87 Hz, 1 H), 7.64–7.76 (m, 3 H).

#### *N*-[2-(2-Bromophenyl)-1,3-benzoxazol-5-yl]-2-chloro-5-nitrobenzamide (**6e**)

UPLC-MS (Waters Acquity, acidic conditions): *t*_R_ = 1.34 min. MS (ESI+): *m*/*z* = 474.2 [M + H]^+^, 100% purity.

^1^H NMR (400 MHz, DMSO-*d*_6_) δ ppm 10.95 (s, 1 H), 8.54 (d, *J* = 2.53 Hz, 1 H), 8.37 (dd, *J* = 8.62, 2.79 Hz, 1 H), 8.30 (d, *J* = 1.77 Hz, 1 H), 8.10 (dd, *J* = 7.73, 1.65 Hz, 1 H), 7.89–7.95 (m, 2 H), 7.85 (d, *J* = 8.87 Hz, 1 H), 7.70 (dd, *J* = 8.87, 2.03 Hz, 1 H), 7.54–7.66 (m, 2 H).

#### 5-Bromo-2-(3-fluoro-4-methoxyphenyl)-1,3-benzoxazole (**6f-3**)

A mixture of 3-fluoro-4-methoxybenzoic acid (2.17 g, 12.8 mmol) and 10 equiv of SOCl_2_ (9.3 mL, 130 mmol) was stirred at 60 °C for 3 h. The mixture was evaporated to dryness, and the resulting acid chloride used directly in the next step. A mixture of 2-amino-4-bromophenol (2.39 g, 12.7 mmol) and 1.0 equiv of 3-fluoro-4-methoxybenzoyl chloride (2.40 g, 12.7 mmol) in 100 mL of toluene was stirred at 80 °C for 16 h. 0.6 equiv of *p*-toluenesulfonic acid (1.31 g, 7.64 mmol) was added, and the reaction mixture stirred overnight at 80 °C. The mixture was evaporated to dryness, and the remaining crude material was purified by flash chromatography (silica gel, hexane/ethyl acetate gradient) to give 2.13 g (52% yield) of the title compound.

UPLC-MS (Waters Acquity, acidic conditions): *t*_R_ = 1.48 min. MS (ESI+): *m*/*z* = 324.0 [M + H]^+^.

#### *tert*-Butyl [2-(3-Fluoro-4-methoxyphenyl)-1,3-benzoxazol-5-yl]carbamate (**6f-2**)

A mixture of 5-bromo-2-(3-fluoro-4-methoxyphenyl)-1,3-benzoxazole (4.00 g, 12.4 mmol), 2.0 equiv of *tert*-butyl carbamate (2.91 g, 24.8 mmol), 0.1 equiv of Bis(dibenzylidenaceton)palladium(0) (0.71 g, 1.24 mmol), 0.1 equiv of *tert*-butyl-XPhos (527 mg, 1.24 mmol), and 2.0 equiv of sodium *tert*-butylate (2.39 g, 24.8 mmol) in 280 mL of toluene was stirred at 90 °C for 6 days. The reaction mixture was evaporated to dryness, water and ethyl actetate were added, and the phases were separated. The aqueous layer was extracted with ethyl acetate. The combined organic phases were washed with a saturated NaCl solution, dried over Na_2_SO_4_, and evaporated to dryness. The resulting crude material was purified by flash chromatography (silica gel, hexane/ethyl acetate gradient) to give 2.14 g (48% yield) of the title compound.

UPLC-MS (Waters Acquity, acidic conditions): *t*_R_ = 1.37 min. MS (ESI+): *m*/*z* = 359.4 [M + H]^+^.

#### 2-(3-Fluoro-4-methoxyphenyl)-1,3-benzoxazol-5-amine (**6f-1**)

A mixture of *tert*-butyl [2-(3-fluoro-4-methoxyphenyl)-1,3-benzoxazol-5-yl]carbamate (611 mg, 1.70 mmol) and 15 mL of a HCl solution (4 N in dioxane, 35.0 equiv) was stirred at r.t. until complete conversion. The reaction was evaporated to dryness. To the mixture were added 2 M aq NaOH solution and DCM. The phases were separated, and the aqueous phase was extracted multiple time with DCM. The combined organic phases were again evaporated to dryness to give 464 mg of the title compound.

UPLC-MS (Waters Acquity, acidic conditions): *t*_R_ = 0.89 min. MS (ESI+): *m*/*z* = 259.1 [M + H]^+^.

^1^H NMR (400 MHz, DMSO-*d*_6_) δ ppm 7.91–7.97 (m, 1 H), 7.88 (dd, *J* = 12.04, 2.15 Hz, 1 H), 7.34–7.41 (m, 2 H), 6.84 (d, *J* = 2.03 Hz, 1 H), 6.65 (dd, *J* = 8.62, 2.28 Hz, 1 H), 5.11 (s, 2 H), 3.94 (s, 3 H).

#### 2-Chloro-*N*-[2-(3-fluoro-4-methoxyphenyl)-1,3-benzoxazol-5-yl]-5-nitrobenzamide (**6f**)

A mixture of 2-(3-fluoro-4-methoxyphenyl)-1,3-benzoxazol-5-amine (92.0 mg, 356 μmol), 1.3 equiv of 2-chloro-5-nitrobenzoic acid (93.3 mg, 463 μmol), 2.0 equiv of PyBrop (332 mg, 712 μmol), and 5.0 equiv of DIPEA (310 μL, 1.8 mmol) in 2.9 mL of DMF was stirred at r.t. for 3 days. To the mixture were added water and DCM. The phases were separated, and the aqueous phase extracted multiple times with DCM. The combined organic phases were evaporated to dryness. The remaining crude material was purified by preparative HPLC (basic conditions) to give 50 mg (32% yield, 96% purity) of the title compound **6f**.

UPLC-MS (Waters Acquity, basic conditions): *t*_R_ = 1.24 min. MS (ESI+): *m*/*z* = 442.0 [M + H]^+^.

^1^H NMR (400 MHz, DMSO-*d*_6_) δ ppm 10.90 (s, 1 H), 8.53 (d, *J* = 2.79 Hz, 1 H), 8.36 (dd, *J* = 8.62, 2.79 Hz, 1 H), 8.21 (d, *J* = 1.77 Hz, 1 H), 7.95–8.05 (m, 2 H), 7.92 (d, *J* = 8.87 Hz, 1 H), 7.78 (d, *J* = 8.87 Hz, 1 H), 7.65 (dd, *J* = 8.87, 2.03 Hz, 1 H), 7.42 (t, *J* = 8.74 Hz, 1 H), 3.96 (s, 3 H).

#### 5-Bromo-2-(5-methylpyridin-3-yl)-1,3-benzoxazole (**6g-3**)

A mixture of 5-methylpyridine-3-carboxylic acid (3.91 g, 28.5 mmol) and 10 equiv of (21 mL, 290 mmol) was stirred at 60 °C for 2 days. The mixture was evaporated to dryness, and the resulting acid chloride used directly in the next step. A mixture of 2-amino-4-bromophenol (5.30 g, 28.2 mmol) and 1.0 equiv of 5-methylpyridine-3-carbonyl chloride (4.39 g, 28.2 mmol) in 110 mL of toluene was stirred at 80 °C for 6 days. 0.6 equiv of *p*-toluenesulfonic acid (2.91 g, 16.9 mmol) was added, and the reaction mixture stirred for 3 days at 80 °C. The mixture was evaporated to dryness. To the mixture were added water, 31 mL of a 2N NaOH solution, and DCM were added, the phases were separated. The organic phase was evaporated to dryness, and the remaining crude material was purified by flash chromatography (silica gel, hexane/ethyl acetate gradient) to give 200 mg (2.5% yield) of the title compound alongside 2.5 g of recovered aminophenol starting material.

UPLC-MS (Waters Acquity, acidic conditions): *t*_R_ = 1.27 min. MS (ESI+): *m*/*z* = 291.0 [M + H]^+^.

#### *tert*-Butyl [2-(5-Methylpyridin-3-yl)-1,3-benzoxazol-5-yl]carbamate (**6g-2**)

A mixture of 5-bromo-2-(5-methylpyridin-3-yl)-1,3-benzoxazole (550 mg, 1.90 mmol), 1.2 equiv of *tert*-butyl carbamate (267 mg, 2.28 mmol), 0.03 equiv of bis(dibenzylidenaceton)palladium(0) (33 mg, 57 μmol), 0.1 equiv of *tert*-butyl-XPhos (80.8 mg, 190 μmol), and 2.0 equiv of sodium *tert*-butylate ((366 mg, 3.80 mmol) in 94 mL of toluene was stirred at 90 °C for 4 h. The reaction mixture was evaporated to dryness, water and DCM were added, and the phases were separated. The aqueous layer was extracted multiple times with DCM. The combined organic phases were evaporated to dryness. The resulting crude material was purified by flash chromatography (silica gel, hexane/ethyl acetate gradient) to give 500 mg (81% yield) of the title compound.

UPLC-MS (Waters Acquity, acidic conditions): *t*_R_ = 1.20 min. MS (ESI+): *m*/*z* = 326.5 [M + H]^+^.

#### 2-(5-Methylpyridin-3-yl)-1,3-benzoxazol-5-amine (**6g-1**)

A mixture *tert*-butyl [2-(5-methylpyridin-3-yl)-1,3-benzoxazol-5-yl]carbamate (500 mg, 1.54 mmol) and 3.8 mL of a HCl solution (4 N in dioxane, 10.0 equiv) was stirred at r.t. until complete conversion. To the mixture were added water and DCM, and the phases were separated. The aqueous phase was extracted multiple time with DCM. The combined organic phases were again evaporated to dryness to give 300 mg (87% yield) of the title compound.

UPLC-MS (Waters Acquity, acidic conditions): *t*_R_ = 0.66 min. MS (ESI+): *m*/*z* = 226.2 [M + H]^+^.

#### 2-Chloro-*N*-[2-(5-methylpyridin-3-yl)-1,3-benzoxazol-5-yl]-5-nitrobenzamide (**6g**)

A mixture of 2-(5-methylpyridin-3-yl)-1,3-benzoxazol-5-amine (41.0 mg, 182 μmol), 1.3 equiv of 2-chloro-5-nitrobenzoic acid (47.7 mg, 237 μmol), 2.0 equiv of PyBrop (170 mg, 364 μmol), 4.0 equiv of DIPEA (130 μL, 730 μmol), and 0.05 equiv of 4-dimethylaminopyridine (1.11 mg, 9.10 μmol) in 0.8 mL of DMF was stirred at r.t. until complete conversion. The reaction mixture was evaporated to dryness. The remaining crude material was purified by flash chromatography (silica gel, hexane/ethyl actetate gradient) to give 30 mg (36% yield, 100% purity) of the title compound **6g**.

LC-MS (Agilent, acidic conditions): *t*_R_ = 1.05 min. MS (ESI+): *m*/*z* = 409.1 [M + H]^+^.

^1^H NMR (400 MHz, DMSO-*d*_6_) δ ppm 10.94 (s, 1 H), 9.17 (d, *J* = 1.77 Hz, 1 H), 8.66 (d, *J* = 1.27 Hz, 1 H), 8.54 (d, *J* = 2.79 Hz, 1 H), 8.33–8.42 (m, 2 H), 8.28 (d, *J* = 1.77 Hz, 1 H), 7.92 (d, *J* = 8.87 Hz, 1 H), 7.84 (d, *J* = 8.87 Hz, 1 H), 7.69 (dd, *J* = 8.87, 2.03 Hz, 1 H), 2.45 (s, 3 H).

#### *N*-(5-Bromo-2-hydroxyphenyl)-6-ethylpyridine-3-carboxamide (**6h-4**)

A mixture of 2-amino-4-bromophenol (2.00 g, 10.6 mmol), 1.2 equiv of 6-ethylpyridine-3-carboxylic acid (1.93 g, 12.8 mmol), 1.5 equiv of HATU (6.07 g, 16.0 mmol), and 5.0 equiv of TEA (7.4 mL, 53 mmol) in 50 mL DMF was stirred at r.t. for 2 h. DMF was evaporated under reduced pressure. To the mixture were added ater and DCM, and the phases were separated. The aqueous layer was extracted multiple times with DCM. The combined organic phases were evaporated to dryness, and the remaining crude material was purified by flash chromatography (silica gel, hexane/ethyl acetate gradient) to give 1.80 g (53% yield) of the title compound.

LC-MS (Agilent, acidic conditions): *t*_R_ = 0.86 min. MS (ESI+): *m*/*z* = 321.1 [M + H]^+^.

^1^H NMR (400 MHz, DMSO-*d*_6_) δ ppm 10.13 (s, 1 H), 9.70 (s, 1 H), 9.01 (d, *J* = 1.77 Hz, 1 H), 8.21 (dd, *J* = 8.11, 2.28 Hz, 1 H), 7.88 (d, *J* = 2.28 Hz, 1 H), 7.42 (d, *J* = 8.11 Hz, 1 H), 7.21 (dd, *J* = 8.62, 2.53 Hz, 1 H), 6.88 (d, *J* = 8.62 Hz, 1 H), 2.83 (q, *J* = 7.60 Hz, 2 H), 1.25 (t, *J* = 7.60 Hz, 3 H).

#### 5-Bromo-2-(6-ethylpyridin-3-yl)-1,3-benzoxazole (**6h-3**)

A mixture of *N*-(5-bromo-2-hydroxyphenyl)-6-ethylpyridine-3-carboxamide (1.8 g, 5.60 mmol) and 15 mL of polyphosphoric acid was stirred at 200 °C for 2 h. After cooling to room temperature, to the mixture were added ice water and DCM, and the phases were separated. The aqueous layer was extracted multiple times with DCM. The combined organic phases were evaporated to dryness to give 1.40 g (82% yield) of the title compound, which was used without further purification in the next step.

LC-MS (Agilent, acidic conditions): *t*_R_ = 1.27 min. MS (ESI+): *m*/*z* = 303.1 [M + H]^+^.

^1^H NMR (400 MHz, DMSO-*d*_6_) δ ppm 9.24 (dd, *J* = 2.28, 0.76 Hz, 1 H), 8.43 (dd, *J* = 8.11, 2.28 Hz, 1 H), 8.08 (d, *J* = 2.03 Hz, 1 H), 7.81 (d, *J* = 8.62 Hz, 1 H), 7.61 (dd, *J* = 8.74, 1.90 Hz, 1 H), 7.53 (d, *J* = 7.86 Hz, 1 H), 2.87 (q, *J* = 7.60 Hz, 2 H), 1.28 (t, *J* = 7.60 Hz, 3 H).

#### *tert*-Butyl [2-(6-Ethylpyridin-3-yl)-1,3-benzoxazol-5-yl]carbamate (**6h-2**)

A mixture of 5-bromo-2-(6-ethylpyridin-3-yl)-1,3-benzoxazole (1400 mg, 4.62 mmol), 1.5 equiv of *tert*-butyl carbamate (812 mg, 6.92 mmol), 0.1 equiv of Bis(dibenzylidenaceton)palladium(0) (266 mg, 462 μmol), 0.3 equiv of *tert*-butyl-XPhos (588 mg, 1.39 mmol), and 3.0 equiv of sodium *tert*-butylate (1.33 g, 13.86 mmol) in 38 mL of toluene was stirred at 90 °C for 3 h. The reaction mixture was evaporated to dryness, water and DCM were added, and the phases were separated. The aqueous layer was extracted multiple times with DCM. The combined organic phases were evaporated to dryness. The resulting crude material was purified by flash chromatography (silica gel, hexane/ethyl acetate gradient) to give 1.30 g (83% yield) of the title compound.

UPLC-MS (Waters Acquity, acidic conditions): *t*_R_ = 1.27 min. MS (ESI+): *m*/*z* = 340.6 [M + H]^+^.

#### 2-(6-Ethylpyridin-3-yl)-1,3-benzoxazol-5-amine (**6h-1**)

A mixture of *tert*-butyl [2-(6-ethylpyridin-3-yl)-1,3-benzoxazol-5-yl]carbamate (1.30 g, 3.83 mmol) and 9.6 mL of a HCl solution (4 N in dioxane, 10.0 equiv) was stirred at r.t. until complete conversion. To the mixture were added water, a saturated NaHCO_3_ solution, and DCM, and the phases were separated. The aqueous phase was extracted multiple times with DCM. The combined organic phases were again evaporated to dryness to give 915 mg of the title compound, which was used without further purification in the next step.

UPLC-MS (Waters Acquity, acidic conditions): *t*_R_ = 0.76 min. MS (ESI+): *m*/*z* = 240.3 [M + H]^+^.

#### 2-Chloro-*N*-[2-(6-ethylpyridin-3-yl)-1,3-benzoxazol-5-yl]-5-nitrobenzamide (**6h**)

A mixture of 2-(6-ethylpyridin-3-yl)-1,3-benzoxazol-5-amine (88.0 mg, 368 μmol), 1.1 equiv of 2-chloro-5-nitrobenzoic acid (81.5 mg, 405 μmol), 5.0 equiv of TEA (260 μL, 1.8 mmol), and 2.0 equiv of HATU (280 mg, 0.74 mmol) in 4.4 mL of DMF was stirred at r.t. until complete conversion. The reaction mixture was evaporated to dryness and purified by preparative HPLC (acidic conditions) to give 50.0 mg (29% yield, 93% purity) of the title compound **6h**.

LC-MS (Agilent, acidic conditions): *t*_R_ = 1.14 min. MS (ESI+): *m*/*z* = 423.1 [M + H]^+^.

^1^H NMR (400 MHz, DMSO-*d*_6_) δ ppm 10.93 (s, 1 H), 9.23–9.29 (m, 1 H), 8.53 (d, *J* = 2.79 Hz, 1 H), 8.45 (dd, *J* = 8.24, 2.41 Hz, 1 H), 8.36 (dd, *J* = 8.87, 2.79 Hz, 1 H), 8.26 (d, *J* = 1.77 Hz, 1 H), 7.92 (d, *J* = 8.62 Hz, 1 H), 7.83 (d, *J* = 8.87 Hz, 1 H), 7.69 (dd, *J* = 8.87, 2.03 Hz, 1 H), 7.54 (d, *J* = 8.36 Hz, 1 H), 2.88 (q, *J* = 7.60 Hz, 2 H), 1.29 (t, *J* = 7.60 Hz, 3 H).

#### 2-Chloro-*N*-[2-(4-methylphenyl)-1,3-benzoxazol-6-yl]-5-nitrobenzamide (**8a**)

A mixture of 2-(4-methylphenyl)-1,3-benzoxazol-6-amine (80.0 mg, 357 μmol), 1.3 equiv of 2-chloro-5-nitrobenzoic acid (93.5 mg, 464 μmol), 2.0 equiv of PyBrop (333 mg, 713 μmol), and 5.0 equiv of DIPEA (310 μL, 1.8 mmol) in 2.7 mL of DMF was stirred at r.t. until complete conversion. The reaction mixture was evaporated to dryness. The remaining crude material was purified by preparative HPLC (acidic conditions) to give 38 mg (25% yield, 96% purity) of the title compound **8a**.

UPLC-MS (Waters Acquity, acidic conditions): *t*_R_ = 1.38 min. MS (ESI+): *m*/*z* = 407.9 [M + H]^+^.

^1^H NMR (400 MHz, DMSO-*d*_6_) δ ppm 11.02 (s, 1 H), 8.53 (d, *J* = 2.79 Hz, 1 H), 8.37 (dd, *J* = 8.87, 2.79 Hz, 1 H), 8.32 (d, *J* = 1.77 Hz, 1 H), 8.10 (d, *J* = 8.11 Hz, 2 H), 7.92 (d, *J* = 8.87 Hz, 1 H), 7.79 (d, *J* = 8.87 Hz, 1 H), 7.56 (dd, *J* = 8.74, 1.90 Hz, 1 H), 7.44 (d, *J* = 8.11 Hz, 2 H), 2.42 (s, 3 H).

#### 5-Bromo-2-(4-ethylphenyl)-1*H*-benzimidazole (**8b-4** and **8c-4**)

A mixture of 4-bromobenzene-1,2-diamine (5.44 g, 29.1 mmol), 1.1 equiv of 4-ethylbenzaldehyde (4.29 g, 32.0 mmol), and 1.2 equiv of oxone (10.7 g, 34.9 mmol) in 2.0 mL of water and 100 mL of DMF was stirred at r.t. for 17 h. A precipitate formed and was filtered of to give 8.0 g (91% yield) of the title compound, which was used without further purification in the next step.

UPLC-MS (Waters Acquity acidic conditions): *t*_R_ = 1.11 min. MS (ESI+): *m*/*z* = 302.4 [M + H]^+^.

#### 5-Bromo-2-(4-ethylphenyl)-1-methyl-1*H*-benzimidazole and 6-Bromo-2-(4-ethylphenyl)-1-methyl-1*H*-benzimidazole (**8b-3** and **8c-3**)

A mixture of 5-bromo-2-(4-ethylphenyl)-1*H*-benzimidazole (2.00 g, 6.64 mmol), 1.1 equiv of iodomethane (455 μL, 7.3 mmol, and 1.2 equiv of Cs_2_CO_3_ (2.60 g, 7.97 mmol) in 30 mL of acetonitrile was stirred at r.t. until complete conversion. The reaction mixture was filtrated off and evaporated to dryness. The remaining crude material was purified by flash chromatography (silical gel, hexane, ethyl acetate gradient) to give 1.00 g (48% yield) of the title compound as mixture of two regioisomers which were not separated at this step.

UPLC-MS (Waters Acquity, acidic conditions): *t*_R_ = 1.20 and 1.21 min. MS (ESI+): *m*/*z* = 316.4 [M + H]^+^.

^1^H NMR (400 MHz, DMSO-*d*_6_) δ ppm 7.92 (d, *J* = 2.03 Hz, 1 H), 7.87 (d, *J* = 1.52 Hz, 1 H), 7.75–7.80 (m, 4 H), 7.59–7.63 (m, 2 H), 7.40–7.44 (m, 5 H), 7.37 (dd, *J* = 8.49, 1.90 Hz, 1 H), 3.87–3.90 (m, 1 H), 3.87 (2s, 2 × 3 H), 2.71 (d, *J* = 7.35 Hz, 2 × 2 H), 1.25 (t, *J* = 7.60 Hz, 2 × 3 H).

#### *tert*-Butyl [2-(4-ethylphenyl)-1-methyl-1*H*-benzimidazol-5-yl]carbamate and *tert*-Butyl [2-(4-ethylphenyl)-1-methyl-1*H*-benzimidazol-6-yl]carbamate (**8b–2** and **8c-2**)

A mixture of 5-bromo-2-(4-ethylphenyl)-1-methyl-1*H*-benzimidazole and 6-bromo-2-(4-ethylphenyl)-1-methyl-1*H*-benzimidazole (700 mg, 2.22 mmol), 1.2 equiv of *tert*-butyl carbamate (312 mg, 2.66 mmol), 0.03 equiv of bis(dibenzylidenaceton)palladium(0) (38 mg, 67 μmol), 0.1 equiv of *tert*-butyl-XPhos (94.3 mg, 222 μmol) and 2.0 equiv of sodium *tert*-butylate (427 mg, 4.44 mmol) in 110 mL of toluene was stirred at 60 °C for 3 days. The reaction mixture was evaporated to dryness, and the resulting crude material was purified by flash chromatography (silica gel, hexane/ethyl acetate gradient) to give 260 g (33% yield) of the title compound as mixture of regioisomers, which were not separated at this step.

UPLC-MS (Waters Acquity, acidic conditions): *t*_R_ = 1.01 and 1.04 min. MS (ESI+): *m*/*z* = 352.7 [M + H]^+^.

#### 2-(4-Ethylphenyl)-1-methyl-1*H*-benzimidazol-5-amine and 2-(4-Ethylphenyl)-1-methyl-1*H*-benzimidazol-6-amine (**8b-1** and **8c-1**)

A mixture *tert*-butyl [2-(4-ethylphenyl)-1-methyl-1*H*-benzimidazol-5-yl]carbamate and *tert*-butyl [2-(4-ethylphenyl)-1-methyl-1*H*-benzimidazol-6-yl]carbamate (260 mg, 740 μmol) and 1.8 mL of a HCl solution (4 N in dioxane, 10.0 equiv) was stirred at r.t. until complete conversion. The reaction mixture was evaporated to dryness to give 190 mg of the title compound as mixture of regioisomers, which were not separated at this step.

UPLC-MS (Waters Acquity, acidic conditions): *t*_R_ = 0.75 and 0.77 min. MS (ESI+): *m*/*z* = 252.2 [M + H]^+^.

#### 2-Chloro-*N*-[2-(4-ethylphenyl)-1-methyl-1*H*-benzimidazol-5-yl]-5-nitrobenzamide (**8c**)

A mixture of 2-chloro-5-nitrobenzoic acid (200 mg, 992 μmol) and 20 equiv of thionyl chloride (1.4 mL, 20 mmol) was stirred at 80 °C for 2 h. The mixture was evaporated to dryness and used immediately in the next step. A mixture of 2-(4-ethylphenyl)-1-methyl-1*H*-benzimidazol-5-amine and 2-(4-ethylphenyl)-1-methyl-1*H*-benzimidazol-6-amine (190 mg, 756 μmol), 1.3 equiv of 2-chloro-5-nitrobenzoyl chloride (216 mg, 983 μmol), and 3.0 equiv of TEA (320 μL, 2.3 mmol) in 3.5 mL of THF was stirred at r.t. until complete conversion. To the mixture were added DCM and water, and the phases wereseparated. The aqueous phase was extracted multiple times with DCM. The combined organic phases were evaporated to dryness, and the remaining crude material purified by flash chromatography to give 290 mg of a mixture of regioisomers, which were separated by chiral preparative SFC. Separation of regioisomers by SFC gave 90 mg (27% yield, 99% purity) of **8c**. Regiochemistry was unambiguously assigned using NOESY (Figure S7).

Analytical SFC: *t*_R_ = 4.55 min.

LC-MS (Agilent, acidic conditions): *t*_R_ = 0.90 min. MS (ESI+): *m*/*z* = 435.2 [M + H]^+^.

^1^H NMR (500 MHz, DMSO-*d*_6_) δ ppm 10.71 (s, 1 H), 8.50 (d, *J* = 2.86 Hz, 1 H), 8.33–8.38 (m, 1 H), 8.11 (d, *J* = 1.59 Hz, 1 H), 7.91 (d, *J* = 8.90 Hz, 1 H), 7.79 (d, *J* = 8.27 Hz, 2 H), 7.54–7.62 (m, 2 H), 7.43 (d, *J* = 8.27 Hz, 2 H), 3.89 (s, 3 H), 2.72 (q, *J* = 7.63 Hz, 2 H), 1.26 (t, *J* = 7.63 Hz, 3 H).

#### 2-Chloro-*N*-[2-(4-ethylphenyl)-1-methyl-1*H*-benzimidazol-6-yl]-5-nitrobenzamide (**8b**)

Separation of regioisomers by SFC gave 128 mg (39% yield, 98% purity) of **8b**. Regiochemistry was unambiguously assigned using NOESY (Figure S8).

Analytical SFC: *t*_R_ = 3.00 min.

LC-MS (Agilent, acidic conditions): *t*_R_ = 0.9 min. MS (ESI+): *m*/*z* = 435.2 [M + H]^+^.

^1^H NMR (500 MHz, DMSO-*d*_6_) δ ppm 10.83 (s, 1 H), 8.49 (d, *J* = 2.86 Hz, 1 H), 8.36 (dd, *J* = 8.90, 2.86 Hz, 1 H), 8.18 (d, *J* = 1.91 Hz, 1 H), 7.91 (d, *J* = 8.58 Hz, 1 H), 7.75–7.79 (m, 2 H), 7.66 (d, *J* = 8.58 Hz, 1 H), 7.42 (d, *J* = 8.27 Hz, 2 H), 7.37 (dd, *J* = 8.58, 1.91 Hz, 1 H), 3.86 (s, 3 H), 2.72 (q, *J* = 7.63 Hz, 2 H), 1.26 (t, *J* = 7.63 Hz, 3 H).

#### 2-Phenylimidazo[1,2-*a*]pyridin-6-amine (**8d-1**)

A mixture of 2-(2-chlorophenyl)-6-nitroimidazo[1,2-*a*]pyridine (5.38 g, 19.7 mmol) and Pd/charcoal in 120 mL of MeOH was stirred under a hydrogen atmosphere at r.t. for 6 h. The reaction mixture was filtrated over Celite and evaporated to dryness to give 2.0 g (49% yield) of the title compound, which was used without further purification in the next step.

UPLC-MS (Waters Acquity, acidic conditions): *t*_R_ = 0.59 min. MS (ESI+): *m*/*z* = 210.4 [M + H]^+^.

#### 2-Chloro-5-nitro-*N*-(2-phenylimidazo[1,2-*a*]pyridin-6-yl)benzamide (**8d**)

A mixture of 2-phenylimidazo[1,2-*a*]pyridin-6-amine (100 mg, 478 μmol), 1.3 equiv of 2-chloro-5-nitrobenzoic acid (125 mg, 621 μmol), 2.0 equiv of PyBrop (446 mg, 956 μmol), and 5.0 equiv of DIPEA (420 μL, 2.4 mmol) in 3.9 mL of DMF was stirred at r.t. until complete conversion. To the mixture were added water and DCM, and the phases were separated. The aqueous phase was extracted multiple times with DCM. The combined organic phases were evaporated to dryness. The remaining crude material was purified by preparative HPLC (acidic conditions) to give 20 mg 11% yield, 95% purity) of the title compound **8d**.

LC-MS (Agilent, acidic conditions): *t*_R_ = 0.78 min. MS (ESI+): *m*/*z* = 393.1 [M + H]^+^.

^1^H NMR (400 MHz, DMSO-*d*_6_) δ ppm 10.91 (s, 1 H), 9.34 (dd, *J* = 2.03, 1.01 Hz, 1 H), 8.54–8.58 (m, 2 H), 8.37 (dd, *J* = 8.87, 2.79 Hz, 1 H), 7.89–7.98 (m, 3 H), 7.63 (d, *J* = 9.63 Hz, 1 H), 7.41–7.48 (m, 2 H), 7.25–7.35 (m, 2 H).

#### 2-Chloro-*N*-[2-(4-methylphenyl)-2*H*-benzotriazol-5-yl]-5-nitrobenzamide (**8e**)

A mixture of 2-(4-methylphenyl)-2*H*-benzotriazol-5-amine (353 mg, 1.57 mmol), 1.5 equiv of 2-chloro-5-nitrobenzoic acid (476 mg, 2.36 mmol, 2.0 equiv of HATU (1.20 g, 3.15 mmol), and 5.0 equiv of DIPEA (1.4 mL, 7.9 mmol) in 6.9 mL of DMF was stirred at r.t. until complete conversion. The remaining crude material was purified by flash chromatography (silica gel, hexane/ethyl acetate gradient) to give 227 mg (36% yield, 100% purity) of the title compound **8e**.

UPLC-MS (acidic conditions): *t*_R_ = 1.40 min. MS (ESI+): *m*/*z* = 408.3 [M + H]^+^

^1^H NMR (400 MHz, DMSO-*d*_6_) δ ppm 11.06 (s, 1 H), 8.57 (d, *J* = 2.53 Hz, 2 H), 8.38 (dd, *J* = 8.87, 2.79 Hz, 1 H), 8.20 (d, *J* = 8.62 Hz, 2 H), 8.05 (d, *J* = 8.62 Hz, 1 H), 7.93 (d, *J* = 8.87 Hz, 1 H), 7.61 (dd, *J* = 9.25, 1.90 Hz, 1 H), 7.46 (d, *J* = 8.36 Hz, 2 H), 2.42 (s, 3 H).

#### 2-Chloro-*N*-[2-(4-ethylphenyl)-1,3-benzoxazol-5-yl]-5-nitrobenzene-1-sulfonamide (**8f**)

A mixture of 2-(4-ethylphenyl)-1,3-benzoxazol-5-amine (98.0 mg, 411 μmol), 1.0 equiv of 2-chloro-5-nitrobenzene-1-sulfonyl chloride (105 mg, 411 μmol), and 1.1 equiv of TEA (63 μL, 450 μmol) in 2.0 mL of DCM was stirred at r.t. for 17 h. To the mixture were added water and DCM, and the phases were separated. The aqueous phase was extracted multiple times with DCM. The combined organic phases were evaporated to dryness. The remaining crude material was purified by preparative HPLC (acidic conditions) to give 95.0 mg (50% yield, 100% purity) of the title compound **8f**.

UPLC-MS (Waters Acquity, acidic conditions): *t*_R_ = 1.45 min. MS (ESI+): *m*/*z* = 458.2 [M + H]^+^.

^1^H NMR (400 MHz, DMSO-*d*_6_) δ ppm 11.06 (s, 1 H), 8.65 (d, *J* = 2.53 Hz, 1 H), 8.40 (dd, *J* = 8.62, 2.79 Hz, 1 H), 8.01–8.08 (m, 2 H), 7.97 (d, *J* = 8.87 Hz, 1 H), 7.69 (d, *J* = 8.62 Hz, 1 H), 7.49 (d, *J* = 2.28 Hz, 1 H), 7.43 (d, *J* = 8.36 Hz, 2 H), 7.18 (dd, *J* = 8.74, 2.15 Hz, 1 H), 2.69 (q, *J* = 7.60 Hz, 2 H), 1.21 (t, *J* = 7.60 Hz, 3 H).

#### 4-(5,6-Dimethyl-1,3-benzoxazol-2-yl)aniline (**8g–1**)

Polyphoshoric acid (33 mL, 290 mmol) was heated 180 °C, and to the solution was added 1.0 g of 4-aminobenzoic acid (7.29 mmol) under vigorous stirring. The resulting mixture was stirred at 180 °C for 10 min, then 1.0 g of 2-amino-4,5-dimethylphenol (7.29 mmol) was added in portions. The resulting mixture was stirred at 180 °C for an additional 2 h. The mixture was added to ice water. To the mixture was added KOH, and the pH value was adjusted to pH 10. The aqueous phase was extracted multiple times with DCM. The combined organic phases were evaporated to dryness to give 1.1 g of the crude material of the title compound, which was used in the next step without further purification.

UPLC-MS (Waters Acquity, acidic conditions): *t*_R_ = 1.23 min. MS (ESI+): *m*/*z* = 239.4 [M + H]^+^.

#### 2-chloro-*N*-[4-(5,6-dimethyl-1,3-benzoxazol-2-yl)phenyl]-5-nitrobenzamide (**8g**)

A mixture of 4-(5,6-dimethyl-1,3-benzoxazol-2-yl)aniline (750 mg, 3.15 mmol), 1.3 equiv of 2-chloro-5-nitrobenzoic acid (825 mg, 4.09 mmol), 1.5 equiv of HATU (1.80 g, 4.72 mmol), and 5.0 equiv of TEA (2.2 mL, 16 mmol) in 14 mL of DMF was stirred at r.t. until complete conversion. To the mixture were added DCM and water, and the phases were separated. The aqueous phase was extracted multiple times with DCM. The combined organic phases were evaporated to dryness, and the remaining crude material purified by flash chromatography (silica gel, hexane/ethyl acetate gradient), followed by preparative HPLC (acidic conditions), to give 190 mg (14% yield, 100% purity) of the title compound **8g**.

UPLC-MS (Waters Acquity, acidic conditions): *t*_R_ = 1.43 min. MS (ESI+): *m*/*z* = 422.3 [M + H]^+^

^1^H NMR (400 MHz, DMSO-*d*_6_) δ ppm 11.07 (s, 1 H), 8.55 (d, *J* = 2.79 Hz, 1 H), 8.37 (dd, *J* = 8.74, 2.66 Hz, 1 H), 8.16–8.22 (m, 2 H), 7.90–7.96 (m, 3 H), 7.57 (d, *J* = 4.56 Hz, 2 H), 2.36 (s, 3 H), 2.34 (s, 3 H).

#### 2-Chloro-*N*-[3-(5-ethyl-1,3-benzoxazol-2-yl)phenyl]-5-nitrobenzamide (**8h**)

UPLC-MS (Waters Acquity, acidic conditions): *t*_R_ = 1.44 min.

MS (ESI+): *m*/*z* = 422.3 [M + H]^+^, 100% purity.

^1^H NMR (400 MHz, DMSO-*d*_6_) δ ppm 11.02 (s, 1 H), 8.74 (t, *J* = 1.77 Hz, 1 H), 8.56 (d, *J* = 2.53 Hz, 1 H), 8.37 (dd, *J* = 8.87, 2.79 Hz, 1 H), 7.95–8.00 (m, 1 H), 7.93 (d, *J* = 8.87 Hz, 1 H), 7.80–7.86 (m, 1 H), 7.72 (d, *J* = 8.36 Hz, 1 H), 7.59–7.68 (m, 2 H), 7.30 (dd, *J* = 8.49, 1.65 Hz, 1 H), 2.71–2.79 (m, 2 H), 1.25 (t, *J* = 7.60 Hz, 3 H).

#### 2-Chloro-*N*-[2-(4-ethylphenyl)-1,3-benzoxazol-5-yl]-5-(trifluoromethyl)benzamide (**9a**)

A mixture of 2-(4-ethylphenyl)-1,3-benzoxazol-5-amine (80.0 mg, 336 μmol), 1.3 equiv of 2-chloro-5-(trifluoromethyl)benzoic acid (98.0 mg, 436 μmol), 0.05 equiv of DMAP (2.05 mg, 16.8 μmol), 4.0 equiv of DIPEA (230 μL, 1.3 mmol), and 2.0 equiv of PyBrop (313 mg, 671 μmol) in 1.5 mL of DMF was stirred at r.t. until complete conversion. The reaction mixture was evaporated to dryness, and the remaining crude material purified by preparative HPLC (acidic conditions) to give 45.0 mg (30% yield, 100% purity) of the title compound **9a**.

UPLC-MS (Waters Acquity, acidic conditions): *t*_R_ = 1.48 min. MS (ESI+): *m*/*z* = 445.0 [M + H]^+^

^1^H NMR (400 MHz, DMSO-*d*_6_) δ ppm 10.77–10.88 (m, 1 H), 8.23 (d, *J* = 1.77 Hz, 1 H), 8.11–8.15 (m, 2 H), 8.09 (d, *J* = 2.28 Hz, 1 H), 7.89–7.94 (m, 1 H), 7.83–7.89 (m, 1 H), 7.78 (d, *J* = 8.62 Hz, 1 H), 7.65 (dd, *J* = 8.74, 2.15 Hz, 1 H), 7.47 (d, *J* = 8.62 Hz, 2 H), 2.72 (d, *J* = 7.60 Hz, 2 H), 1.24 (t, *J* = 7.60 Hz, 3 H).

#### *tert*-Butyl 2-chloro-5-(methylcarbamoyl)benzoate (**9b–2**)

A mixture of 3-(*tert*-butoxycarbonyl)-4-chlorobenzoic acid (1.23 g, 4.79 mmol), 2.0 equiv of methylamine (4.8 mL, 2.0 M, 9.6 mmol), 2.5 equiv of DIPEA (2.1 mL, 12 mmol), and 3.0 equiv of HATU (5.47 g, 14.4 mmol) in 289 mL of DMF was stirred at r.t. until complete conversion. To the mixture were added water and DCM, and the phases were separated. The aqueous phase was extracted multiple times with DCM. The combined organic phases were evaporated to dryness, and the remaining crude material purified by flash chromatography (silice gel, hexane/ethyl acetate gradient) to give 900 mg (70% yeld) of the title compound.

UPLC-MS (Waters Acquity, acidic conditions): *t*_R_ = 1.11 min. MS (ESI+): *m*/*z* = 270.1 [M + H]^+^.

#### 2-Chloro-5-(methylcarbamoyl)benzoic acid (**9b–1**)

A mixture of *tert*-butyl 2-chloro-5-(methylcarbamoyl)benzoate (900 mg, 3.34 mmol) and 8.3 mL of a HCl solution (4 N in dioxane, 10 equiv) was stirred at r.t. until complete conversion. To the mixture were added DCM and water, and the phases were separated. The aqueous phase was extracted multiple times with DCM. The aqueous phase was evaporated to dryness to give 700 mg (98% yield) of the title compound.

UPLC-MS (Waters Acquity, acidic conditions): *t*_R_ = 0.59 min. MS (ESI+): *m*/*z* = 214.0 [M + H]^+^.

#### 4-Chloro-N^3^-[2-(4-ethylphenyl)-1,3-benzoxazol-5-yl]-*N*^1^-methylbenzene-1,3-dicarboxamide (**9b**)

A mixture of 2-chloro-5-(methylcarbamoyl)benzoic acid (300 mg, 1.40 mmol) and 20 equiv of thionyl chloride (2.0 mL, 28 mmol) was stirred at 80 °C for 2.5 h. The mixture was evaporated to dryness and used immediately in the next step. A mixture of 2-(4-ethylphenyl)-1,3-benzoxazol-5-amine (156 mg, 657 μmol), 1.05 equiv of 2-chloro-5-(methylcarbamoyl)benzoyl chloride (160 mg, 689 μmol), and 3.0 equiv of TEA (270 μL, 2.0 mmol) in 4.0 mL of THF was stirred at r.t. for 12 h and evaporated to dryness. The crude material was taken up in DCM, and the mixture was stirred at r.t., filtered, and evaporated to dryness again to give 150 mg (52% yield, 98% purity) of the title compound **9b**.

UPLC-MS (Agilent, acidic conditions): *t*_R_ = 1.29 min. MS (ESI+): *m*/*z* = 434.1 [M + H]^+^.

^1^H NMR (400 MHz, DMSO-*d*_6_) δ ppm 10.87 (s, 1 H), 8.82 (br d, *J* = 4.82 Hz, 1 H), 8.27 (d, *J* = 1.77 Hz, 1 H), 8.09–8.16 (m, 3 H), 7.99 (dd, *J* = 8.36, 2.28 Hz, 1 H), 7.75–7.80 (m, 1 H), 7.66–7.74 (m, 2 H), 7.47 (d, *J* = 8.36 Hz, 2 H), 2.79 (d, *J* = 4.56 Hz, 3 H), 2.72 (d, *J* = 7.60 Hz, 2 H), 1.23 (t, *J* = 7.60 Hz, 3 H).

#### 2-Chloro-*N*-[2-(4-ethylphenyl)-1,3-benzoxazol-5-yl]pyridine-3-carboxamide (**9c**)

A mixture of 2-(4-ethylphenyl)-1,3-benzoxazol-5-amine (100 mg, 420 μmol), 1.0 equiv of 2-chloropyridine-3-carboxylic acid (66.1 mg, 420 μmol), 5.0 equiv of TEA (290 μL, 2.1 mmol) ,and 2.0 equiv of HATU (319 mg, 839 μmol) in 6.0 mL of THF was stirred at r.t. until complete conversion and then evaporated to dryness. To the mixture were added water and DCM, and the phases were separated. The organic phase was evaporated to dryness, and the remaining crude material purified by preparative HPLC (acidic conditions) to give 14 mg (9% yield, 100% purity) of the title compound **9c**.

UPLC-MS (Waters Acquity, acidic conditions): *t*_R_ = 1.27 min. MS (ESI+): *m*/*z* = 378.0 [M + H]^+^

^1^H NMR (400 MHz, DMSO-*d*_6_) δ ppm 10.78–10.91 (m, 1 H), 8.56 (dd, *J* = 4.82, 1.77 Hz, 1 H), 8.22 (d, *J* = 2.03 Hz, 1 H), 8.10–8.16 (m, 3 H), 7.78 (d, *J* = 8.87 Hz, 1 H), 7.65 (dd, *J* = 8.87, 2.03 Hz, 1 H), 7.59 (dd, *J* = 7.60, 4.82 Hz, 1 H), 7.47 (d, *J* = 8.62 Hz, 2 H), 2.68–2.76 (m, 2 H), 1.24 (t, *J* = 7.60 Hz, 3 H).

#### 2-Chloro-5-cyano-*N*-[2-(4-ethylphenyl)-1,3-benzoxazol-5-yl]benzamide (**9d**)

A mixture of 22-(4-ethylphenyl)-1,3-benzoxazol-5-amine (100 mg, 420 μmol), 1.5 equiv of 2-chloro-5-cyanobenzoic acid (114 mg, 629 μmol, 5.0 equiv of DIPEA (370 μL, 2.1 mmol), and 1.5 equiv of HATU (239 mg, 629 μmol) in 2.4 mL of DMF was stirred at r.t. until complete conversion and evaporated to dryness. The remaining crude material purified by preparative HPLC (acidic conditions) to give 68 mg (38% yield, 97% purity) of the title compound **9d**.

UPLC-MS (Waters Acquity, acidic conditions): *t*_R_ = 1.36 min. MS (ESI+): *m*/*z* = 402.0 [M + H]^+^.

^1^H NMR (400 MHz, methanol-*d*_4_) δ ppm 8.21 (d, *J* = 1.52 Hz, 1 H), 8.13–8.18 (m, 2 H), 8.03 (d, *J* = 2.03 Hz, 1 H), 7.85 (dd, *J* = 8.36, 2.03 Hz, 1 H), 7.74 (d, *J* = 8.36 Hz, 1 H), 7.65–7.69 (m, 1 H), 7.59–7.64 (m, 1 H), 7.43 (d, *J* = 8.62 Hz, 2 H), 2.76 (q, *J* = 7.60 Hz, 2 H), 1.29 (t, *J* = 7.60 Hz, 3 H).

#### *N*-[2-(4-Ethylphenyl)-1,3-benzoxazol-5-yl]-3-nitrobenzamide (**9e**)

A mixture of 2-(4-ethylphenyl)-1,3-benzoxazol-5-amine (90.0 mg, 378 μmol), 1.3 equiv of 3-nitrobenzoic acid (82.1 mg, 491 μmol), 2 equiv of PyBrop (352 mg, 755 μmol), and 5.0 equiv of DIPEA (330 μL, 1.9 mmol) in 3.1 mL of DMF was stirred at r.t. until complete conversion. The mixture was extracted multiple times with DCM. The combined organic phases were washed with water and evaporated to dryness. The remaining crude material purified by preparative HPLC (acidic conditions) to give 20 mg (14% yield, 99% purity) of the title compound **9e**.

UPLC-MS (Waters Acquity, basic conditions): *t*_R_ = 1.36 min. MS (ESI+): *m*/*z* = 388.0 [M + H]^+^.

^1^H NMR (400 MHz, DMSO-*d*_6_) δ ppm 10.77 (s, 1 H), 8.83 (t, *J* = 2.03 Hz, 1 H), 8.42–8.49 (m, 2 H), 8.27 (d, *J* = 1.77 Hz, 1 H), 8.09–8.16 (m, 2 H), 7.87 (t, *J* = 7.98 Hz, 1 H), 7.72–7.82 (m, 2 H), 7.47 (d, *J* = 8.62 Hz, 2 H), 2.68–2.77 (m, 2 H), 1.24 (t, *J* = 7.60 Hz, 3 H).

#### 2-Chloro-*N*-[2-(3-methylphenyl)-1,3-benzoxazol-5-yl]-3-nitrobenzamide (**9f**)

A mixture of 2-(3-methylphenyl)-1,3-benzoxazol-5-amine (99.0 mg, 441 μmol), 1.2 equiv of 2-chloro-3-nitrobenzoic acid (107 mg, 530 μmol), 5.0 equiv of TEA (310 μL, 2.2 mmol), and 1.2 equiv of HATU (201 mg, 530 μmol) in 2.0 mL of DMF was stirred at r.t. until complete conversion and then evaporated to dryness. The remaining crude material purified by flash chromatography (silica gel, hexane/ethyl acetate gradient) to give 83 mg (46% yield, 100% purity) of the title compound **9f**.

UPLC-MS (basic conditions): *t*_R_ = 1.34 min. MS (ESI+): *m*/*z* = 408.2 [M + H]^+^.

^1^H NMR (400 MHz, DMSO-*d*_6_) δ ppm 10.91 (s, 1 H), 8.24 (d, *J* = 1.77 Hz, 1 H), 8.18 (dd, *J* = 8.11, 1.52 Hz, 1 H), 7.95–8.07 (m, 3 H), 7.72–7.83 (m, 2 H), 7.66 (dd, *J* = 8.74, 2.15 Hz, 1 H), 7.44–7.55 (m, 2 H), 2.44 (s, 3 H).

#### *N*-[2-(4-Ethylphenyl)-1,3-benzoxazol-5-yl]-5-nitrothiophene-2-carboxamide (**9g**)

A mixture of 2-(4-ethylphenyl)-1,3-benzoxazol-5-amine (90.0 mg, 378 μmol), 1.3 equiv of 5-nitrothiophene-2-carboxylic acid (85.0 mg, 491 μmol), 2 equiv of PyBrop (352 mg, 755 μmol), and 5.0 equiv of DIPEA (330 μL, 1.9 mmol) in 3.1 mL of DMF was stirred at r.t. until complete conversion. To the mixture were added DCM and water, and the phases were separated. The aqueous phase was extracted multiple times with DCM. The combined organic phases were evaporated to dryness. Methanol was added to the remaining crude material, and the solid material was filtered off to give 15 mg (10% yield, 100% purity) of the title compound **9g**.

UPLC-MS (Agilent, acidic conditions): *t*_R_ = 1.42 min. MS (ESI+): *m*/*z* = 394.1 [M + H]^+^.

^1^H NMR (400 MHz, DMSO-*d*_6_) δ ppm 10.81 (s, 1 H), 8.24 (d, *J* = 4.31 Hz, 1 H), 8.20 (d, *J* = 2.03 Hz, 1 H), 8.07–8.15 (m, 3 H), 7.80 (d, *J* = 8.87 Hz, 1 H), 7.69 (dd, *J* = 8.87, 2.03 Hz, 1 H), 7.47 (d, *J* = 8.36 Hz, 2 H), 2.72 (q, *J* = 7.60 Hz, 2 H), 1.24 (t, *J* = 7.60 Hz, 3 H.

#### *N*-[2-(4-Ethylphenyl)-1,3-benzoxazol-5-yl]prop-2-enamide (**9h**)

A mixture of 2-(4-ethylphenyl)-1,3-benzoxazol-5-amine (99.0 mg, 415 μmol), 1.3 equiv of prop-2-enoic acid (38.9 mg, 540 μmol), 4.0 equiv of DIPEA (290 μL, 1.7 mmol), and 1.2 equiv of HATU (190 mg, 499 μmol) in 1.8 mL of DMF was stirred at r.t. until complete conversion. To the mixture were added DCM and water, and the phases were separated. The aqueous phase was extracted multiple times with DCM. The combined organic phases were evaporated to dryness. The remaining crude material purified by preparative HPLC (acidic conditions) to give 26 mg (20% yield, 97% purity) of the title compound **9h**.

UPLC-MS (Waters Acquity, acidic conditions): *t*_R_ = 1.21 min. MS (ESI+): *m*/*z* = 293.0 [M + H]^+^.

^1^H NMR (400 MHz, DMSO-*d*_6_) δ ppm 10.34 (s, 1 H), 8.23 (d, *J* = 2.03 Hz, 1 H), 8.09–8.14 (m, 2 H), 7.73 (d, *J* = 8.62 Hz, 1 H), 7.58 (dd, *J* = 8.87, 2.03 Hz, 1 H), 7.46 (d, *J* = 8.36 Hz, 2 H), 6.41–6.52 (m, 1 H), 6.26–6.34 (m, 1 H), 5.79 (dd, *J* = 9.89, 2.03 Hz, 1 H), 2.72 (q, *J* = 7.60 Hz, 2 H), 1.21–1.26 (m, 3 H).

#### *N*-[2-(4-Ethylphenyl)-1,3-benzoxazol-5-yl]-2-fluoro-5-nitrobenzamide (**9i**)

A mixture of 2-(4-ethylphenyl)-1,3-benzoxazol-5-amine (80.0 mg, 336 μmol), 1.3 equiv of 2-fluoro-5-nitrobenzoic acid (80.8 mg, 436 μmol), 2 equiv of PyBrop (313 mg, 671 μmol), 0.05 equiv of DMAP (2.05 mg, 16.8 μmol), and 4.0 equiv of DIPEA (230 μL, 1.3 mmol) in 1.5 mL of DMF was stirred at r.t. until complete conversion. To the mixture were added DCM and water, and the phases were separated. The aqueous phase was extracted multiple times with DCM. The combined organic phases were evaporated to dryness. The remaining crude material was purified by preparative HPLC (acidic conditions) to give 30 mg (22% yield, 100% purity) of the title compound **9i**.

UPLC-MS (Waters Acquity, acidic conditions): *t*_R_ = 1.37 min. MS (ESI+): *m*/*z* = 406.0 [M + H]^+^.

^1^H NMR (400 MHz, DMSO-*d*_6_) δ ppm 10.70–10.95 (m, 1 H), 8.59 (dd, *J* = 5.96, 2.91 Hz, 1 H), 8.45–8.52 (m, 1 H), 8.23 (d, *J* = 2.03 Hz, 1 H), 8.10–8.16 (m, 2 H), 7.79 (d, *J* = 8.62 Hz, 1 H), 7.63–7.75 (m, 2 H), 7.47 (d, *J* = 8.36 Hz, 2 H), 2.68–2.76 (m, 2 H), 1.24 (t, *J* = 7.60 Hz, 3 H).

#### 2-Bromo-*N*-[2-(4-ethylphenyl)-1,3-benzoxazol-5-yl]-5-nitrobenzamide (**9j**, **BAY-4931**)

A mixture of 2-(4-ethylphenyl)-1,3-benzoxazol-5-amine (200 mg, 839 μmol), 1.05 equiv of 2-bromo-5-nitrobenzoic acid (217 mg, 881 μmol), 2 equiv of PyBrop (783 mg, 1.68 mmol), 0.05 equiv of DMAP (5.13 mg, 42.0 μmol), and 4.0 equiv of DIPEA (580 μL, 3.4 mmol) in 3.7 mL of DMF was stirred at r.t. until complete conversion. To the mixture were added DCM and water, and the phases were separated. The aqueous phase was extracted multiple times with ethyl acetate. The combined organic phases were evaporated to dryness. The remaining crude material was purified by preparative HPLC (acidic conditions) to give 65 mg (16% yield, 100% purity) of the title compound **9j**.

UPLC-MS (Waters Acquity, acidic conditions): *t*_R_ = 1.44 min. MS (ESI+): *m*/*z* = 466.1 [M + H]^+^. Refer to Figure S6 for the HPLC trace of **9j** (**BAY-0069**). MS (ESI+): *m*/*z* = 468.2 [M + H]^+^.

^1^H NMR (400 MHz, DMSO-*d*_6_) δ ppm 10.88 (s, 1 H), 8.47 (d, J = 2.53 Hz, 1 H), 8.26 (dd, J = 8.87, 2.79 Hz, 1 H), 8.22 (d, J = 2.03 Hz, 1 H), 8.11–8.16 (m, 2 H), 8.07 (d, J = 8.87 Hz, 1 H), 7.79 (d, J = 8.87 Hz, 1 H), 7.65 (dd, J = 8.74, 2.15 Hz, 1 H), 7.47 (d, J = 8.62 Hz, 2 H), 2.72 (q, J = 7.60 Hz, 2 H), 1.24 (t, J = 7.60 Hz, 3 H). LC-MS (acidic conditions): *t*_R_ = 1.43 min.

#### *N*-[2-(4-Ethylphenyl)-1,3-benzoxazol-5-yl]-2-iodo-5-nitrobenzamide (**9k**)

A mixture of 2-(4-ethylphenyl)-1,3-benzoxazol-5-amine (70.0 mg, 294 μmol), 1.2 equiv of 2-iodo-5-nitrobenzoic acid (103 mg, 353 μmol), 2.4 equiv of DIPEA (120 μL, 710 μmol), and 1.2 equiv of HATU (134 mg, 353 μmol) in 2.7 mL of DMF was stirred at r.t. until complete conversion. The reaction mixture was evaporated to dryness, and the remaining crude material was purified by preparative HPLC (acidic conditions) to give 37 mg (24% yield, 100% purity) of the title compound **9k**.

UPLC-MS (Agilent, acidic conditions): *t*_R_ = 1.42 min. MS (ESI+): *m*/*z* = 514.0 [M + H]^+^.

^1^H NMR (400 MHz, DMSO-*d*_6_) δ ppm 10.81 (s, 1 H), 8.34 (d, *J* = 2.79 Hz, 1 H), 8.28 (d, *J* = 8.62 Hz, 1 H), 8.21 (d, *J* = 1.77 Hz, 1 H), 8.13 (d, *J* = 8.11 Hz, 2 H), 8.04 (dd, *J* = 8.62, 2.79 Hz, 1 H), 7.80 (d, *J* = 8.87 Hz, 1 H), 7.66 (dd, *J* = 8.87, 2.03 Hz, 1 H), 7.48 (d, *J* = 8.62 Hz, 2 H), 2.69–2.77 (m, 2 H), 1.24 (t, *J* = 7.60 Hz, 3 H).

### Solubility from DMSO in Buffer pH 6.5

Aqueous solubility was determined by an orientating high-throughput screening method. Solubility was determined in PBS buffer pH 6.5 containing 1% DMSO. Test compounds were applied as 1 mM DMSO solutions. After the addition of pH 6.5 PBS buffer, solutions were shaken for 24 h at room temperature. Undissolved material was removed by filtration using a MultiScreen Solubility Filter Plate (Millipore) according to the manufacturer’s protocol. The compound dissolved in the filtrate was quantified by HPLC-UV. The response was fitted to a one-point standard curve prepared in DMSO.

### Compound Stability

Stability versus nucleophilic thiols (cysteine, glutathione) at 37 °C was determined essentially as described.^18^ Stability versus nucleophiles was determined by HPLC-UV. To 1 mL of acetonitrile was added 5 μL of a 10 mM solution of the compound in DMSO. The reaction occurred in the presence of a 100-fold excess of thiol. Cysteine and glutathione, respectively, were dissolved in pH 7.4 buffer to give a 500 μM reaction solution. To the 1 mL thiol reaction solution was added 100 μL of the drug solution. Injections were made immediately after mixing for the time-zero injection and then again after 1, 2, 4, and 24 h. Compounds were incubated at 37 °C. The degradation rate (recovery in %) was calculated by relating peak areas after 1, 2, 4, and 24 h to the time-zero injection.

### Mass Spectrometry Covalent Binding Assay

To determine relative covalent binding (%]) to PPARγ via denaturing intact mass analysis, 5 μM human recombinant PPARγ was incubated with 5 μM compound (n = 2) at room temperature. Each sample contained 1% DMSO, including the PPARγ control without compound. Reactions were stopped after 1 h via the addition of 2 μL of 4% TFA to a 10 μL reaction volume. Samples were measured on a Waters I Class UPLC coupled to a Waters SYNAPT G2-S quadrupole-TOF electrospray instrument operated in the ESI+ mode. The Waters I Class UPLC instrument was equipped with a Waters Mass Prep C4 column, 2.1 × 5 mm. The column temperature was set to 65 °C, and the flow rate was 100 μL/min. Samples were measured with a gradient from 20% buffer B to 80% buffer in 1.9 min, with a total run time of 6 min. Buffer A contained water + 0.1% formic acid, and buffer B contained MeCN + 0.1% formic acid. The MS settings were as follows: *m*/*z* range, 150–2200; scan time, 1 s; acquisition mode, resolution; and acquisition time 5 min.

### PPARγ Biochemical Competitive Binding Assay

The assay was performed as described previously.^3^ Ultrahigh-throughput screen was performed using LanthaScreen TR-FRET PPARγ competitive binding assay. Essentially, the epitope-tagged PPARγ ligand binding domain (PPARγ-LBD) was tested in a biochemical TR-FRET interaction assay with the nonselective PPARγ ligand, fluormone, using LanthaScreen assay technology (LanthaScreen TR-FRET PPARγ competitive binding assay, ThermoFisher). The assay was performed according to the manufacturers protocol with an adaptation for miniaturization to a 1536-well format.

### PPARγ:MED1 TR-FRET Assay

A biochemical interaction assay measuring the ligand-dependent changes in interactions between the PPARγ-LBD and a fluorescent peptide from the coactivator *MED1* (TRAP220/DRIP-2) was performed to evaluate ligand-dependent PPARγ:coactivator interactions This assay was performed according to the manufacturer’s protocol (LanthaScreen TR-FRET PPARγ coactivator assay, ThermoFisher). Two hours after the dose–response compound treatment, plates were read using an EnVision Multimode plate reader (PerkinElmer) equipped with the LanthaScreen Advanced Dual Label filter set (PerkinElmer).

### PPARγ:NCOR1 TR-FRET and PPARγ:NCOR2 CRR TR-FRET Assays

To evaluate inverse-agonist activity in a biochemical activity, we modified the PPARγ:MED1 TR-FRET assay to replace the TRAP220/DRIP-2 coactivator peptide included in the kit was with fluorescent-labeled corepressor peptides from NCOR1 (NCoR ID2) or NCOR2 (Smrt ID2) (ThermoFisher) as described previously.^3^ Two hours after the dose–response compound treatment, with each point in duplicate, plates were read using a microplate reader in TR-FRET mode according to the manufacturer’s instructions. EC_50_ and *E*_max_ values for PPARγ:NCOR2 are the means from at least two experiments.

### PPARA Biochemical Competitive Binding Assay

The LanthaScreen TR-FRET PPARα competitive binding assay kit (ThermoFisher) was performed according to the manufacturer’s protocol to evaluate binding to PPARα. Twenty-four hours after the dose–response compound treatment, plates were read using a microplate reader in the TR-FRET mode according to the manufacturer’s instructions.

### GAL4-NHR-LBD Chimera Reporter Gene Assays

CHO cells expressing the Firefly Luciferase gene under the control of a GAL4 promoter (pFR-Luc, Stratagene) were stably transfected with fusion proteins of the GAL4 DNA binding domain (pFC-dbd, Stratagene) and the indicated nuclear hormone receptor ligand binding domain (NHR-LBD). Then, 2500 cells were plated per well in 384-well plates in OptiMEM with 2.5% fetal bovine serum and allowed to adhere prior to the addition of compounds in ten concentrations, each in quadruplicate. Six hours after compound treatment, reporter activity was quantified by lysis and the addition of the substrate Luciferin. Luminescence activity was detected using a luminescence plate reader. Every measurement was performed in at least two independent experiments. Receptors include human PPARγ, PPARA, and PPARD in addition to mouse versions (Pparγ).

### Cell Lines

UM-UC-9 cells were purchased from the European Collection of Authenticated Cell Cultures (EcACC). PaCaDD-188 and PaCaDD-161 were purchased from DSMZ (Germany). All other cell lines were obtained from the Cancer Cell Line Encyclopedia^19^ (Broad Institute, Cambridge, MA), which obtained them from the original source and performed cell line authentication. Cell lines were tested for mycoplasma contamination.

### RT112-FABP4-NLucP Reporter Gene Assay

The PPARγ reporter assay was performed as described.^3^ Essentially, the NanoLuciferase gene was engineered into the 3′-UTR of the canonical PPARγ target gene *FABP4* in RT112/84 cells using Cas9-guided homology-dependent repair. A single-cell clone was selected and expanded. From 5000 to 10 000 cells were plated per well in 384-well plates in culture media containing 10% fetal bovine serum and allowed to adhere prior to the addition of compounds in a dose–response manner. Twenty hours after compound treatment, reporter activity was quantified using the Nano-Glo Luciferase assay system (Promega, Madison, Wisconsin), and luminescence activity was detected using a luminescence plate reader. IC_50_ and *E*_max_ values were calculated from four replicates per concentration, and reported values are the means of at least two independent experiments.

### Colony Formation Assay and Crystal Violet Staining

Cells were plated in triplicate in 12-well plates in 1 mL of media per well and allowed to adhere prior to treatment with the vehicle or compound. All cell lines were grown in MEMα containing 10% FBS with the exception of PaCaDD-188 and PaCaDD-161, which were grown in Dresden media as previously described.^19^ Plating density was determined prior in order to achieve approximately 80% confluency in 7–14 days with vehicle treatment. For the experimental plates, cells were treated with the DMSO vehicle, the neutral antagonist GW9662 (100 nM), T0070907 (100 nM), or BAY 4931 (100 nM) using the HP-D300e Digital Dispenser (Tecan). Every three or four days, media and compounds were replenished. When vehicle-treated cells reached approximately 80% confluency, cells were stained with crystal violet as previously described.^20^ Briefly, media were removed, and cells were washed with PBS. Cells were fixed using 4% formaldehyde in PBS for 30 min at room temperature. The formaldehyde solution was removed, and the cells were stained with crystal violet for another 30 min, after which the stain was removed and washed out. The plates were allowed to dry inverted overnight and imaged the following day using an Epson Perfection 600 scanner.

### UM-UC-9 Proliferation

UM-UC-9 were grown in MEM-α media (Gibco) containing 10% heat-inactivated fetal bovine serum (Sigma). The cells were stably transduced with a lentiviral expression vector encoding the TagGFP-Histone-2B cDNA (pTagGFP2-H2B, Evrogen). Cells were plated at 500 cells per well in a 384-well view-plate and allowed to attach at 37 °C for 2 h. Plates were dosed with compounds at the indicated concentrations in duplicate. Seven days after addition of the compound, nuclei were counted with the use of an IncuCyte S3 Live Cell Imager. Cell counts were normalized to the vehicle control, and data are reported as IC_50_, *E*_max_ and the percent of the vehicle control. All compounds reported were also evaluated in an independent experiment read out at day 5, with similar results.

### FABP4 RTqPCR

High-throughput RTqPCR was performed using LightCycler 1536 (Roche) instrument according to the manufacturers protocol. RT112/84 cells were incubated the compounds for 24 h, and *FABP4* (primer sequence TAAACTGGTGGTGGAATGCG, GCGAACTTCAGTCCAGGTCA, TCATGAAAGGCGTCACTTCCACGAGA) expression was measured to monitor the activity of *PPARG*, *RPL30* (primer sequence GTCCCGCTCCTAAGGCAG, GTTGATCGACTCCAGCGACT, AGATGGTGGCCGCAAAGAAGACGAA) was used as a housekeeper gene. The qPCR was performed as a one-step measurement in cell lysates using the LightCycler RNA Virus Master PCR kit (Roche) according to the manufacturer’s protocol in a total volume of 1 μL. The data were first normalized to the housekeeping gene and are shown as the relative expression compared to the vehicle control.

### *In Vitro* Metabolic Stability Assay in Rat Hepatocytes

To generate the primary hepatocyte suspension, hepatocytes from male Wistar rats were isolated via a two-step perfusion method. After perfusion, the liver was carefully removed from the rat, the liver capsule was opened, and the hepatocytes were gently shaken out into a Petri dish with ice-cold Williams’ medium E (WME). The resulting cell suspension was filtered through sterile gauze into 50 mL Falcon tubes and centrifuged at 50 × *g* for 3 min at room temperature. The cell pellet was resuspended in 30 mL of WME and centrifuged through a Percoll gradient two times at 100 × *g*. The hepatocytes were washed again with WME and resuspended in a medium containing 5% FCS. Cell viability was determined by trypan blue exclusion. For the metabolic stability assays, liver cells were distributed in WME containing 5% FCS into glass vials at a density of 1.0 × 106 vital cells/mL.

The test compound was added to a final concentration of 1 μM. The organic solvent in the incubations was limited to ≤0.01% DMSO and ≤1% acetonitrile. During incubation, the hepatocyte suspensions were continuously shaken at 580 rpm, and aliquots were taken at 2, 8, 16, 30, 45, and 90 min. To the aliquots were immediately added an equal volume of cold acetonitrile. Samples were frozen at −20 °C overnight and subsequently centrifuged at 3000 rpm for 15 min. The supernatant was analyzed with an Agilent 1200 HPLC system with MS/MS detection. The half-life of a test compound was determined from the concentration–time plot. From the half-life, the intrinsic and the *in vitro* predicted blood clearances were calculated in addition to the hepatic extraction ratio EH = (CLb/LBF) × 100% according to the “well-stirred” liver model.^21^ In combination with the standardized liver blood flow (LBF) of 4.2 L/h/kg, a specific liver weight of 32 g/kg body weight, and amount of liver cells *in vivo* (1.1 × 108 cells/g liver) and *in vitro* (1.0 × 106/mL), the *in vitro* blood clearance (CLb, *in vitro*) and the maximal bioavailability (*F*_max_ (%) = 1 – EH × 100%) were calculated.

### *In Vitro* Metabolic Stability in Liver Microsomes

The *in vitro* metabolic stability of test compounds was determined by incubation at 1 μM in a suspension of liver microsomes in 100 mM pH 7.4 phosphate buffer (NaH2PO4·H2O + Na2HPO4·2H2O) at a protein concentration of 0.5 mg/mL at 37 °C. The microsomes were activated by adding a cofactor mix containing 8 mM glucose-6-phosphate, 0.5 mM NADP, and 1 IU/mL glucose-6-phosphate dehydrogenase in pH 7.4 phosphate buffer. The metabolic assay was started shortly afterward by adding the test compound to the incubation mixture at a final volume of 1 mL. During incubation, the microsomal suspensions were continuously shaken at 580 rpm, and aliquots were taken at 2, 8, 16, 30, 45, and 60 min. Further handling and analysis were performed as per the hepatocyte method described above with the human specific scaling factors of a liver blood flow of 1.32 L/h/kg and a specific liver weight of 21 g/kg body weight.

### Estimation of Plasma Protein Binding by Flux Dialysis

The binding of test compounds to plasma proteins was measured using a modified standard equilibrium dialysis procedure in a 96-well format at 37 °C and 5% CO_2_ atm using HT-Dialysis equipment made of Teflon. The Flux dialysis method is based on the principle that the initial flux rate (R_slope) of a compound is proportional to the product of compound initial concentration (fu) and the unbound dialysis membrane permeability (P_mem). Therefore, fu can be determined from R_slope when the membrane P_mem is known. Common equipment and an assay-specific P_mem value of of 75.2 × 10–6 cm/s, which was established previously, was used for calculation.^22^

In brief, a semipermeable membrane (regenerated cellulose, MWCO 12–14K) separates the plasma donor and plasma receiver sides, which are each filled with 150 μL of plasma. The test compound is added to the donor side at 1 μM and binds to plasma proteins. The unbound fraction of the test compound passes the membrane and distributes on both sides until equilibrium is reached. The flux rate as the rate of the appearance of the compound in the receiver side is approximated from the time course of the quotient of receiver and the donor concentration (*R*) by nonlinear regression including data from an entire time course. For this purpose, samples were taken at different time points (up to 96 h) from the donor and receiver side, and the relative compound concentration (peak area ratios analyte/IS) was measured by LC-MSMS analytics. Prior to this, both sides were matrix matched (diluted with buffer and plasma to achieve the same matrix of 10% plasma) and subsequently precipitated with a fourfold volume of methanol containing an appropriate internal standard (IS).

### Caco-2 Permeability

Caco-2 cells (DSMZ) were seeded at a density of 2.5 × 105 cells/well on 24-well insert plates (0.4 μm pore size, 0.3 cm^2^ (Costar)) and grown for 13–15 d in a DMEM medium supplemented with 10% FCS, 1% GlutaMAX (100× , Gibco), 100 U/mL penicillin, 100 μg/mL streptomycin (Gibco), and 1% nonessential amino acids (100 × ). Cells were maintained at 37 °C in a humidified 5% CO2 atm. Medium was changed every 2–3 d.

The bidirectional transport assay for the evaluation of Caco-2 permeability was undertaken in 24-well insert plates using a robotic system (Tecan). Before the assay was run, the culture medium was replaced with the transport medium (FCS-free HEPES carbonate transport buffer, pH 7.2). To assess the monolayer integrity, the transepithelial electrical resistance (TEER) was measured. Only monolayers with a TEER of at least 400 Ω·cm^2^ were used. Test compounds were predissolved in DMSO, and the solutions were added to either the apical or basolateral compartment at a final concentration of 2 μM. Evaluation was done in triplicate. Before and after incubation for 2 h at 37 °C, samples were taken from both compartments and analyzed by LC-MS/MS after precipitation with MeOH. The apparent permeability coefficient (*P*_app_) was calculated for both the apical to basolateral (A → B) and the basolateral to apical (B → A) directions using following equation: *P*_app_ = (*V*_r_/*P*_0_)(1/*S*)(*P*_2_/*t*), where *V*_r_ is the volume of the medium in the receiver chamber, *P*_0_ is the measured peak area of the test compound in the donor chamber at *t* = 0, *S* is the surface area of the monolayer, *P*_2_ is the measured peak area of the test compound in the acceptor chamber after incubation for 2 h, and *t* is the incubation time. The basolateral (B) to apical (A) efflux ration was calculated by dividing *P*_app_(B–A) by *P*_app_(A–B).

### Metabolite Identification in Rat Hepatocytes

The test compound was incubated at 37 °C in a hepatocyte suspension containing 1 × 10^6^ cells/mL in a round-shaking water bath at 116 rpm for 1, 2, and 4 h. To the suspension was added 5 μM test compound from a 0.1 mM stock solution dissolved in acetonitrile. Enzymatic activities of all hepatocyte preparations were measured using a variety of standard substrates. All hepatocytes exhibited good activities. The incubations were terminated by the addition of acetonitrile (approximately 30% (v/v)) and stored at 18 °C until analysis. Prior to analysis, the samples were thawed and centrifuged at 12 000 rpm for 10 min. Aliquots of 10 μL of the supernatant were used to control the recovery of the radioactivity by liquid scintillation counting. Aliquots of the supernatants were transferred into HPLC vials and analyzed by HPLC and online MS detection using the Orbitrap Fusion Lumos mass spectrometer and parallel split to UV detection. Exact mass and mass changes in comparison with the parent drug in combination with the fragment pattern from MS/MS were used to propose the structures of metabolites or confirm structures by comparison to those of reference compounds.

### CYP Inhibition

The inhibitory potency of test compounds toward CYP450-dependent metabolic pathways was determined in pooled human liver microsomes (Xenotech, USA) by applying individual CYP isoform-selective standard probes (CYP1A2, phenacetin; CYP2C8, amodiaquine; CYP2C9, diclofenac; CYP2D6, dextromethorphan; and CYP3A4, midazolam). Reference inhibitors were included as positive controls. Incubation conditions (protein and substrate concentrations and incubation time) were optimized regarding the linearity of metabolite formation. Assays were processed in 96-well microtiter plates at 37 °C using a Genesis Workstation (Tecan, Crailsheim, Germany). After protein precipitation, metabolite formation was quantified by LC-MS/MS analysis, followed by inhibition evaluation and IC_50_ calculation.

### PXR NOEL Assay

A HepG2 cell lines stably cotransfected with a vector for human PXR and a Luciferase reporter gene under the control of a human CYP3A4 promotor were seeded in a 384-well plate and cultivated at 37 °C and 5% CO_2_ in humidified air. Twenty-four hours prior to read-out, the cells were treated with the compound in a ten-step serial dilution of 1:3, starting at the highest test concentration of 50 μM and ending at 2 nM. Rifampicin was incubated in the same manner as the positive control. In addition, to normalize the luminescence signal, cells were incubated with Rifampicin at a concentration of 16.7 μM corresponding to 100% activation in addition to DMSO for background luminescence corresponding to 0% activation (*n* = 32 wells each). Cells were lysed and incubated with the Luciferase substrate ONE-Glo Reagent (Promega, Madison, WI) according to the manufacturer’s instructions, and the luminescence signal was detected in a plate reader. A concentration-dependent increase of the luciferase activity above 10% that of the rifampicin control was classified as PXR transactivation.

### *In Vivo* Exposure

All animal experiments were conducted in accordance with the German Animal Welfare Law and were approved by local authorities. Female NMRI nu/nu mice (Janvier, France) were dosed once at 100 mg/kg orally (*p.o.*, in PEG400/ethanol 9:1) (*n* = 3 per group), intraperitoneally (*i.p.*, in solutol/ethanol/water 4:1:5), or subcutaneously (*s.c.*, in castoroil/benzylbenzoate 9:1). After 0.5, 3, 6, 14, 24, and 48 h, mice were sacrificed by decapitation and blood sampled in potassium-EDTA tubes (Sarstedt, Germany). 100 μL of plasma was used for analysis. Samples were precipitated by the immediate administration of ice cold acetonitrile in a 1:5 dilution. Samples were frozen at −20 °C overnight and subsequently centrifuged at 3000 rpm for 15 min. The supernatant was analyzed with an Agilent 1200 HPLC system with MS/MS detection (AB Sciex, Framingham, MA). Obtained exposures were corrected for plasma–protein binding and reported in relation to the antiproliferative IC_50_u.

#### Structural Biology

For the production of recombinant PPARγ, codon- optimized DNA sequences of the human PPARG-LBD, including residues 231–505 (Uniprot P37231-1, Isoform 2), were synthesized (Gene Art, Life Technologies) and inserted into a modified pET22b vector for the overexpression of His-fusion proteins in *Escherichia coli*. All plasmids contained a thrombin protease cleavage site between the N-terminal 6× His tag and the protein of interest. The protein was overexpressed in *Escherichia coli* BL21(DE3) overnight at 17 °C after induction by IPTG.

In brief, cell pellets were resuspended in lysis buffer (20 mM pH 8.0 TRIS, 150 mM NaCl, 1 mM DTT, and 20 mM imidazole with EDTA complete (Roche Applied Science)) and mechanically lysed using a Microfluidizer. Lysates were centrifuged at 30 000 × *g* for 1 h, and clear supernatants were loaded on a 5 mL Protino Ni-NTA FPLC column (Macherey-Nagel GmbH). Bound protein was washed with buffer A (20 mM pH 8.0 TRIS, 150 mM NaCl, 1 mM DTT, and 20 mM imidazole) and high-salt buffer A (300 mm NaCl). The His or fusion tag was removed by thrombin cleavage on the column overnight at 16 °C. Cleaved PPARγ LDB and thrombin were eluted with buffer A. The final step of purification included the addition of benzamidine sepharose (Cytiva) to remove thrombin and size exclusion chromatography (10 mM pH 8.0 TRIS, 100 mM NaCl, 5 mM DTT, and 1 mM EDTA). The purified protein was concentrated and stored at −80 °C.

For crystallization and structure determination, purified human PPARγ LBD (9–12 mg/mL, frozen stock) was incubated with the peptide and the ligand at the indicated protein-to-peptide-to-ligand molar ratios (Table S1) for 1–5 h at room temperature. Small-molecule ligands were dissolved to 100 mM in DMSO and further diluted to 10 mM in ethanol. The NCOR2-ID2 peptide (H2343–W2365, HASTNMGLEAIIRKALMGKYDQW) was purchased (Biosyntan GmbH, Germany) and dissolved without further purification to 10 mM in protein buffer. All crystallization experiments were performed at 20 °C as sitting drops by adding equal volumes of sample and reservoir solution (100–300 nL). Crystallization conditions are listed in Table S1. Crystals were frozen in liquid nitrogen after cryo-protection with 25% glycerol.

Diffraction data were collected at the Proxima-2 beamline (Soleil synchrotron, Paris, France) and processed with XDS.^23^ See Table S1 for data and refinement statistics. The structures were solved using molecular replacement as implemented in Phaser and Dimple.^24^ Further refinement of the initial models was accomplished through multiple rounds of refinement in Refmac5^25^ and manual fitting and rebuilding in Coot.^26^ Restraints for small-molecule ligands were generated with ProDrg^27^ and modified for covalent linkage to a cysteine residue with JLigand.^28^

## Supporting Information Available

Methods for RNA sequencing and the PRISM multiplexed cell line panel, summary of the HTS screen, PRISM profiling, colony formation assay, RNA sequencing analysis, X-ray structural comparison of the binding modes of **BAY-4931** and T0070907, pharmacodynamic analysis of tumors treated with compounds, and HPLC traces of *in vivo* compounds (PDF)
X-ray statistics (CSV)
Detailed PK parameters (CSV)
Molecular string formulas and summary of the biochemical and cellular data (CSV)

## Accession Codes

PDB IDs for crystal structures as follows: 8AQM for PPARγ, NCOR2, and compound **6a** and 8AQN for PPARγ, NCOR2, and **BAY-4931**. RNA sequencing data are available from the National Center for Biotechnology Gene Expresison Omnibus (NCBI GEO) under accession no. GSE210693.

## Abbreviations Used

ADME: absorption, distribution, metabolism, and excretion
AUC_0-*t*last_: area under the curve until the last measurable time point
CRR: corepressor recruitment assay
CL_b_: clearance from blood
*C*_max_: maximum plasm concentration
*E*_max_: maximal efficacy
GSH: glutathione
MeCN: acetonitrile
NCOR: nuclear receptor corepressor
NHR-LBD: nuclear hormone receptor ligand-binding domain
RXR: retinoid X receptor
