# Correction to “Discovery and Structure-Based Design of Potent Covalent PPARγ Inverse-Agonists BAY-4931 and BAY-0069”

**DOI:** 10.1021/acs.jmedchem.2c02002

**Published:** 2022-12-21

**Authors:** Douglas L. Orsi, Elisabeth Pook, Nico Bräuer, Anders Friberg, Philip Lienau, Christopher T. Lemke, Timo Stellfeld, Ulf Brüggemeier, Vera Pütter, Hanna Meyer, Maria Baco, Stephanie Tang, Andrew D. Cherniack, Lindsay Westlake, Samantha A. Bender, Mustafa Kocak, Craig A. Strathdee, Matthew Meyerson, Knut Eis, Jonathan T. Goldstein

The incorrect PDF file was provided as Supporting Information with the published article. The correct file for this paper is provided here.

## Supporting Information Available

Methods for RNA sequencing and the PRISM multiplexed cell line panel, summary of the HTS screen, PRISM profiling, colony formation assay, RNA sequencing analysis, X-ray structural comparison of the binding modes of **BAY-4931** and T0070907, pharmacodynamic analysis of tumors treated with compounds, HPLC traces of *in vivo* compounds, and NOESY profiles of compounds **8b** and **8c** (PDF)

