# Supplementary material for: Discovery and Structure-Based Design of Potent Covalent PPARγ Inverse-Agonists BAY-4931 and BAY-0069
Source: J Med Chem. 2022 Oct 21;65(21):14843–63. doi: 10.1021/acs.jmedchem.2c01379 (PMC9662185; doi:10.1021/acs.jmedchem.2c01379)

Discovery and structure-based design of potent covalent PPARγ inverse-agonists, BAY-4931 and BAY-0069

Douglas L. Orsi,[1] Elisabeth Pook,[2] Nico Bräuer,[3] Anders Friberg,[3] Philip Lienau,[2] Christopher T. Lemke,[1] Timo Stellfeld,[3] Ulf Brüggemeier,[2] Vera Pütter,[3] Hanna Meyer,[3] Maria Baco,[4] Stephanie Tang,[4] Andrew D. Cherniack,[4,5] Lindsay Westlake,[4] Samantha A. Bender,[4] Mustafa Kocak,[4] Craig A. Strathdee[4], Matthew Meyerson,[4,5,6,7] Knut Eis,[2] and Jonathan T. Goldstein[4,*]

AUTHOR ADDRESS [1]Center for the Development of Therapeutics, Broad Institute of MIT and Harvard, Cambridge, MA, 02142, USA. [2]Research and Development, Pharmaceuticals, Bayer AG, 13353, Berlin, Germany. [3]Nuvisan ICB GmbH, 13353, Berlin, Germany [4]Cancer Program, Broad Institute of MIT and Harvard, Cambridge, MA, 02142, USA. [5]Department of Medical Oncology, Dana-Farber Cancer Institute, Boston, MA, 02215, USA. [6]Center for Cancer Genomics, Dana-Farber Cancer Institute, Boston, MA, 02215, USA. [7]Department of Genetics and Medicine, Harvard Medical School, Boston, MA, 02115, USA.

KEYWORDS: peroxisome proliferator-activated receptor, PPARG, inverse-agonist, nuclear receptor.

ABSTRACT: The ligand-activated nuclear receptor PPAR gamma (PPARG, PPARγ) represents a potential target for a new generation of cancer therapeutics, especially in muscle invasive luminal bladder cancer, where PPARγ is a critical lineage driver. Here we disclose the discovery of a series of chloro-nitro-arene covalent inverse-agonists of PPARγ that exploit a benzoxazole core to improve interactions with co-repressors NCOR1 and NCOR2. *In vitro* treatment of sensitive cell lines with these compounds results in robust regulation of PPARγ target genes and antiproliferative effects. Despite imperfect physicochemical properties, the compounds showed modest pharmacodynamic target regulation *in vivo*. Improvements to *in vitro* potency and efficacy of BAY-4931 and BAY-0069 compared to previously described PPARγ inverse agonists show that these compounds represent novel tools to probe the *in vitro* biology of PPARγ inverse agonism.

INTRODUCTION: Peroxisome-proliferator activated receptor gamma (PPAR gamma, PPARG, PPARγ) is a ligand-activated nuclear receptor and master regulator of adipogenesis[1]. PPARγ is the target of the glitazone (**1**) and glitazar (**2**) (Figure 1A) families of drugs used clinically for the treatment of lipid and glucose dysregulation associated with type 2 diabetes[2]. Activation of PPARγ through somatic alterations in *PPARG* gene and its partner protein *RXRA* are oncogenic[3-5], while high *PPARG* gene expression is the top predictive biomarker of *PPARG* genetic dependency in large-scale genome-wide genetic perturbation studies in the Cancer Dependency Map (DepMap.org)[6], with *PPARG* focal gene amplification (UM-UC-9 and 5637) and *RXRA* p.S427F hotspot mutation (HT1197) being outliers of particular interest. Additionally, pharmacological PPARγ agonists, including pioglitazone (**3**), are associated with an increased risk of bladder cancer[7-9]. Pharmacological antagonism of PPARγ *in vitro* has been shown to lead to antiproliferative effects in PPARγ-activated cancer cell lines[3], as well as promote osteogenesis[10], and genetic experiments are suggestive that PPARγ inverse agonists may induce proinflammatory effects in the tumor environment[4].

While most described PPARγ modulators are agonists, there are a small handful of described inverse-agonists, including T0070907 (**4**) and SR10221 (**5**)[10, 11] (Figure 1B), though none have progressed to clinical evaluation. To test the therapeutic hypothesis that inducing a repressive PPARγ complex using an inverse-agonist might be clinically beneficial for patients, we set out to identify potent and selective inverse-agonists with favorable pharmacokinetic and pharmacodynamic properties. Here we report the discovery of new potent and efficacious PPARγ covalent inverse-agonists. While these compounds are limited in their *in vivo* pharmacokinetic properties, they are valuable tools for *in vitro* studies.

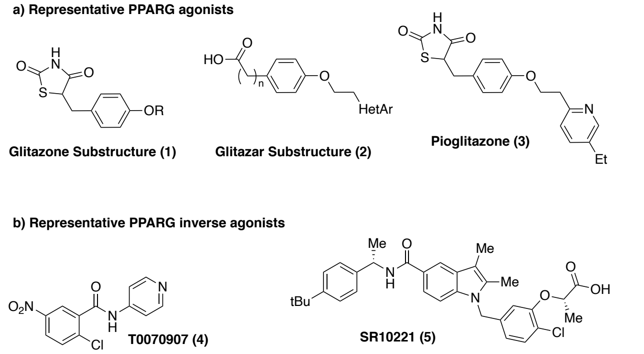

Figure 1. Representative PPARγ modulators. **A.** Representative PPARγ agonists with clinical utility. **B.** Representative PPARγ inverse-agonists.

RESULTS AND DISCUSSION: A high-throughput screening campaign for PPARγ inverse-agonists was undertaken as outlined in Figure 2A. A chemical library containing more than 4 million compounds was interrogated using an ultrahigh-throughput PPARγ Biochemical Competitive Binding Assay to identify binders of the PPARγ ligand binding domain (LBD) that are competitive the fluorescent labeled PPARγ ligand, fluormone™. Compounds that bind to the binding pocket of PPARγ LBD lead to a decrease in TR-FRET signal between fluormone and labeled PPARγ. Compounds were screened in duplicate at a single concentration (10μM). Candidate binders identified in the primary screen were validated by retesting in the PPARγ binding assay and a total of 15,152 primary hits and 11,421 confirmed hits for an overall positive rate of 0.3% (**Chart S1**) for PPARγ binders .

To deconvolute functional activity and prioritize confirmed binders, functional biochemical and cellular assays were performed at a single dose (10 μM) in a panel of assays. A PPARγ:MED1 TR-FRET biochemical co-activator interaction assay was used to measure the effect of compounds on the interaction between PPARγ LBD and a peptide containing the "LxxLL" nuclear receptor interaction motif from the co-activator, MED1 (TRAP220/ DRIP-205). A signal increase is indicative of a potential agonist and known PPARγ agonist GW1929[12] responded accordingly. To investigate candidate inverse-agonists, the assay was adapted to use a peptide containing the interaction motif from the co-repressor, NCOR2 (Smrt ID2)[13] in place of MED1 peptide. Compounds that induce interactions between PPARγ LBD and peptide from the co-repressor NCOR2 would be indicative of inverse-agonists. Known inverse-agonists T0070907[11] and SR10221[10] responded accordingly.

A large proportion of the initially confirmed HTS candidates did not show activity in the coregulator recruitment assays (bottom left quadrant of Fig 2B) and are likely simple binders or neutral antagonists. Another large fraction overlapped in directionality of assay signal with the PPARγ agonist, GW1929, in the upper left quadrant of Figure 2B. PPARγ inverse-agonists T0070907 and SR10221 demonstrated a concentration-dependent increase in signal in the NCOR2 biochemical assay. A small number of candidates led to a signal increase in the co-repressor recruitment assay (lower right quadrant of 2B), indicating potential inverse-agonists. Interestingly, agonists led to a decreased signal in the inverse-agonist biochemical assay with NCOR2 peptide, and conversely, inverse-agonists decreased signal in the agonist assay with MED1 peptide. This indicates that inverse-agonists may induce a structural conformation that disrupts basal interactions between PPARγ and co-activators, further shifting the equilibrium from activation to repression. Whether this also occurs in the cellular context is unclear. To eliminate possible assay artifacts and verify activity in a cellular context, high-throughput RT-qPCR monitoring the mRNA transcripts of the canonical PPARγ target gene *FABP4* was performed. The effects of compounds on the expression of *FABP4* in RT112 cells were also monitored using a high-throughput nanoluciferase reporter assay (RT112-FABP4-NLucP[3]) to evaluate and triage candidate compounds based on cellular activity with a single dose of 10μM.

Subsequent predictive filtering of chemical structures to eliminate undesirable structures refined the list to 561 preferred candidates for testing in dose-response format. Preferred hits were evaluated in a co-repressor recruitment TR-FRET assay measuring ligand dependent changes in the interaction between PPARγ and a peptide from NCOR2, in addition to RT-qPCR of the canonical PPARγ target gene, *FABP4*. Compounds were also tested in a previously described cellular reporter assay for PPARγ transactivation, RT112-FABP4_NLucP reporter assay[3].

To select the most suitable starting point for a candidate optimization effort, we started with an absolute requirement for confirmed binding with a dose-dependent increase in signal in the PPARγ:NCOR2 interaction assay. Candidates were further triaged by a requirement for dose-dependent inverse-agonist activity in one or more of the cellular assays in PPARγ-activated bladder cancer cell lines, including RT-qPCR in RT112 cells, or RT112-FABP4-NLucP. Five favored chemotypes were identified. One promising candidate, **6a**, which shares the same chloro-nitro benzamide covalent warhead as T0070907 (Compound 4), was selected for optimization.

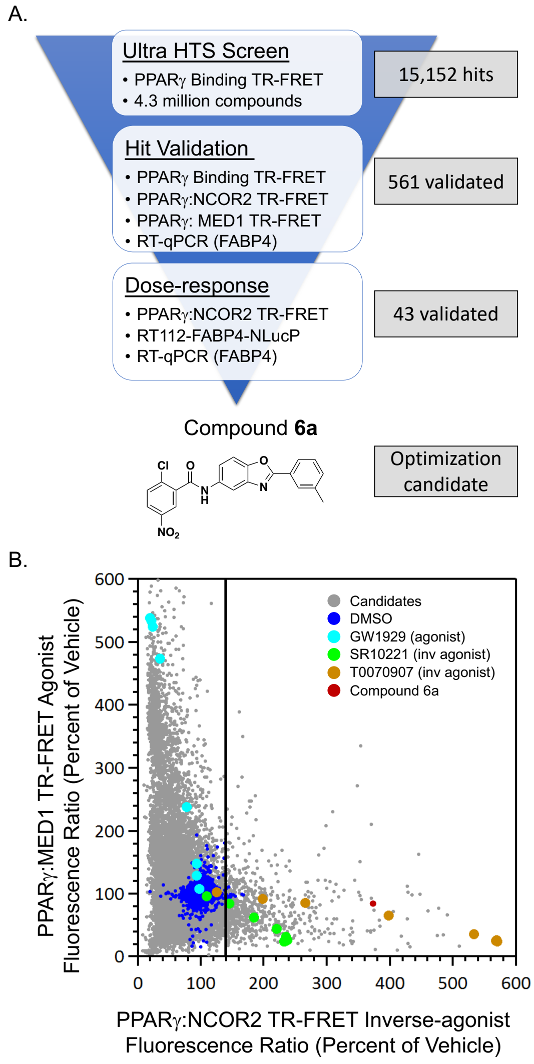

Figure 2 Discovery of PPARγ inverse-agonists. A. Representation of hit finding screening cascade from ultrahigh-throughput competitive ligand-binding assay of 4.3 million compounds, single

concentration hit validation, and dose-response curves to identify candidate hit 6a. B. Hit validation comparing fluorescent signal from PPARg:MED1 co-activator recruitment and PPARg:NCOR2 co-repressor recruitment (CRR) LanthaScreen TR-FRET interaction assays for candidate compounds tested at 10µM. PPARg probe compounds were added in dose-titration for benchmarking assay performance. Probe compounds included GW1929 (agonist), SR10221 (inv agonist), and T0070907 (inv agonist).

To help guide medicinal chemistry efforts, **6a** was co-crystallized with the PPARγ LBD bound to a peptide derived from the co-repressor NCOR2 (Figure 3). **6a** covalently modified C313 of PPARγ isoform 2, in a similar manner to T0070907[14]. Crystal structures of **6a** and T0070907 show that Helix-12, an essential interaction surface for co-activator proteins, is sequestered into the canonical ligand binding site and is thus unavailable to recruit the co-activators (Fig 3B). The conserved residues of the hydrophobic receptor interaction motif (LXXIIXXXL) of NCOR2 are key to the interaction with PPARγ bound to the inverse-agonists (Fig 3C). Intriguingly, the C-terminal residue (Y475 in PPARγ isoform 1 NP_005028, or Y505 in PPARγ isoform 2; NP_056953.2) interacts directly with the amide linker of the inverse-agonist.

Compared to known inverse-agonist T0070907, the lipophilic tail of **6a**, consisting of the benzoxazole and 3-tolyl group, extends further towards the lipophilic co-repressor binding surface. The binding region is relatively narrow, with the potential to interact with solvent around the central benzoxazole ring. To systematically investigate the insights gleaned from this crystal structure, the structure activity relationship (SAR) for the compound was evaluated within 3 structural regions (Scheme 1), the NCOR-interacting ring, the potentially solvent-interacting core, and the covalent warhead.

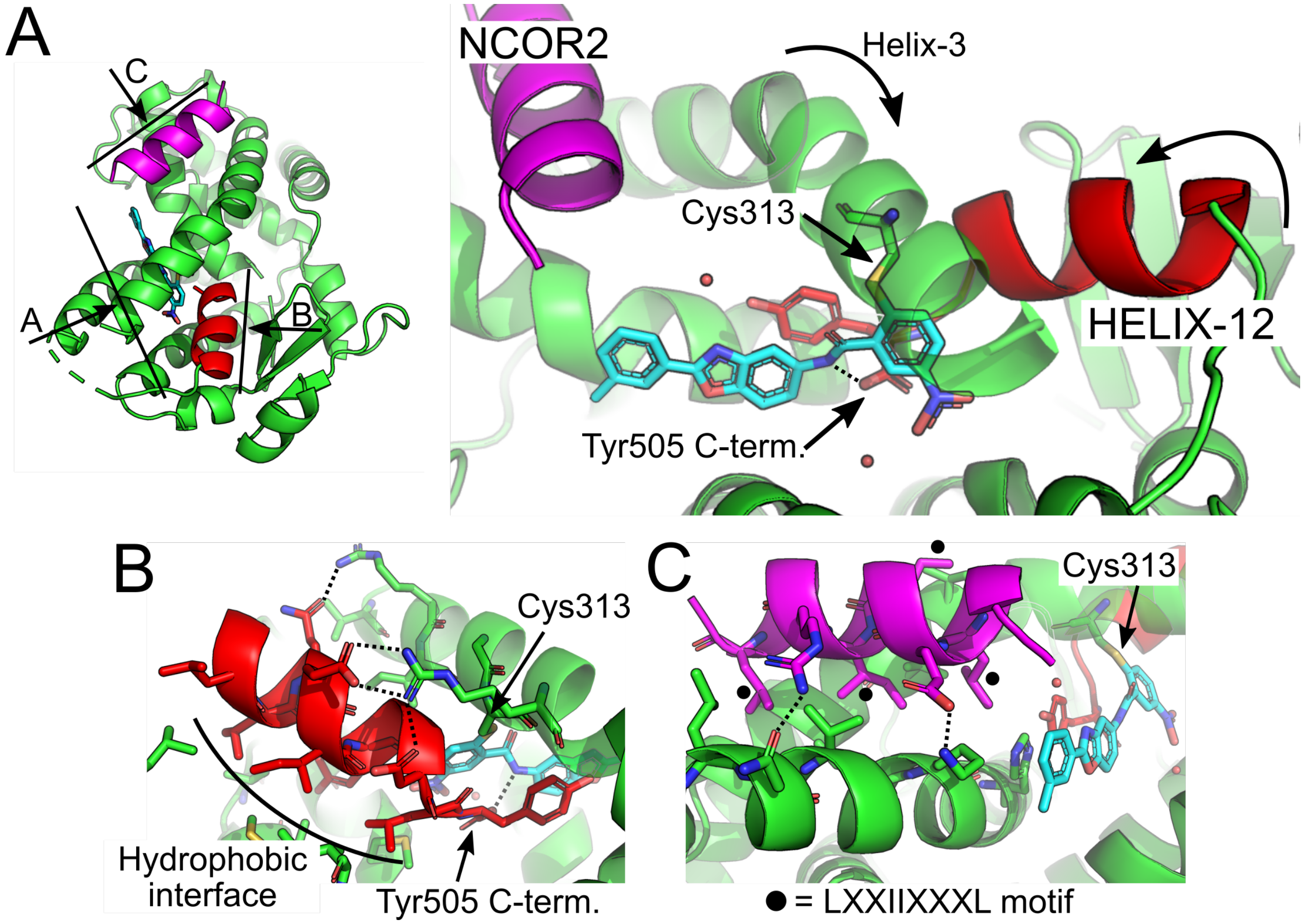

Figure 3. Crystal structure of PPARγ bound to NCOR2 co-repressor peptide and the inverse-agonist compound **6a** (PDB ID: 8AQM). **A.** Left: Insert showing the complete trimeric complex with the views of the different panels indicated by lines and arrows. Right: Overview of the PPARγ (green) co-complex with the C-terminal Helix-12 (red) bound behind the kinked Helix-3, NCOR2 peptide (magenta), and compound **6a** (cyan) covalently bound to Cys313. The very C-terminus, Tyr505, interacts directly with **6a**. **B.** Intramolecular interactions of Helix-12 when sequestered into the canonical ligand binding site. **C.** NCOR2 co-repressor binding interactions to PPARγ.

SAR: Initial efforts focused on improving the interactions between compounds and NCOR2. Compounds with variable NCOR-interacting rings were synthesized according to **Scheme 1**. Synthesis of the necessary anilines was achieved by either reduction of the corresponding nitro-arene under $SnCl_2$ conditions or via a Buchwald-Hartwig coupling of the corresponding aryl-bromide with tert-butyl carbamate, followed by Boc deprotection with HCl in 1,4-dioxane. The resulting anilines were then reacted with the aryl chloride of the corresponding covalent warhead to provide the desired products.

Scheme 1. SAR Strategy and general synthesis of target compounds

a) Planned SAR Exploration

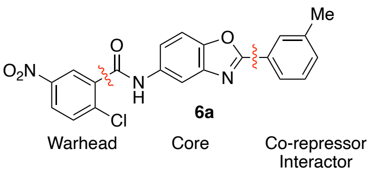

b) General Synthetic Route

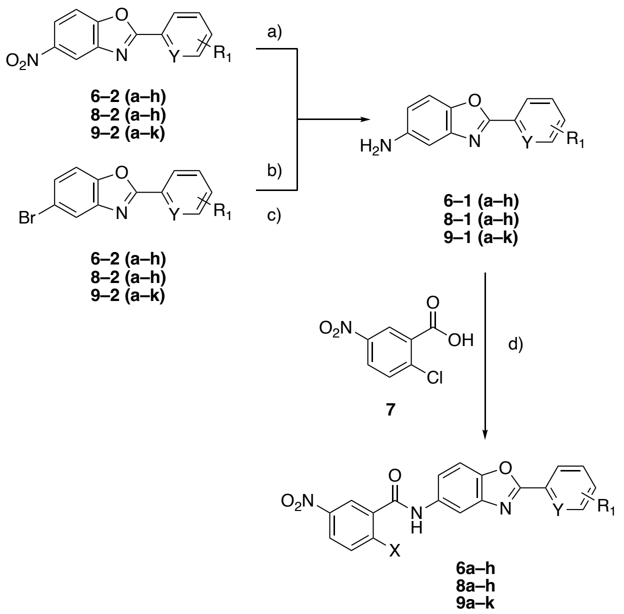

a) $SnCl_2$ (4 equiv), EtOH, reflux. b) t-Bu carbamate (1.2 equiv), NaO(t-Bu) (2 equiv), t-Bu XPhos (0.1 equiv), $Pd(dba)_2$ (0.03 equiv), PhMe, 60 ˚C. c) 4 M HCl in 1,4-dioxane, 25 ˚C. d) i) **7** (1.03 equiv), $SOCl_2$ (excess), 80 ˚C, concentrated. ii) **6–1 (a–h)** (1 equiv), Et_3N (5 equiv), THF, 25 ˚C.

A variety of small substituents on the aryl ring at different positions showed strong preference for meta / para substitution (**6a, 6c (BAY-4931)**), while ortho substitution (**6b**) dramatically reduced potency and maximal efficacy in the cellular assays (**Table 1**). Incorporation of electron-poor rings (**6d-e**) significantly improved activity, though combining bulk at the para position with an electron deficient ring did not provide an additive effect **(6f**). Substituted 3-pyridines maintained activity in the co-repressor recruitment assay **(6g-h) (Table1).**

Functional activity was evaluated by testing candidate compounds in NCOR2 recruitment assay as well as the previously described RT112-FABP4-NLucP cellular reporter assay for PPARγ transactivation[3]. Again, para substitution was strongly preferred (**BAY-4931**), while the combination of the 3-pyridyl and 4-ethyl substitutions led to favorable effects in cellular assays **(6h).** Notably, the variability in maximal efficacy (E_{max}) in the cellular assays was less pronounced than in the biochemical assay. To profile the antiproliferative effect of functional inverse-agonists, compounds were evaluated in UM-UC-9 bladder cancer cells, which possess a focal gene amplification of *PPARG* to greater than approximately 25 copies and are exquisitely sensitive to PPARγ modulation[3]. Again, para substitution was strongly preferred, as only (**BAY-4931**) and (**6h**) demonstrated compelling antiproliferative potency and efficacy (**Table 1**). Interestingly, **6g** maintained potency, but had poor efficacy in the UM-UC-9 proliferation assay indicative of a partial inverse-agonist.

Table 1: SAR of the Co-repressor interacting aryl group

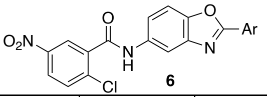

Cmpd	Ar	CRR[a] EC_{50} [nM] / E_{max} [%]	Reporter[b] IC_{50} [nM] / E_{max} [%]	UM-UC-9 Proliferation[c] IC_{50} [nM] / E_{max} [%]
**6a**	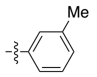	1.8 / 345	1300 / 87	>10000 / 0
**6b**	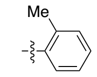	5.2 / 99	195 / 52	6640 / -
**6c (BAY-4931)**	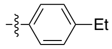	5.8 / 619	0.17 / 94	3.4 / 79
**6d**	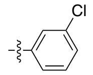	7.2 / 509	52 / 93	– / –
**6e**	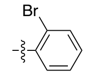	3.9 / 235	480 / 133	– / –
**6f**	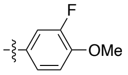	2.2 / 534	0.23 / 82	314 / 67
**6g**	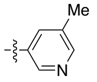	0.65 / 437	0.16 / 72	1.9 / 34
**6h**	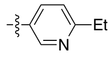	2.0 / 580	0.25 / 107	3.1 / 86

a) LanthaScreen TR-FRET PPARγ co-repressor recruitment (CRR) assay with PPARγ -LBD and NCOR2 (Smrt ID2) peptide. Refer to the Experimental Section for assay conditions. EC_{50} and E_{max} values represent the values from one or more experiment performed with samples in duplicate. b) RT112-FABP4-NLucP cellular reporter assay for transactivation of PPARγ. Refer to the Experimental Section for assay conditions. IC_{50} and E_{max} values represent the mean from two or more experiments. c) UM-UC-9 proliferation assay. Refer to the Experimental Section for assay conditions. IC_{50} and E_{max} values represent mean one or more experiments read out at day 7.

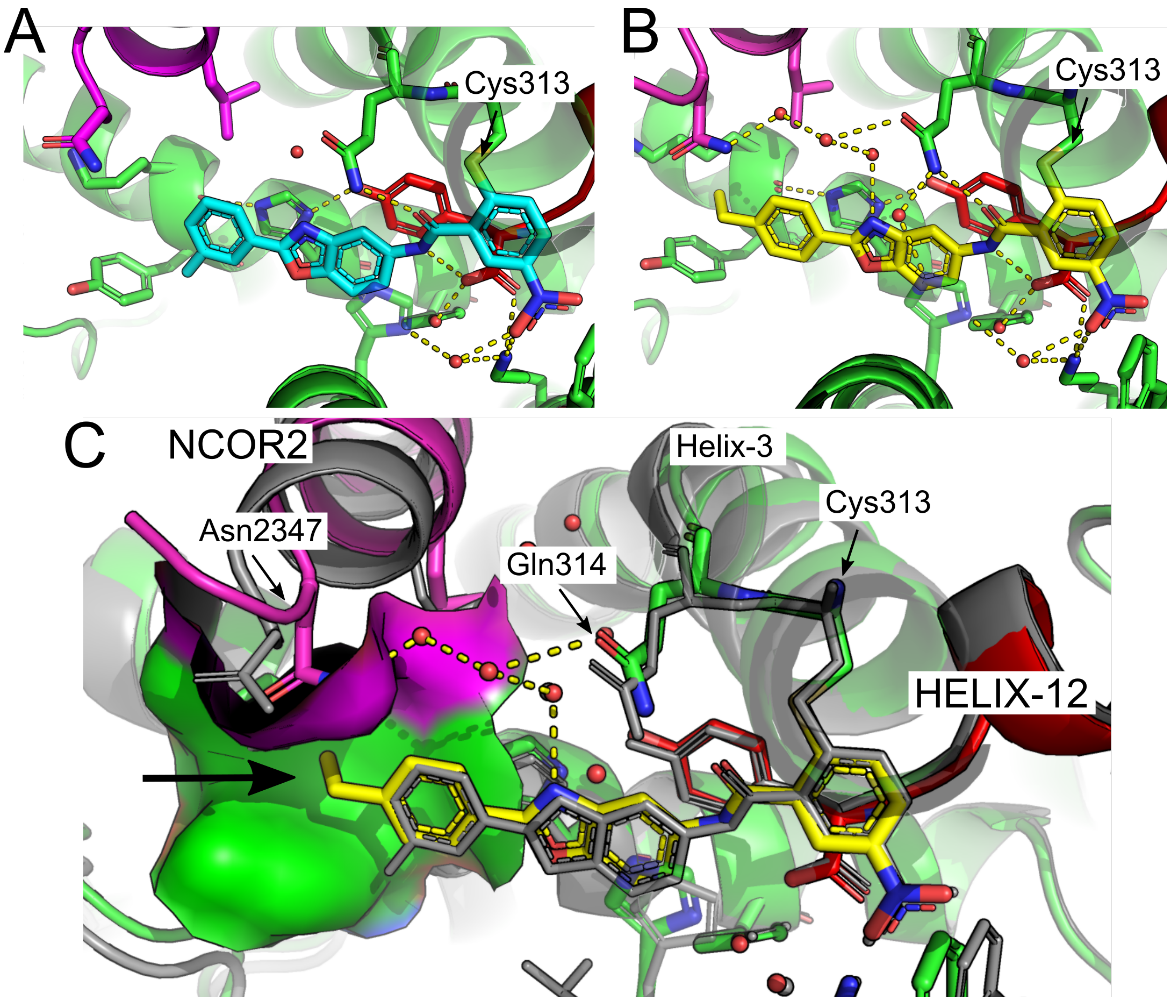

Figure 4. Structural details of the interactions between PPARγ /NCOR2 and **6a** (PDB ID: 8AQN) and the optimized inverse-agonists BAY-4931 (PDB ID: 8AQN). **A.** Co-crystal structure of PPARγ (green), NCOR2 peptide (magenta), and **6a** (cyan). Polar interactions are highlighted with yellow dashes. **B.** Co-crystal structure of PPARγ (green), NCOR2 peptide (magenta), and BAY-4931 (yellow). **C.** Comparison of the crystal structures with **6a** (gray) and BAY-4931 (colored as in B). A water-mediated interaction between BAY-4931, Gln314, and Asn2347 of the NCOR2 peptide is highlighted. The additional hydrophobic interaction of the para ethyl group of BAY-4931 is indicated by a black arrow (lower left).

The increased E_{max} observed in the corepressor recruitment assay by para-substituted compounds may be explained by increased and/or stabilized interactions with the NCOR2 peptide. To test this hypothesis, **BAY-4931** was co-crystallized with PPARγ (Fig 4). The covalently bound inverse-agonist, **6a**, extends towards the NCOR2 peptide and is located slightly underneath the N-terminus of the co-repressor (Fig 4A). Para-substituted **BAY-4931** shows hints of stabilized recruitment of the NCOR2 peptide (Fig 4B). The NCOR2 peptide exhibits a modest shift in position and two additional N-terminal residues of the peptide could be modeled with confidence. The introduced ethyl substitution fits well into a niche formed between the receptor and the co-repressor. Also, with BAY-4931, a more extensive water-network was observed, now extending all the way from PPARγ /BAY-4931 to the NCOR2 peptide. Comparison of the two complexes otherwise show minimal changes in ligand binding mode and protein conformations (Fig 4C). Extending the N-terminus of the NCOR2 peptide did not change any of the observed interactions or increase the number of resolved residues. Taken together, this is in line with the biochemical and cellular data of the two compounds (**Table 1**) showing **BAY-4931** is more potent and efficacious than **6a**. The hypothesis that a stabilized binding mode of the inverse-agonist is important for increased efficacy fits with the finding that T0070907 exhibits conformational dynamics and a different binding mode when bound to PPARγ prior to recruitment of NCOR2 as described by Shang *et al*[14] (PDB: 6C1I). The observed flipped binding mode of T0070907 does not allow for any interactions to NCOR2 and Helix-12 is also not sequestered (**Figure S4**).

Selected active compounds from **Table 1** were profiled for metabolic stability, Caco-2 permeability, aqueous solubility, glutathione (GSH) stability and extent of covalent modification of the PPARγ ligand binding domain (**Table 2**). All compounds tested were poorly soluble and highly metabolized in rat liver hepatocytes (≥ 70% of LBF in rats assuming 4.2 L/h/kg[15]), but moderately stable in human liver microsomes (LBF in human of ~1.3 L/h/kg[16]) (**Table 2**). Permeability ranged from low (≤ 10 nm/s, **6c**) to favorable (≥ 70 nm/s, **6g**), with none of the compounds tested showing signs of P-glycoprotein (P-gp) mediated efflux (**Table 2**). Despite improved human microsomal stability and permeability for

pyridines **6g** and **6h**, these compounds did not lead to an improvement in rat hepatocyte stability or solubility compared to **BAY-4931**. Thus **BAY-4931** was selected as the preferred compound for further optimization.

Table 2: ADME of selected NCOR2 interacting ring modifications

Cmpd	$Cl_{h, mic}$ (L/h/kg)[a]	$Cl_{r, hep}$ (L/h/kg)[b]	GSH Stability (% recovery 1 / 2 / 4 / 24 h)[c]	Cys Stability (% recovery 1 / 2 / 4 / 24 h)[d]	Caco-2 Permeability A–B (nm/s) / Efflux Ratio[e]	Solubility (mg/L)[f]
**6a**	0.45	4.2	100 / 100 / 17 / 13	100 / 85 / 88 / 15	11.62 / 0.36	<0.1
**BAY-4931**	0.82	3.7	93 / 76/ 68 / 21	87 / 78 / 67 / 12	1.34 / 0	0.27
**6f**	0.81	4.1	100	100	18 / 0.10	<0.1
**6g**	0.31	4.2	100	100	170 / 0.41	<0.1
**6h**	0.61	4.1	–	–	–	<0.1

a) Human microsomal stability determined by the incubation of 1 µM of compound with human liver microsomes for 1 h. Refer to Experimental Section for assay conditions. Clearance value represents one experiment. b) Rat hepatocyte stability assay determined by the incubation of 1 µM of compound with rat liver hepatocytes for 1.5 h. Refer to Experimental Section for assay conditions. Clearance value represents one experiment. c) Compound stability in buffer containing 500 µM of glutathione. Refer to Experimental Section for assay conditions. % recovery represents one experiment. d) Compound stability in buffer containing 500 µM of cysteine. Refer to Experimental Section for assay conditions. % recovery represents one experiment. e) Caco-2 permeability assay. Refer to Experimental Section for assay conditions. Permeability and efflux ratio represent a single experiment. f) Thermodynamic solubility of compound in pH 6.5 PBS buffer from a DMSO stock. Refer to Experimental Section for assay conditions.

We next turned our attention to the potentially solvent exposed central core to improve physicochemical and ADME properties. Compounds with variable central cores were synthesized according to **Scheme 1b.**

Inverting the benzoxazole regiochemistry **(8a)** maintained co-repressor recruitment, whereas making the benzoxazole the NCOR-interactor and the aryl the linker ablated co-repressor recruitment (**8g, h**) (**Table 3**). Benzannulated heteroaryl cores provided mixed results, as N-Me benzimidazoles reduced co-repressor recruitment (**8b, c**) while an imidazo-pyridine and a benzotriazole retained co-repressor recruitment (**8d, e**) (**Table 3**). Efforts to replace the amide linker with a sulfonamide failed to retain activity (**8f**). Modifications that retained biochemical activity resulted in unacceptable loss in efficacy and potency in the cellular assays.

Table 3: Central core SAR

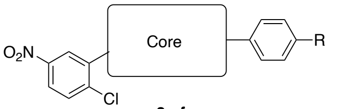

8a-f

Cmpd	Ar	CRR[a] EC_{50} [nM] / E_{max} [%]	Reporter[b] IC_{50} [nM] / E_{max} [%]	UM-UC-9 Proliferation[c] IC_{50} [nM] / E_{max} [%]
**BAY-4931**	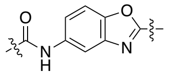	5.8 / 619	0.17 / 94	3.4 / 79
**8a, R=Me**	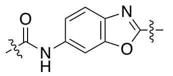	4.0 / 524	1800 / 68	3710 / 105
**8b, R=Et**	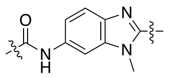	19 / 424	3.3 / 74	9.2/ 36
**8c, R=Et**	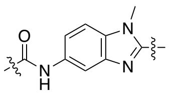	3.7 / 95	4.4 / 54	913 / –
**8d R=H**	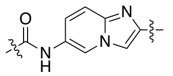	2.6 / 647	0.58 / 89	52 / 55
**8e R=Me**	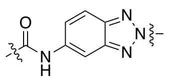	4.4 / 562	0.32 / 93	2.8 / 36
**8f, R=Et**	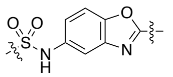	>5000 0 / 0	>5000 0 / 0	– / –
**8g**	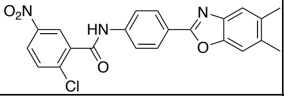	10 / 288	1.6 / 95	8.8 / 43
**8h**	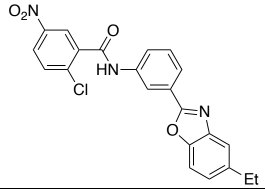	54 / 331	>5000 0 / 0	>1000 0 / 0

a) LanthaScreen TR-FRET PPARγ co-repressor recruitment assay with NCOR2 (Smrt ID2) peptide. Refer to the Experimental Section for assay conditions. EC_{50} and E_{max} values represent the mean from at least two experiments with points tested in duplicate. b) RT112-FABP4-NLucP cellular reporter assay for transactivation of PPARγ. Refer to the Experimental Section for assay conditions. IC_{50} and E_{max} values represent the mean from two or more experiments with points tested in quadruplicate. c) UM-UC-9 proliferation assay. Refer to the Experimental Section for assay conditions. IC_{50} and E_{max} values represent mean one or more experiments read out at day 7.

Despite the lack of improvement in cellular assays, selected compounds from **Table 3** were profiled in tier 1 ADME to understand the impact of increased polarity on pharmacokinetic properties. Notably, the more polar N-heteroaryl cores failed to improve the solubility or metabolic stability of the parent compound, though **8c** and **8d** demonstrated improved permeability (**Table 4**). Since no modifications made thus far improved hepatocyte stability, **compound 6a** was subjected to metabolite identification studies. The sole identified metabolite of **6a** results from GSH displacing the Ar-Cl warhead (**Figure 5**). Envisioning that improving the metabolic stability was essential to continue to progress the chemical series, we next modulated the reactivity of the covalent warhead.

Table 4: Tier 1 ADME of selected compounds from Table 3

Cmpd	$CL_{b, h\ mic}$ (L/h/kg)	$CL_{b, r\ hep}$ (L/h/kg)	GSH Stability % remaining 1 / 2 / 4 / 24 h	Cys Stability % remaining 1 / 2 / 4 / 24 h	Caco-2 Permeability A–B (nm/s) / Efflux Ratio	Solubility (mg/L)
**BAY-4931**	0.82	3.7	93 / 76 / 68 / 21	87 / 78 / 67 / 12	1.3 / 0	0.27
**8b**	0.61	4.2	100 / 83 / 67 / 38	100 / 92 / 77 / 62	– / –	<0.1
**8c**	0.81	4.1	100	100	94 / 0.36	<0.1
**8d**	0.31	4.2	100	100	71 / 0.32	<0.1
**8e**	0.61	4.1	97 / 82 / 63 / 7.8	93 / 74 / 71 / 4.7	3.7 / 0	N/A

a) Human microsomal stability determined by the incubation of 1 µM of compound with human liver microsomes for 1 h. Refer to Experimental Section for assay conditions. Clearance value represents one experiment. b) Rat hepatocyte stability assay determined by the incubation of 1 µM of compound with rat liver hepatocytes for 1 h. Refer to Experimental Section for assay conditions. Clearance value represents one experiment. c) Compound stability in buffer containing 500 µM of glutathione. Refer to Experimental Section for assay conditions. % Recovery represents one experiment. d) Compound stability in buffer containing 500 µM of cysteine. Refer to \ Experimental Section for assay conditions. % Recovery represents one experiment. e) Caco-2 permeability assay. Refer to Experimental Section for assay conditions. Permeability and efflux ration represent a single experiment. f) Thermodynamic solubility of compound in pH 6.5 PBS buffer from a DMSO stock. Refer to Experimental Section for assay conditions.

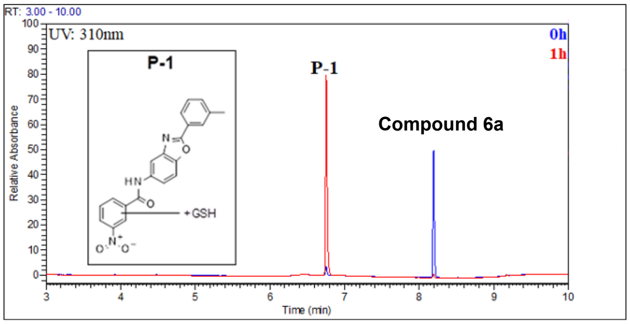

Figure 5. Identification of compound **6a** metabolic reaction products by incubation with rat hepatocytes. UV chromatogram of **6a** and its metabolite P-1 before (blue) and after 1 h incubation (red) in rat hepatocytes and structure proposal of the metabolite P-1 based on the exact masses of the metabolite P-1 and its MS-MS fragments

Less-reactive warheads decreased activity in PPARγ activity assays (**Table 5**). Changing the activating nitro group to a variety of less electron withdrawing groups (**9a-d**) caused significant or full activity loss, though nitrile **9d** remained active in the co-repressor recruitment assay. Removing or positionally shifting the covalent warhead resulted in inactive compounds (**9e, f**). Warheads exploiting alternate chemistry, such as heteroarenes and acrylamides, were inactive (**9g, h**). Aryl halides maintained similar in vitro activity to the parent chloride **BAY-4931 (9i–k**). However, these substitutions failed to improve metabolic stability (**Table 6**). Despite poor aqueous solubility, low metabolic stability, and low permeability (see **Table 6**), chloride **BAY-4931** and bromide **9j (BAY-0069)** possessed the best overall *in vitro* profile; thus, both were characterized *in vivo*.

Table 5: Warhead SAR

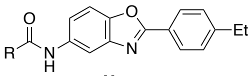

Y

Cmpd	Ar	CRR[a] EC_{50} [nM] / E_{max} [%][a]	Reporter[b] IC_{50} [nM] / E_{max} [%]	UM-UC-9 Proliferation[c] IC_{50} [nM] / E_{max} [%]	Covalent Binding[d] [%]
**BAY-4931**	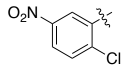	5.8 / 619	0.17 / 94	3.4 / 79	88
**9a**	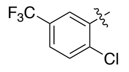	>50000 / 26	>50000 / 0	– / –	N/A
**9b**	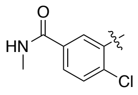	>50000 / 0	>33000 / 0	– / –	N/A
**9c**	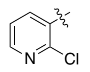	>50000 / 0	>50000 / 0	– / –	N/A
**9d**	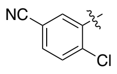	87 / 335	8.6 / 113	358 / 84	95
**9e**	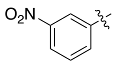	8050 / 554	>50000 / 0	– / –	N/A
**9f[e]**	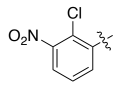	>50000 / 0	>50000 / 0	1220 / 22	91
**9g**	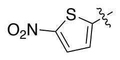	7950 / 86	9350 / 97	– / –	N/A
**9h**	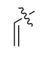	>50000 / 16	5750 / 94	– / –	10
**9i**	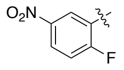	3.3 / 638	4.0 / 77	330 / 91	9.5
**9j (BAY-0069)**	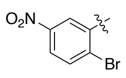	9.6 / 620	0.22 / 88	2.5 / 72	59
**9k**	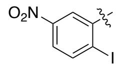	28 / 690	0.29 / 115	5.3 / 78	94

a) CRR LanthaScreen TR-FRET PPARγ:NCOR2 assay with NCOR2 (Smrt ID2) peptide. Refer to the Experimental Section for assay conditions. EC50 and Emax values represent the mean from at least two experiments. b) RT112-FABP4-NLucP cellular reporter assay for transactivation of PPARγ. Refer to the Experimental Section for assay conditions. IC_{50} and E_{max} values represent the mean from at least two experiments. c) UM-UC-9 proliferation assay. Refer to the Experimental Section for assay conditions. IC_{50} and E_{max} values represent mean one or more experiments. d) Relative covalent binding assay. Refer to the Experimental Section for assay conditions. [%] represents relative amount of protein showing covalent adduct MW shift. e) With 3-methylphenyl NCOR2 interacting ring.

Table 6: ADME profile of selected warheads

Cmpd	$CL_{b, h\ mic}$ (L/h/kg)	$CL_{b, r\ hep}$ (L/h/kg)	GSH Stability % remaining 1 / 2 / 4 / 24 h	Cys Stability % remaining 1 / 2 / 4 / 24 h	Caco-2 Permeability A–B (nm/s) / Efflux Ratio	Solubility (mg/L)
**6c BAY-4931**	0.82	3.7	93 / 76 / 68 / 21	87 / 78 / 67 / 12	1.3 / 0	0.27
**9d**	0.80	3.5	—	—	0 / 0	<0.1
**9i**	0.78	3.9	73 / 36 / 17 / 3.7	100 / 100 / 78 / 52	– / –	<0.1
**9j BAY-0069**	0.47	3.9	62 / 36 / 17 / 3.7	84 / 46 / 12 / –	2.6 / 0.24	<0.1
**9k**	0.67	3.9	–	–	3.3 / 0	<0.1

a) Human microsomal stability determined by the incubation of 1 μM of compound with human liver microsomes for 1 h. Refer to Experimental Section for assay conditions. Clearance value represents one experiment. b) Rat hepatocyte stability assay determined by the incubation of 1 μM of compound with rat liver hepatocytes for 1 h. Refer to Experimental Section for assay conditions. Clearance value represents one experiment. c) Compound stability in buffer containing 500 μM of glutathione. Refer to Experimental Section for assay conditions. % Recovery represents one experiment. d) Compound stability in buffer containing 500 μM of cysteine. Refer to Experimental Section for assay conditions. % Recovery represents one experiment. e) Caco-2 permeability assay. Refer to Experimental Section for assay conditions. Permeability and efflux ration represent a single experiment. f) Thermodynamic solubility of compound in pH 6.5 PBS buffer from a DMSO stock. Refer to Experimental Section for assay conditions.

Selectivity profile of BAY-4931 and BAY-0069: *PPARG* is a member of a family of lipid-activated nuclear receptors, which encompasses *PPARA* and *PPARD*, with additional high homology with PXR. As such, **BAY-4931** and **BAY-0069** were tested in cellular reporter activity assays for mouse Pparγ to determine cross-species reactivity, as well PPARA, PPARD, and PXR to determine selectivity (**Table 7**). **BAY-0069** only acted as an inverse-agonist at PPARγ, with little activity at the homologous PXR. When tested for CYP inhibition across a panel of the most relevant CYP enzymes for drug-drug-interactions, both **BAY-4931** and **BAY-0069** only inhibited CYP2C8 (**Table 7**).

Table 7: Selectivity and metabolic liability profile of BAY-4931 and BAY-0069

	GAL4-NHR-FLUC IC_{50} [nM] / E_{max}[%]				CYP Inhibition IC_{50} [µM]					PXR NOEL[j]
Compound	mouse Pparγ[a]	PPARγ[b]	PPARA[c]	PPARD[d]	CYP1A2[e]	CYP2C8[f]	CYP2C9[g]	CYP2D6[h]	CYP3A4[i]	[µM]
BAY-4931	0.14/82	0.40/100	>50000/0	>50000/0	>10	7.0	>10	>10	>10	> 50
BAY-0069	24/25	6.3/72	7500/63	9000/84	>5	4.3	>5	>5	>5	41

a-d) GAL4-NHR-LBD one hybrid reporter assay with GAL4 DNA binding domain fused to a nuclear hormone receptor ligand binding domain (NHR-LBD). Refer to the Experimental Section for assay conditions. IC50 (nM) and E_{max} (% inhibition of luciferase reporter activity) or activation (+) values are from one experiment with a 10 point dose-response curve with 4 replicates per point for a) mouse Pparγ, b) human PPARγ, c)PPARA, and d) PPARD. e-i) IC50 (µM) values for CYP inhibition assays with specific isoform indicated. Data represent the mean from one experiment performed in duplicate. j) PXR (NR1I3) no effect level IC_{50} (µM).

***In vitro* cell profiling:** Compounds **BAY-4931** and **BAY-0069** were compared to probe compounds, T0070907 and SR10221, in dose response across a variety of cell lines. These studies highlight subtle differences of the *in vitro* profiles of these newly described compounds (**Figure 6A-E**). A dose dependent increase in the interaction between PPARγ and co-repressor peptides from NCOR1 and NCOR2 were observed as expected for inverse-agonists. A dose-dependent decrease in the interaction with peptide from co-activator MED1 was also observed, even in the absence of exogenous agonist, indicating a destabilizing effect of inverse-agonists on basal interactions between PPARγ and co-activators. BAY-4931 and BAY-0069 led to antiproliferative effects in *PPARG* amplified cell line UM-UC-9, while agonist rosiglitazone led to a modest, but reproducible increase in proliferation in this cell line.

During kinetic proliferation profiling in sensitive bladder cell lines[3], we noticed a delay of 2-5 days after initial dosing prior to observing changes in the proliferation rate and eventual cytostasis. Therefore, we tested the effects of T0070907 or BAY-4931 on proliferation in a 12-day multiplexed cell line panel screen to screen for potentially sensitive cell lines (PRISM, Broad Institute, **Figure S1**)[17]. Candidate cell lines from this study in addition to other *PPARG*-dependent cell lines from DepMap were selected for further evaluation in colony formation assays to enable extended treatment with compounds for 7-14 days (**Figure 6F and Figure S2**). We compared the antiproliferative effects of inverse-agonists T0070907 and BAY-4931 to the neutral antagonist GW9662. At a concentration of 100nM, BAY-4931 shows antiproliferative effects across the majority of the cell lines selected for predicted sensitivity. At the same concentration, T0070907 also shows antiproliferative effects in these cell lines, but to a more modest degree. UM-UC-9, HuP-T4, and PaCaDD-188 were among the most sensitive cell lines in the colony formation assays treated with BAY-4931. BFTC905, a bladder cell line, was chosen as a control as it was not identified as sensitive to compounds in the PRISM panel or genetic perturbation of *PPARG* in DepMap and compounds had minimal effect; the lack of growth inhibition in BFTC905 by the inverse-agonists taken together with antiproliferative effects in the predicted sensitive cell lines suggests compound selectivity for the subset of *PPARG*-dependent cell lines.

***In vitro* effects on gene expression:** The effects of PPARγ modulators on global mRNA regulation were evaluated by RNA sequencing in the *RXRA* p.S427F hotspot mutation bladder cell line, HT-1197 (**Figure S3**). Comparing treatment effects of inverse-agonists T0070907 at 500nM to BAY-4931 at 200nM, it was observed that the same genes are regulated by T0070907 and BAY-4931 with the same directionality of the effect. The genes expression effects anti-correlate with the effects of the PPARγ agonist, rosiglitazone (**Figure S3a**). Furthermore, the entire gene set was regulated proportionately more with BAY-4931 (**Figure S3b**) under the conditions tested. This indicates that at maximal receptor occupancy, BAY-4931 drives the equilibrium more towards repression than T0070907, analogous to expected effects of a partial agonist compared to a full agonist.

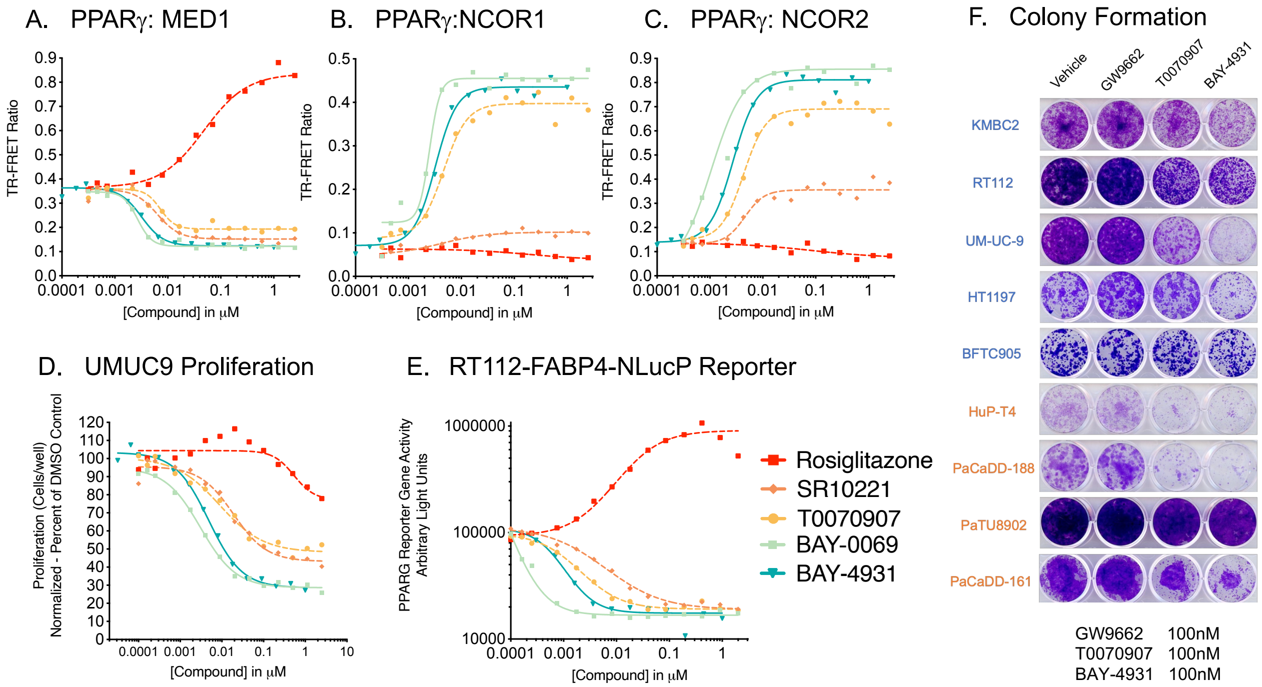

Figure 6. BAY-4931 and BAY-0069 are selective inverse-agonists of PPARγ. Biochemical TR-FRET evaluation of ligand-dependent changes in the interaction between PPARγ ligand-binding domain and interacting peptide fragments from **A.** co-activator MED1, **B.** co-repressor NCOR1, and **C.** co-repressor NCOR2. **D.** Dose-response effects of compound on the proliferation of UMUC9-H2B-GFP cells. **E.** Dose-response effects of compound on reporter activity in RT112-FABP4-NLucP **F.** Crystal violet colony formation assays comparing effects of PPARγ modulators with bladder cell lines highlighted in blue and pancreatic in orange. Samples in triplicate, representative well shown. See supporting information for additional data.

***In vivo* profiling:** Based on the strong *in vitro* effects and high selectivity of **BAY-0069** in addition to slightly improved microsomal stability over **BAY-4931** (**Table 6**), we elected to profile **BAY-0069** *in vivo*. Unfortunately, the low intrinsic solubility of **BAY-0069** prevented profiling by *i.v.* administration; however, compound exposure was assessed by oral, intraperitoneal, and subcutaneous administration, all at 100 mg/kg (**Figure 7**). Despite the high dose administered, the obtained exposure was found to be very low by all three routes with *i.p.* showing the best $AUC_{0\text{-tlast}}$ of 0.26 h*mg/L and a Cmax of 59 nM (see **Table S2**).Corrected for protein binding, the unbound Cmax of **BAY-0069** covered the IC_{50} from the FABP4-NLucP reporter gene assay but failed to exceed the antiproliferative $IC_{50}u$ from UM-UC-9 cell lines, though intraperitoneal administration came closest with a Cmax,u / IC50, u of 0.77.

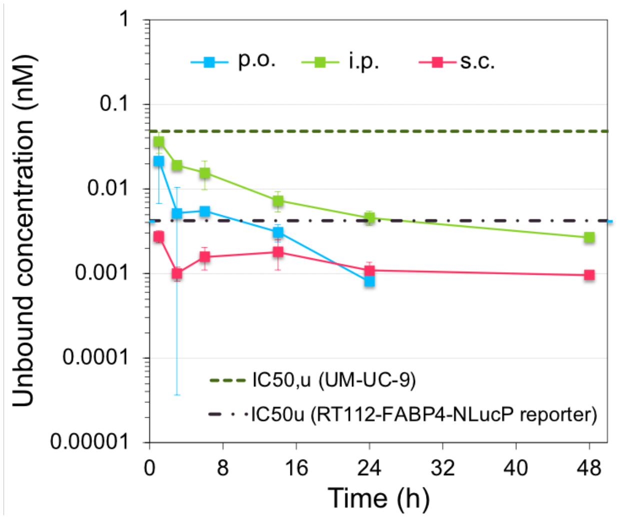

Figure 7. Exposure evaluation of BAY-0069. Unbound plasma concentrations of BAY-0069 after administration of 100 mg/kg using *p.o.*, *i.p.*, and *s.c.* administration routes versus (n=3 mice per group) *in vitro* antiproliferative IC50 (unbound) in UM-UC-9 cells as well as IC50 (unbound) in RT112-FABP4-NLucP reporter assay. See **Table S2** for detailed pharmacokinetic parameters.

Despite this limited overall exposure, the *in vivo* effect of **BAY-0069** on gene expression was examined in RT112 xenograft tumors in comparison with the non-covalent inverse-agonist SR10221. We focused on *FABP4* and *ANXA3* gene expression as a biomarker of inverse agonism, as these PPARγ target genes showed strong regulatory effects in *in vitro* mRNA expression studies[3] with *FABP4* being strongly downregulated and *ANXA3* being upregulated by inverse-agonists. *In vivo*, BAY-0069 showed modest downregulation

of *FABP4* expression, comparable with SR10221, despite more robust *in vitro* inverse agonism in NCOR2 recruitment, RT112-FABP4-NLucP luciferase repression, and UM-UC-9 proliferation (**Figure 8**). Upregulation of *ANXA3* expression was statistically significant by SR10221, but not by **BAY-0069.** Note that for **BAY-0069**, the maximum tolerable dose was reached and repeat dosing was not possible.

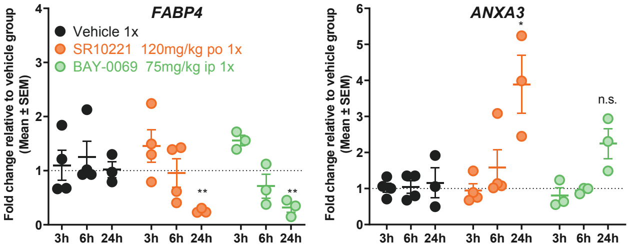

Figure 8. Pharmacodynamic regulation of PPARγ target gene expression in UM-UC-9 xenograft bearing mice. Expression of *FABP4* and *ANXA3* normalized to housekeeping gene, *PPIA*, and reported as normalized expression relative to vehicle control. Mice were treated as indicated and 3 – 4 were sacrificed at the respective time points. Statistical analysis: one-way ANOVA with Dunnett's multiple testing correction; *: $p \leq 0.05$; **: $p \leq 0.01$.

CONCLUSIONS: Herein we disclose the discovery of a halo-nitroarene series of covalent PPARγ inverse-agonists. Chemical optimization through structure-informed design led to discovery of a potent and selective PPARγ inverse-agonists. The extended benzoxazole core brought a lipophilic arene in close contact with the lipophilic surface of the co-repressor NCOR2, while the binding of the chloro-nitroarene warhead sequesters Helix-12, an essential binding surface for the co-activator MED1, deep inside the protein.

Target engagement was demonstrated in a series of biochemical assays evaluating PPARγ binding and function. Cell-based assays confirmed that the compounds were cellularly active and modulate the function of PPARγ. Antiproliferative activity was observed in a panel of cell lines selected based on their genetic dependency to *PPARG* deletion.

The combination of poor solubility, low permeability, and rapid hepatic clearance by GSH adduct formation from BAY-0069 resulted in a low exposure by all routes of administration. However, BAY-4931 and BAY-0069 show very robust *in vitro* effects in biochemical and cellular assays and provide novel tools to further the study of PPARγ structure and function.

EXPERIMENTAL SECTION

General Chemical Methods and Materials: Commercially available reagents and anhydrous solvents were used as supplied, without further purification. All air- and moisture-sensitive reactions were carried out in oven-dried (at 120 °C) glassware under an inert atmosphere of argon. A Biotage Initiator Classic microwave reactor was used for reactions conducted in a microwave oven. Reactions were monitored by TLC and UPLC analysis with a Waters Acquity UPLC-MS Single Quad system; column: Acquity UPLC BEH C18 1.7 µm, 50 × 2.1 mm; basic conditions: eluent A: H2O + 0.2 vol% aq NH3 (32%), eluent B: MeCN; gradient: 0–1.6 min 1–99% B, 1.6–2.0 min 99% B; flow: 0.8 mL/min; acidic conditions: eluent A: H2O + 0.1 vol% formic acid (99%), eluent B: MeCN; gradient: 0–1.6 min 1–99% B, 1.6–2.0 min 99% B; flow: 0.8 mL/min; temperature: 60 °C; DAD scan: 210–400 nm. Analytical TLC was carried out on aluminum-backed plates coated with Merck Kieselgel 60 F254, with visualization under UV light at 254 nm. Flash chromatography was carried out using a Biotage Isolera One system with 200–400 nm variable detector. Preparative HPLC was carried out with a Waters AutoPurification MS Single Quad system; column: Waters XBridge C18 5 µm, 100 × 30 mm; basic conditions: eluent A: H2O + 0.2 vol% aq NH3 (32%), eluent B: MeCN; gradient: 0–0.5 min 5% B, flow: 25 mL/min; 0.51–5.50 min 10–100% B, flow: 70 mL/min; 5.51–6.5 min 100% B, flow: 70 mL/min; acidic conditions: eluent A: H2O + 0.1 vol% formic acid (99%), eluent B: MeCN; gradient: 0–0.5 min 5% B, flow: 25 mL/min; 0.51–5.50 min 10–100% B, flow: 70 mL/min; 5.51–6.5 min 100% B, flow: 70 mL/min; temperature: 25 °C; DAD scan: 210–400 nm. Regioisomers were separated by preparative, chiral SFC using a Sepiatec: Prep SFC100 insrtrument; column: Chiralpak IA 5µ 250x30mm; eluent A: CO2; eluent B: methanol + 0.2 vol % aq. NH3 (32%); isocratic: 35% B; flow: 100 ml/min; temperature: 40°C; BPR: 150 bar; UV: 280 nm. Analytical, chiral SFC was carried out using an Agilent: 1260, Aurora SFC-module; column: Chiralpak IA 5µ 100x4.6mm; eluent A: CO2; eluent B: methanol + 0.2 Vol-% aq. NH3 (32%); isokratic: 35%B; flow: 4 ml/min; temperature: 37.5°C; BPR: 100 bar; UV: 280 nm. NMR spectra were recorded at rt (22 ± 1 °C), unless otherwise noted, on Bruker Avance III HD spectrometers. 1H NMR spectra were obtained at 400 or 500 MHz and referenced to the residual solvent signal (7.26 ppm for CDCl3, 2.50 ppm for DMSO-d6). 1H NMR data are reported as follows: chemical shift (δ) in ppm, multiplicity (s = singlet, d = doublet, t = triplet, q = quartet, br = broad, m = multiplet), integration, and assignment. Low-resolution mass spectra (electrospray ionization, ESI) were obtained via HPLC-MS (ESI) using a Waters Acquity UPLC system equipped with an SQ 3100 Mass Detector; column: Acquity UPLC BEH C18 1.7 µm, 50 × 2.1 mm; basic conditions: eluent A: H2O + 0.2 vol% aq NH3 (32%), eluent B: MeCN; gradient: 0–1.6 min 1–99% B, 1.6–2.0 min 99% B; flow: 0.8 mL/min; acidic conditions: eluent A: H2O + 0.1 vol% formic acid (99%), eluent B: MeCN; gradient: 0–1.6 min 1–99% B, 1.6–2.0 min 99% B; flow: 0.8 mL/min; temperature: 60 °C; DAD scan: 210–400 nm or Agilent 1290 UPLCMS 6230 TOF system; column: BEH C 18 1.7 µm, 50x2.1mm; Eluent A: water + 0.05 % formic acid (99%); Eluent B: acetonitrile + 0.05 % formic acid (99%); gradient: 0-1.7 2-90% B, 1.7-2.0 90% B; flow 1.2 ml/min; temperature: 60°C; DAD scan: 190-400 nm. . Analysis and separation of mixtures of regioisomers was performed using chiral SFC. Instrument: Agilent: 1260, Aurora SFC-Modul; column: Chiralpak IA 5µ 100x4.6mm; Eluent A: CO2; Eluent B: Methanol + 0.2 Vol-% aqueous ammonia (32%); isocratic: 35%B; flow: 4 ml/min; temperature: 37.5°C; BPR: 100bar; UV: 280 nm. Instrument: Sepiatec: Prep SFC100; column: Chiralpak IA 5µ 250x30mm; eluent A: CO2; eluent B: methanol + 0.2 vol % aqueous ammonia (32%); isocratic: 35%B; flow: 100 ml/min; temperature: 40°C; BPR: 150bar; UV: 280

nm. The purity of all target compounds was at least 95% as determined by UPLC-MS, with the exception of 6h (93%). Compound names were generated using ICS software.

2-(3-Methylphenyl)-5-nitro-1,3-benzoxazole (**6a-2**)

2-Amino-4-nitrophenol (1.80 g, 11.7 mmol) was dissolved in 90 toluene, then 1.0 eq. 3-methylbenzoyl chloride (1.5 ml, 12 mmol) was added slowly. This mixture was refluxed for 20 hours. Then 0.25 eq. p-toluenesulfonic acid (555 mg, 2.92 mmol) was added and the mixture was refluxed for 6 more hours. The reaction mixture was allowed to cool to r.t.. A dark precipitate had been formed which was collected by filtration and washed with toluene. The filtrate was evaporated under reduced pressure to give 3.54 g of the title compound (83% yield) as crude material which was used directly in the next step.

^{1}H NMR (400 MHz, DMSO-d_6) δ ppm 8.67 (d, *J* = 2.28 Hz, 1 H), 8.35 (dd, *J* = 8.87, 2.28 Hz, 1 H), 8.01 - 8.10 (m, 3 H), 7.49 - 7.58 (m, 2 H), 2.45 (s, 3 H).

2-(3-Methylphenyl)-1,3-benzoxazol-5-amine (**6a-1**)

2-(3-Methylphenyl)-5-nitro-1,3-benzoxazole (3.54 g, 13.9 mmol) was dissolved in 130 ml ethanol, then 4.0 eq. tin(II) chloride dihydrate (12.6 g, 55.6 mmol) was added. This mixture was refluxed for 2 hours. The mixture was adjusted with sodium carbonate solution (w=10%) to pH 9 and afterwards extracted 3 times with DCM, washed with water, dried over sodium sulfate, filtered, and evaporated to give 2.48 g (64% yield) crude material of the title compound which was directly used in the next step.

UPLC-MS (Waters Acquity, acidic conditions): ^{t}R. = 0.98 min. MS (ESI+): m/z = 225.2 [M+H]+.

2-Chloro-N-[2-(3-methylphenyl)-1,3-benzoxazol-5-yl]-5-nitrobenzamide (**6a**)

2-(3-Methylphenyl)-1,3-benzoxazol-5-amine (200 mg, 892 µmol), 1.2 eq. 2-chloro-5-nitrobenzoic acid (216 mg, 1.07 mmol) and 1.3 eq. p*ropanephosphonic* acid *anhydride (w=50% in DMF)* were dissolved in 12 ml DMF under argon atmosphere. The mixture was stirred at r.t. for 18h. DMF was evaporated under reduced pressure. Aqueous $NaHCO_3$-solution and ethyl acetate were added to the mixture and the layers were separated. The aqueous layer was extracted multiple times with ethyl acetate. The combined organic layers were washed with saturated NaCl-solution, dried over sodium sulfate, filtered, and evaporated to give the title compound as crude material. Purification by flash chromatography (silica gel, hexane/ ethyl acetate gradient) gave 187 mg raw material. This material was suspended in DCM/ MeOH (9:1), filtered and washed again with DCM/ MeOH (9:1) to give 31.8 mg (9% yield, 100% purity) of the title compound **6a**.

UPLC-MS (Waters Acquity, acidic conditions): ^{t}R = 1.37 min. MS (ESI+): *m/z* = 408.1 $[M+H]^{+.}$

^{1}H NMR (400 MHz, DMSO-d_6) δ ppm 10.91 (s, 1 H), 8.53 (d, *J* = 2.79 Hz, 1 H), 8.36 (dd, *J* = 8.87, 2.79 Hz, 1 H), 8.24 (d, *J* = 1.77 Hz, 1 H), 8.05 (s, 1 H), 7.98 - 8.03 (m, 1 H), 7.92 (d, *J* = 8.87 Hz, 1 H), 7.80 (d, *J* = 8.87 Hz, 1 H), 7.66 (dd, *J* = 8.87, 2.03 Hz, 1 H), 7.44 - 7.55 (m, 2 H), 2.44 (s, 3 H).

tert.-Butyl [2-(2-methylphenyl)-1,3-benzoxazol-5-yl]carbamate (**6b-2**)

A mixture of 5-bromo-2-(2-methylphenyl)-1,3-benzoxazole (1.02 g, 3.54 mmol), 1.2 eq. tert.-butyl carbamate (498 mg, 4.25 mmol), 0.1 eq. t-Bu-X-Phos (150 mg, 354 µmol), 0.03 eq. Bis(dibenzylidenaceton)palladium(0) (61 mg, 110 µmol) and 2.0 eq. sodium tert.-butylate (680 mg, 7.08 mmol) in 180 ml toluene was stirred at 60°C for 8 days. Water and DCM were added, the phases were separated, and the organic phase evaporated to dryness to give 1.28 g crude material. Purification by flash chromatography (silica gel, hexane/ ethyl acetate gradient) gave 140 mg of the impure title compound (5% yield) alongside 983 mg recovered starting material. The crude material was used without further purification in the next step.

UPLC-MS (Waters Acquity, acidic conditions): ^{t}R = 1.48 min. MS (ESI+): m/z = 325.5 [M+H]+.

2-(2-Methylphenyl)-1,3-benzoxazol-5-amine (**6b-1**)

A mixture of tert.-butyl [2-(2-methylphenyl)-1,3-benzoxazol-5-yl]carbamate (156 mg, 480 µmol) and 1.2 ml 4M HCl in dioxane (10eq., 4.8 mmol) was stirred at r.t. for 18h. The reaction mixture was evaporated to dryness. To give 155.8 mg (36 % yield) of the title compound which was directly used in the next step.

UPLC-MS (Waters Acquity, acidic conditions): ^{t}R = 0.95 min. MS (ESI+): m/z = 225.1 [M+H]+.

2-Chloro-N-[2-(2-methylphenyl)-1,3-benzoxazol-5-yl]-5-nitrobenzamide (**6b**)

A mixture of 2-chloro-5-nitrobenzoic acid (145 mg, 719 µmol) and 20 eq. thionyl chloride (1.0 ml, 14 mmol) was stirred at 80 °C for 2.5 hours. The reaction mixture was evaporated to dryness and immediately used in the next step. A mixture of 2-(2-methylphenyl)-1,3-benzoxazol-5-amine (156 mg, 695 µmol), 1 eq. 2-chloro-5-nitrobenzoyl chloride (153 mg, 695 µmol) and 5.0 eq. triethylamine (480 µl, 3.5 mmol) in 3.2 ml THF was stirred at r.t. for 4 days. The mixture was evaporated to dryness and the remaining material purified by preparative HPLC (acidic conditions) to give 21.0 mg (7% yield, 96% purity) of the title compound **6b**.

UPLC-MS (Waters Acquity, acidic conditions): ^{t}R = 1.3 min. MS (ESI+): *m/z* = 408.2 $[M+H]^+$

^1H NMR (400 MHz, DMSO-d_6) δ ppm 10.91 (s, 1 H), 8.53 (d, *J* = 2.79 Hz, 1 H), 8.37 (dd, *J* = 8.87, 2.79 Hz, 1 H), 8.27 (d, *J* = 2.03 Hz, 1 H), 8.15 (dd, *J* = 7.86, 1.27 Hz, 1 H), 7.92 (d, *J* = 8.87 Hz, 1 H), 7.81 (d, *J* = 8.87 Hz, 1 H), 7.66 (dd, *J* = 8.87, 2.03 Hz, 1 H), 7.41 - 7.56 (m, 3 H), 2.77 (s, 3 H).

5-Bromo-2-(4-ethylphenyl)-1,3-benzoxazole (**6c-3**)

4-Ethylbenzoyl chloride (18 ml, 120 mmol) was dissolved in 200 ml toluene and 2.0 eq. sodium bicarbonate dissolved in 200 ml water were added. Under vigorous stirring, 1.0 eq. 2-amino-4-bromophenol (23.4 g, 125 mmol) were added portion-wise, and the mixture stirred over night at r.t.. Ethyl acetate was added, and the mixture extracted with water three times. The organic phase was evaporated to dryness and taken up in 200 ml toluene again. 1.0 eq. p-Toluenesulfonic acid (21.4 g, 125 mmol) was added, and the reaction mixture stirred over night at 95°C. After cooling to r.t., MTBE was added, and the reaction mixture washed 3-times with aqueous sodium bicarbonate solution (w=10%). The organic phase was evaporated to dryness and the resulting crude material by flash chromatography (silica gel, hexane/ ethyl acetate gradient) to give 23.0 g (61 % yield) of the title compound.

1H NMR (400 MHz, DMSO-d6) δ ppm 8.05 - 8.28 (m, 2 H), 7.95 - 8.05 (m, 1 H), 7.68 - 7.85 (m, 1 H), 7.51 - 7.62 (m, 1 H), 7.33 - 7.50 (m, 2 H), 2.62 - 2.87 (m, 2 H), 1.23 (s, 3 H).

tert.-Butyl [2-(4-ethylphenyl)-1,3-benzoxazol-5-yl]carbamate (**6c-2**)

A mixture of 5-bromo-2-(4-ethylphenyl)-1,3-benzoxazole (22.7 g, 75.1 mmol) and 1.2 eq. tert.-butyl carbamate (13.2 g, 113 mmol) in toluene was purged with nitrogen gas. 0.1 eq. Bis(dibenzylidenaceton)palladium(0) (4.32 g, 7.5 mmol) , 0.3 eq. t-Bu-X-Phos (9.57 g, 22.5 mmol), and 3.0 eq. sodium tert.-butylate (21.7 g, 225 mmol) were added and the reaction mixture stirred at 80°C for 6 hours. The reaction mixture was evaporated to dryness, water and DCM were added and the phases separated. The combined organic phases were evaporated to dryness again and the resulting crude material purified by flash chromatography (silica gel, hexane/ ethyl acetate gradient) to give 19.6 g (77% yield) of the title compound.
1H NMR (400 MHz, DMSO-d6) δ ppm 9.40 - 9.58 (m, 1 H), 8.03 - 8.17 (m, 2 H), 7.84 - 7.97 (m, 1 H), 7.59 - 7.69 (m, 1 H), 7.38 - 7.52 (m, 3 H), 2.63 - 2.77 (m, 2 H), 1.50 (s, 9 H), 1.19 - 1.26 (m, 3 H).

2-(4-Ethylphenyl)-1,3-benzoxazol-5-amine (**6c-1**)

A mixture of tert.-butyl [2-(4-ethylphenyl)-1,3-benzoxazol-5-yl]carbamate (7.20 g, 21.3 mmol) and 53 ml HCl-solution (4 N in dioxane, 10.0 eq.) was stirred at r.t. for 16 h. The reaction was evaporated to dryness. Diluted aqueous NaOH-solution and DCM were added, the phases separated, and the aqueous phase extracted multiple time with DCM. The combined organic phases were again evaporated to dryness to give 4.90 g (97 % yield) of the title compound.UPLC-MS (Waters Acquity, acidic conditions): ^{t}R = 1.10 min. MS (ESI+): m/z = 239.3 [M+H]+.

2-Chloro-N-[2-(4-ethylphenyl)-1,3-benzoxazol-5-yl]-5-nitrobenzamide (**6c**, **BAY-4931**)

A mixture of 2-(4-ethylphenyl)-1,3-benzoxazol-5-amine (250 mg, 1.05 mmol), 1.5 eq. 2-chloro-5-nitrobenzoic acid (317 mg, 1.57 mmol), 2 eq. HATU (798 mg, 2.10 mmol), 5.0 eq. DIPEA (910 µl, 5.2 mmol) in 4.6 ml DMF was stirred at r.t. °C for 12 h. Water and saturated $NaHCO_3$-solution were added, the mixture extracted with ethyl acetate, washed with brine, dried via Na_2SO_4 and evaporated to dryness. The remaining crude material was purified by flash chromatography (silica gel, hexane/ ethyl acetate gradient) to give 190 mg (43% yield, 100% purity) of the title compound 6c.

^1H NMR (400 MHz, DMSO-d_6) δ ppm 10.90 (s, 1 H), 8.53 (d, J = 2.79 Hz, 1 H), 8.36 (dd, J = 8.87, 2.79 Hz, 1 H), 8.22 (d, J = 1.77 Hz, 1 H), 8.09 - 8.16 (m, 2 H), 7.92 (d, J = 8.87 Hz, 1 H), 7.79 (d, J = 8.87 Hz, 1 H), 7.65 (dd, J = 8.87, 2.03 Hz, 1 H), 7.47 (d, J = 8.62 Hz, 2 H), 2.72 (q, J = 7.60 Hz, 2 H), 1.24 (t, J = 7.60 Hz, 3 H).

UPLC-MS (Waters Acquity, acidic conditions): ^{t}R = 1.42 min. MS (ESI+): m/z = 422.2 $[M+H]^+$.

Refer to Figure S6 for HPLC trace of **6c** (**BAY-4931**)

5-Bromo-2-(3-chlorophenyl)-1,3-benzoxazole (**6d-3**)

A mixture of 2-amino-4-bromophenol (2.00 g, 10.6 mmol), 1.2 eq. 3-chlorobenzoyl chloride (2.05 g, 12 mmol) in 40 ml toluene was stirred at 80 °C for 3 days. 1.0 eq. p-toluenesulfonic acid (1.83 g, 10.6 mmol) were added, and the reaction mixture stirred another 3 days at 80°C. The mixture was evaporated to dryness, water and ethyl acetate added and the phases separated. The aqueous phase was extracted multiple times with ethyl acetate, the combined organic phases washed with diluted aqueous NaOH-solution, dried with Na_2SO_4 and evaporated to dryness again. The remaining crude material was purified by flash chromatography (silica gel, hexane/ ethyl acetate gradient) to give 3.0 g (91% yield) of the title compound.

UPLC-MS (Waters Acquity, acidic conditions): ^{t}R = 1.61 min. MS (ESI+): m/z = 310.0 [M+H]+.

tert.-Butyl [2-(3-chlorophenyl)-1,3-benzoxazol-5-yl]carbamate (**6d-2**)

A mixture of 5-bromo-2-(3-chlorophenyl)-1,3-benzoxazole (3.00 g, 9.72 mmol), 2.0 eq. ter.t.-butyl carbamate (2.28 g, 19.4 mmol), 0.1 eq. Bis(dibenzylidenaceton)palladium(0) (0.56 g, 0.97 mmol), 0.1 eq. t-Bu-X-Phos (413 mg, 0.97 mmol) and 2.0 eq. sodium ter.t.-butylate (1.87 g, 19.4 mmol) in 220 ml toluene was stirred at 90°C for 6 days. The reaction mixture was evaporated to dryness, water and ethyl actetate were added and the phases separated. The aqueous layer was extracted with ethyl acetate. The combined organic phases were washed with saturated NaCl-solution, dried over Na_2SO_4 and evaporated to dryness. The resulting crude material was purified by flash chromatography (silica gel, hexane/ ethyl acetate gradient) to give 1.66 g (50% yield) of the title compound.

UPLC-MS (Waters Acquity, acidic conditions): ^{t}R = 1.51 min. MS (ESI+): m/z = 345.2 [M+H]+.

2-(3-Chlorophenyl)-1,3-benzoxazol-5-amine (**6d-1**)

A mixture of tert.-butyl [2-(3-chlorophenyl)-1,3-benzoxazol-5-yl]carbamate (1.66 g, 4.81 mmol) and 30 ml HCl-solution (4 N in dioxane, 25.0 eq.) was stirred at r.t. for 24 h. The reaction was evaporated to dryness to give the title compound which was used in the next step without further purification.

UPLC-MS (Waters Acquity, acidic conditions): ^{t}R = 1.06 min. MS (ESI+): m/z = 246.0 [M+H]+.

2-Chloro-N-[2-(3-chlorophenyl)-1,3-benzoxazol-5-yl]-5-nitrobenzamide (**6d**)

A mixture of 2-(3-chlorophenyl)-1,3-benzoxazol-5-amine (100 mg, 409 µmol), 1.4 eq. 2-chloro-5-nitrobenzoic acid (115 mg, 572 µmol), 1.3 eq. HATU (202 mg, 531 µmol), 3.0 eq. DIPEA (210 µl, 1.2 mmol) in 1.8 ml DMF was stirred at r.t. until complete conversion. Water and ethyl acetate were added, and the phases separated. The aqueous phase was extracted with multiple times with ethyl acetate, washed with saturated NaCl-solution, dried with Na_2SO_4 and evaporated to dryness. The remaining crude material was purified by preparative HPLC (acidic conditions) to give 29 mg (16% yield, 98% purity) of the title compound **6d**.
UPLC-MS (Waters Acquity, acidic conditions): ^{t}R = 1.37 min. MS (ESI+): *m*/*z* = 428.0 $[M+H]^+$.

^{1}H NMR (400 MHz, DMSO-d_6) δ ppm 10.94 (s, 1 H), 8.53 (d, *J* = 2.79 Hz, 1 H), 8.37 (dd, *J* = 8.87, 2.79 Hz, 1 H), 8.27 (d, *J* = 1.77 Hz, 1 H), 8.15 - 8.21 (m, 2 H), 7.92 (d, *J* = 8.87 Hz, 1 H), 7.83 (d, *J* = 8.87 Hz, 1 H), 7.64 - 7.76 (m, 3 H).

N-[2-(2-Bromophenyl)-1,3-benzoxazol-5-yl]-2-chloro-5-nitrobenzamide (**6e**)

UPLC-MS (Waters Acquity, acidic conditions): R_t = 1.34 min. MS (ESI+): m/z = 474.2 [M+H]+, 100% purity.

^{1}H NMR (400 MHz, DMSO-d_6) δ ppm 10.95 (s, 1 H), 8.54 (d, *J* = 2.53 Hz, 1 H), 8.37 (dd, *J* = 8.62, 2.79 Hz, 1 H), 8.30 (d, *J* = 1.77 Hz, 1 H), 8.10 (dd, *J* = 7.73, 1.65 Hz, 1 H), 7.89 - 7.95 (m, 2 H), 7.85 (d, *J* = 8.87 Hz, 1 H), 7.70 (dd, *J* = 8.87, 2.03 Hz, 1 H), 7.54 - 7.66 (m, 2 H).

5-Bromo-2-(3-fluoro-4-methoxyphenyl)-1,3-benzoxazole (**6f-3**)

A mixture of 3-fluoro-4-methoxybenzoic acid (2.17 g, 12.8 mmol) and 10 eq. $SOCl_2$ (9.3 ml, 130 mmol) was stirred at 60 °C for 3 h. The mixture was evaporated to dryness and the resulting acid chloride used directly in the next step. A mixture of 2-amino-4-bromophenol (2.39 g, 12.7 mmol), 1.0 eq. 3-fluoro-4-methoxybenzoyl chloride (2.40 g, 12.7 mmol) in 100 ml toluene was stirred at 80 °C for 16 h. 0.6 eq. p-toluenesulfonic acid (1.31 g, 7.64 mmol) were added, and the reaction mixture stirred overnight at 80°C. The mixture was evaporated to dryness, and the remaining crude material was purified by flash chromatography (silica gel, hexane/ ethyl acetate gradient) to give 2.13 g (52% yield) of the title compound.

UPLC-MS (Waters Acquity, acidic conditions): R_t = 1.48 min. MS (ESI+): m/z = 324.0 [M+H]+.

tert.-Butyl [2-(3-fluoro-4-methoxyphenyl)-1,3-benzoxazol-5-yl]carbamate (**6f-2**)

A mixture of 5-bromo-2-(3-fluoro-4-methoxyphenyl)-1,3-benzoxazole (4.00 g, 12.4 mmol), 2.0 eq. tert.-butyl carbamate (2.91 g, 24.8 mmol), 0.1 eq. Bis(dibenzylidenaceton)palladium(0) (0.71 g, 1.24 mmol), 0.1 eq. t-Bu-X-Phos (527 mg, 1.24 mmol) and 2.0 eq. sodium ter.t.-butylate (2.39 g, 24.8 mmol) in 280 ml toluene was stirred at 90°C for 6 days. The reaction mixture was evaporated to dryness, water and ethyl actetate were added and the phases separated. The aqueous layer was extracted with ethyl acetate. The combined organic phases were washed with saturated NaCl-solution, dried over Na_2SO_4 and evaporated to dryness. The resulting crude material was purified by flash chromatography (silica gel, hexane/ ethyl acetate gradient) to give 2.14 g (48% yield) of the title compound.

UPLC-MS (Waters Acquity, acidic conditions): R_t = 1.37 min. MS (ESI+): m/z = 359.4 [M+H]+.

2-(3-Fluoro-4-methoxyphenyl)-1,3-benzoxazol-5-amine (**6f-1**)

A mixture of tert.-butyl [2-(3-fluoro-4-methoxyphenyl)-1,3-benzoxazol-5-yl]carbamate (611 mg, 1.70 mmol) and 15 ml HCl-solution (4 N in dioxane, 35.0 eq.) was stirred at r.t.

until complete conversion. The reaction was evaporated to dryness. 2M aqueous NaOH-solution and DCM were added, the phases separated, and the aqueous phase extracted multiple time with DCM. The combined organic phases were again evaporated to dryness to give 464 mg of the title compound.

UPLC-MS (Waters Acquity, acidic conditions): ^{t}R = 0.89 min. MS (ESI+): *m/z* = 259.1 $[M+H]^+$.

^{1}H NMR (400 MHz, DMSO-d_6) δ ppm 7.91 - 7.97 (m, 1 H), 7.88 (dd, *J* = 12.04, 2.15 Hz, 1 H), 7.34 - 7.41 (m, 2 H), 6.84 (d, *J* = 2.03 Hz, 1 H), 6.65 (dd, *J* = 8.62, 2.28 Hz, 1 H), 5.11 (s, 2 H), 3.94 (s, 3 H).

2-Chloro-N-[2-(3-fluoro-4-methoxyphenyl)-1,3-benzoxazol-5-yl]-5-nitrobenzamide (**6f**)

A mixture of 2-(3-fluoro-4-methoxyphenyl)-1,3-benzoxazol-5-amine (92.0 mg, 356 µmol), 1.3 eq. 2-chloro-5-nitrobenzoic acid (93.3 mg, 463 µmol), 2.0 eq. PyBrop (332 mg, 712 µmol), 5.0 eq. DIPEA (310 µl, 1.8 mmol) in 2.9 ml DMF was stirred at r.t. for 3d. Water and DCM were added, the phases separated, and the aqueous phase extracted multiple times with DCM. The combined organic phases were evaporated to dryness. The remaining crude material was purified by preparative HPLC (basic conditions) to give 50 mg (32% yield, 96% purity) of the title compound **6f**.

UPLC-MS (Waters Acquity, basic conditions): ^{t}R = 1.24 min. MS (ESI+): *m/z* = 442.0 $[M+H]^+$.

^{1}H NMR (400 MHz, DMSO-d_6) δ ppm 10.90 (s, 1 H), 8.53 (d, *J* = 2.79 Hz, 1 H), 8.36 (dd, *J* = 8.62, 2.79 Hz, 1 H), 8.21 (d, *J* = 1.77 Hz, 1 H), 7.95 - 8.05 (m, 2 H), 7.92 (d, *J* = 8.87 Hz, 1 H), 7.78 (d, *J* = 8.87 Hz, 1 H), 7.65 (dd, *J* = 8.87, 2.03 Hz, 1 H), 7.42 (t, *J* = 8.74 Hz, 1 H), 3.96 (s, 3 H).

5-Bromo-2-(5-methylpyridin-3-yl)-1,3-benzoxazole (**6g-3**)

A mixture of 5-methylpyridine-3-carboxylic acid (3.91 g, 28.5 mmol) and 10 eq. (21 ml, 290 mmol) was stirred at 60 °C for 2 days. The mixture was evaporated to dryness and the resulting acid chloride used directly in the next step. A mixture of 2-amino-4-bromophenol (5.30 g, 28.2 mmol), 1.0 eq. 5-methylpyridine-3-carbonyl chloride (4.39 g, 28.2 mmol) in 110 ml toluene was stirred at 80 °C for 6 days. 0.6 eq. p-toluenesulfonic acid (2.91 g, 16.9 mmol) were added, and the reaction mixture stirred for 3 days at 80°C. The mixture was evaporated to dryness. Water, 31 mL 2N NaOH-solution and DCM were added and the phases separated. The organic phase was evaporated to dryness and the remaining crude material was purified by flash chromatography (silica gel, hexane/ ethyl acetate gradient) to give 200 mg (2.5% yield) of the title compound alongside 2.5 g recovered aminophenol starting material.

UPLC-MS (Waters Acquity, acidic conditions): ^{t}R = 1.27 min. MS (ESI+): m/z = 291.0 [M+H]+.

tert.-Butyl [2-(5-methylpyridin-3-yl)-1,3-benzoxazol-5-yl]carbamate (**6g-2**)

A mixture of 5-bromo-2-(5-methylpyridin-3-yl)-1,3-benzoxazole (550 mg, 1.90 mmol), 1.2 eq. tert.-butyl carbamate (267 mg, 2.28 mmol), 0.03 eq. Bis(dibenzylidenaceton)palladium(0) (33 mg, 57 µmol), 0.1 eq. t-Bu-X-Phos (80.8 mg, 190 µmol) and 2.0 eq. sodium ter.t.-butylate ((366 mg, 3.80 mmol) in 94 ml toluene was stirred at 90°C for 4 hours. The reaction mixture was evaporated to dryness, water and DCM were added and the phases separated. The aqueous layer was extracted multiple times with DCM. The combined organic phases were evaporated to dryness. The resulting crude material was purified by flash chromatography (silica gel, hexane/ ethyl acetate gradient) to give 500 mg (81% yield) of the title compound.

UPLC-MS (Waters Acquity, acidic conditions): ^{t}R = 1.20 min. MS (ESI+): m/z = 326.5 [M+H]+.

2-(5-Methylpyridin-3-yl)-1,3-benzoxazol-5-amine (**6g-1**)

A mixture tert.-butyl [2-(5-methylpyridin-3-yl)-1,3-benzoxazol-5-yl]carbamate (500 mg, 1.54 mmol) and 3.8 ml HCl-solution (4 N in dioxane, 10.0 eq.) was stirred at r.t. until complete conversion. Water and DCM were added, and the phases separated. The aqueous phase extracted multiple time with DCM. The combined organic phases were again evaporated to dryness to give 464 mg of the title compound to give 300 mg (87% yield) of the title compound.

UPLC-MS (Waters Acquity, acidic conditions): ^{t}R = 0.66 min. MS (ESI+): m/z = 226.2 [M+H]+.

2-Chloro-N-[2-(5-methylpyridin-3-yl)-1,3-benzoxazol-5-yl]-5-nitrobenzamide (**6g**)

A mixture of 2-(5-methylpyridin-3-yl)-1,3-benzoxazol-5-amine (41.0 mg, 182 µmol), 1.3 eq. 2-chloro-5-nitrobenzoic acid (47.7 mg, 237 µmol), 2.0 eq. PyBrop (170 mg, 364 µmol), 4.0 eq. DIPEA (130 µl, 730 µmol) and 0.05 eq. 4-dimethylaminopyridine (1.11 mg, 9.10 µmol) in 0.8 ml DMF was stirred at r.t. until complete conversion. The reaction mixture was evaporated to dryness. The remaining crude material was purified by flash chromatography (silica gel, hexane/ ethyl actetate gradient) to give 30 mg (36% yield, 100% purity) of the title compound **6g**.

LC-MS (Agilent, acidic conditions): ^{t}R = 1.05 min. MS (ESI+): *m/z* = 409.1 $[M+H]^+$.

^{1}H NMR (400 MHz, DMSO-d_6) δ ppm 10.94 (s, 1 H), 9.17 (d, *J* = 1.77 Hz, 1 H), 8.66 (d, *J* = 1.27 Hz, 1 H), 8.54 (d, *J* =

2.79 Hz, 1 H), 8.33 - 8.42 (m, 2 H), 8.28 (d, *J* = 1.77 Hz, 1 H), 7.92 (d, *J* = 8.87 Hz, 1 H), 7.84 (d, *J* = 8.87 Hz, 1 H), 7.69 (dd, *J* = 8.87, 2.03 Hz, 1 H), 2.45 (s, 3 H).

N-(5-Bromo-2-hydroxyphenyl)-6-ethylpyridine-3-carboxamide (**6h-4**)

A mixture of 2-amino-4-bromophenol (2.00 g, 10.6 mmol), 1.2 eq. 6-ethylpyridine-3-carboxylic acid (1.93 g, 12.8 mmol), 1.5 eq. HATU (6.07 g, 16.0 mmol), 5.0 eq. TEA (7.4 ml, 53 mmol) in 50 ml DMF was stirred at r.t. for 2 h. DMF was evaporated under reduced pressure. Water and DCM were added, and the phases separated.
The aqueous layer was extracted multiple times with DCM. The combined organic phases were evaporated to dryness and the remaining crude material purified by flash chromatography (silica gel, hexane/ ethyl acetate gradient) to give 1.80 g (53% yield) of the title compound.

LC-MS (Agilent, acidic conditions): ^tR = 0.86 min. MS (ESI+): m/z = 321.1 $[M+H]^+$.

^1H NMR (400 MHz, DMSO-d_6) δ ppm 10.13 (s, 1 H), 9.70 (s, 1 H), 9.01 (d, *J* = 1.77 Hz, 1 H), 8.21 (dd, *J* = 8.11, 2.28 Hz, 1 H), 7.88 (d, *J* = 2.28 Hz, 1 H), 7.42 (d, *J* = 8.11 Hz, 1 H), 7.21 (dd, *J* = 8.62, 2.53 Hz, 1 H), 6.88 (d, *J* = 8.62 Hz, 1 H), 2.83 (q, *J* = 7.60 Hz, 2 H), 1.25 (t, *J* = 7.60 Hz, 3 H).

5-Bromo-2-(6-ethylpyridin-3-yl)-1,3-benzoxazole (**6h-3**)

A mixture of N-(5-bromo-2-hydroxyphenyl)-6-ethylpyridine-3-carboxamide (1.8 g, 5.60 mmol) and 15 ml polyphosphoric acid was stirred at 200 °C for 2 h. After cooling to room temperature, ice water and DCM were added, and the phases separated. The aqueous layer was extracted multiple times with DCM. The combined organic phases were evaporated to dryness to give 1.40 g (82% yield) of the title compound which was used without further purification in the next step.

LC-MS (Agilent, acidic conditions): ^tR = 1.27 min. MS (ESI+): m/z = 303.1 $[M+H]^+$.

^1H NMR (400 MHz, DMSO-*d_6*) δ ppm 9.24 (dd, *J* = 2.28, 0.76 Hz, 1 H), 8.43 (dd, *J* = 8.11, 2.28 Hz, 1 H), 8.08 (d, *J* = 2.03 Hz, 1 H), 7.81 (d, *J* = 8.62 Hz, 1 H), 7.61 (dd, *J* = 8.74, 1.90 Hz, 1 H), 7.53 (d, *J* = 7.86 Hz, 1 H), 2.87 (q, *J* = 7.60 Hz, 2 H), 1.28 (t, *J* = 7.60 Hz, 3 H).

tert.-Butyl [2-(6-ethylpyridin-3-yl)-1,3-benzoxazol-5-yl]carbamate (**6h-2**)

A mixture of 5-bromo-2-(6-ethylpyridin-3-yl)-1,3-benzoxazole (1400 mg, 4.62 mmol), 1.5 eq. tert.-butyl carbamate (812 mg, 6.92 mmol), 0.1 eq. Bis(dibenzylidenaceton)palladium(0) (266 mg, 462 µmol), 0.3 eq. t-Bu-X-Phos (588 mg, 1.39 mmol) and 3.0 eq. sodium ter.t.-butylate (1.33 g, 13.86 mmol) in 38 ml toluene was stirred at 90°C for 3 hours. The reaction mixture was evaporated to dryness, water and DCM were added and the phases separated. The aqueous layer was extracted multiple times with DCM. The combined organic phases were evaporated to dryness. The resulting crude material was purified by flash chromatography (silica gel, hexane/ ethyl acetate gradient) to give 1.30 g (83% yield) of the title compound.

UPLC-MS (Waters Acquity, acidic conditions): ^tR = 1.27 min. MS (ESI+): m/z = 340.6 [M+H]+.

2-(6-Ethylpyridin-3-yl)-1,3-benzoxazol-5-amine (**6h-1**)

A mixture tert.-butyl [2-(6-ethylpyridin-3-yl)-1,3-benzoxazol-5-yl]carbamate (1.30 g, 3.83 mmol) and 9.6 ml HCl-solution (4 N in dioxane, 10.0 eq.) was stirred at r.t. until complete conversion. Water, saturated $NaHCO_3$-solution and DCM were added, and the phases separated. The aqueous phase extracted multiple time with DCM. The combined organic phases were again evaporated to dryness to give 464 mg of the title compound to give the title compound which was used without further purification in the next step.

UPLC-MS (Waters Acquity, acidic conditions): ^tR = 0.76 min. MS (ESI+): m/z = 240.3 [M+H]+.

2-Chloro-N-[2-(6-ethylpyridin-3-yl)-1,3-benzoxazol-5-yl]-5-nitrobenzamide (**6h**)

A mixture of 2-(6-ethylpyridin-3-yl)-1,3-benzoxazol-5-amine (88.0 mg, 368 µmol), 1.1 eq. 2-chloro-5-nitrobenzoic acid (81.5 mg, 405 µmol), 5.0 eq. TEA (260 µl, 1.8 mmol), 2.0 eq. HATU (280 mg, 0.74 mmol) in 4.4 ml DMF was stirred at r.t. until complete conversion. The reaction mixture was evaporated to dryness and purified by preparative HPLC (acidic conditions) to give 50.0 mg (29% yield, 93% purity) of the title compound **6h**.

LC-MS (Agilent, acidic conditions): ^tR = 1.14 min. MS (ESI+): *m/z* = 423.1 $[M+H]^+$.

^1H NMR (400 MHz, DMSO-*d_6*) δ ppm 10.93 (s, 1 H), 9.23 - 9.29 (m, 1 H), 8.53 (d, *J* = 2.79 Hz, 1 H), 8.45 (dd, *J* = 8.24, 2.41 Hz, 1 H), 8.36 (dd, *J* = 8.87, 2.79 Hz, 1 H), 8.26 (d, *J* = 1.77 Hz, 1 H), 7.92 (d, *J* = 8.62 Hz, 1 H), 7.83 (d, *J* = 8.87 Hz, 1 H), 7.69 (dd, *J* = 8.87, 2.03 Hz, 1 H), 7.54 (d, *J* = 8.36 Hz, 1 H), 2.88 (q, *J* = 7.60 Hz, 2 H), 1.29 (t, *J* = 7.60 Hz, 3 H).

2-Chloro-N-[2-(4-methylphenyl)-1,3-benzoxazol-6-yl]-5-nitrobenzamide (**8a**)

A mixture of 2-(4-methylphenyl)-1,3-benzoxazol-6-amine (80.0 mg, 357 µmol), 1.3 eq. 2-chloro-5-nitrobenzoic acid (93.5 mg, 464 µmol), 2.0 eq. PyBrop (333 mg, 713 µmol) and 5.0 eq. DIPEA (310 µl, 1.8 mmol) in 2.7 ml DMF was stirred at r.t. until complete conversion. The reaction mixture was evaporated to dryness. The remaining crude material was purified by preparative HPLC (acidic conditions) to give 38 mg (25% yield, 96% purity) of the title compound 8**a**.

UPLC-MS (Waters Acquity, acidic conditions): ^tR = 1.38 min. MS (ESI+): *m/z* = 407.9 $[M+H]^+$.

^1H NMR (400 MHz, DMSO-d_6) δ ppm 11.02 (s, 1 H), 8.53 (d, *J* = 2.79 Hz, 1 H), 8.37 (dd, *J* = 8.87, 2.79 Hz, 1 H), 8.32 (d, *J* = 1.77 Hz, 1 H), 8.10 (d, *J* = 8.11 Hz, 2 H), 7.92 (d, *J* = 8.87 Hz, 1 H), 7.79 (d, *J* = 8.87 Hz, 1 H), 7.56 (dd, *J* = 8.74, 1.90 Hz, 1 H), 7.44 (d, *J* = 8.11 Hz, 2 H), 2.42 (s, 3 H).

5-Bromo-2-(4-ethylphenyl)-1H-benzimidazole (**8b-4** and **8c-4**)

A mixture of 4-bromobenzene-1,2-diamine (5.44 g, 29.1 mmol), 1.1 eq. 4-ethylbenzaldehyde (4.29 g, 32.0 mmol), 1.2 eq. oxone (10.7 g, 34.9 mmol) in 2.0 ml water and 100 ml DMF was stirred at r.t. for 17 h. A precipitate had been formed and was filtered of to give 8.0 g (91% yield) of the title compound which was used without further purification in the next step.

UPLC-MS (Waters Acquity acidic conditions): ^tR = 1.11 min. MS (ESI+): m/z = 302.4 [M+H]+.

5-Bromo-2-(4-ethylphenyl)-1-methyl-1H-benzimidazole & 6-Bromo-2-(4-ethylphenyl)-1-methyl-1H-benzimidazole (**8b-3** and **8c-3**)

A mixture of 5-bromo-2-(4-ethylphenyl)-1H-benzimidazole (2.00 g, 6.64 mmol), 1.1 eq. iodomethane (455 µl, 7.3 mmol, 1.2 eq. Cs_2CO_3 (2.60 g, 7.97 mmol) in 30 ml acetonitrile was stirred at r.t. until complete conversion. The reaction mixture was filtrated off and evaporated to dryness. The remaining crude material was purified by flash chromatography (silical gel, hexane, ethyl acetate gradient) to give 1.00 g (48% yield) of the title compound as mixture of two regioisomers which were not separated at this step.

UPLC-MS (Waters Acquity, acidic conditions): ^tR = 1.20 & 1.21 min. MS (ESI+): m/z = 316.4 [M+H]+.

^1H NMR (400 MHz, DMSO-d_6) δ ppm 7.92 (d, *J* = 2.03 Hz, 1 H), 7.87 (d, *J* = 1.52 Hz, 1 H), 7.75 - 7.80 (m, 4 H), 7.59 – 7.63 (m, 2 H), 7.40 – 7.44 (m, 5 H), 7.37 (dd, *J* = 8.49, 1.90 Hz, 1 H), 3.87 - 3.90 (m, 1 H), 3.87 (2 x s, 2 x 3 H), 2.71 (d, *J* = 7.35 Hz, 2 x 2 H), 1.25 (t, *J* = 7.60 Hz, 2 x 3 H).

tert.-Butyl [2-(4-ethylphenyl)-1-methyl-1H-benzimidazol-5-yl]carbamate & tert.-Butyl [2-(4-ethylphenyl)-1-methyl-1H-benzimidazol-6-yl]carbamate (**8b–2** and **8c-2**)

A mixture of 5-bromo-2-(4-ethylphenyl)-1-methyl-1H-benzimidazole and 6-bromo-2-(4-ethylphenyl)-1-methyl-1H-benzimidazole (700 mg, 2.22 mmol), 1.2 eq. tert.-butyl carbamate (312 mg, 2.66 mmol), 0.03 eq. Bis(dibenzylidenaceton)palladium(0) (38 mg, 67 µmol), 0.1 eq. t-Bu-X-Phos (94.3 mg, 222 µmol) and 2.0 eq. sodium ter.t.-butylate (427 mg, 4.44 mmol) in 110 ml toluene was stirred at 60°C for 3 days. The reaction mixture was evaporated to dryness and the resulting crude material was purified by flash chromatography (silica gel, hexane/ ethyl acetate gradient) to give 260 g (33% yield) of the title compound as mixture of regioisomers which were not separated at this step.

UPLC-MS (Waters Acquity, acidic conditions): ^tR = 1.01 & 1.04 min. MS (ESI+): m/z = 352.7 [M+H]+.

2-(4-Ethylphenyl)-1-methyl-1H-benzimidazol-5-amine & 2-(4-Ethylphenyl)-1-methyl-1H-benzimidazol-6-amine (**8b-1** and **8c-1**)

A mixture tert.-butyl [2-(4-ethylphenyl)-1-methyl-1H-benzimidazol-5-yl]carbamate & tert.-butyl [2-(4-ethylphenyl)-1-methyl-1H-benzimidazol-6-yl]carbamate (260 mg, 740 µmol) and 1.8 ml HCl-solution (4 N in dioxane, 10.0 eq.) was stirred at r.t. until complete conversion. The reaction mixture was evaporated to dryness to give 190 mg of the title compound to give the title compound as mixture of regioisomers which were not separated at this step.

UPLC-MS (Waters Acquity, acidic conditions): ^tR = 0.75 & 0.77 min. MS (ESI+): m/z = 252.2 [M+H]+.

2-Chloro-N-[2-(4-ethylphenyl)-1-methyl-1H-benzimidazol-5-yl]-5-nitrobenzamide (**8c**)

A mixture of 2-chloro-5-nitrobenzoic acid (200 mg, 992 µmol) and 20 eq. thionyl chloride (1.4 ml, 20 mmol) was stirred at 80 °C for 2 hours. The mixture was evaporated to dryness and used immediately in the next step. A mixture of 2-(4-ethylphenyl)-1-methyl-1H-benzimidazol-5-amine and 2-(4-ethylphenyl)-1-methyl-1H-benzimidazol-6-amine (190 mg, 756 µmol), 1.3 eq. 2-chloro-5-nitrobenzoyl chloride (216 mg, 983 µmol), 3.0 eq. TEA (320 µl, 2.3 mmol) in 3.5 ml THF was stirred at r.t. until complete conversion. DCM and water were added, and the phases separated. The aqueous phase was extracted multiple times with DCM. The combined organic phases were evaporated to dryness and the remaining crude material purified by flash chromatography to give 290 mg of a mixture of regioisomers which were separated by chiral preparative SFC.

Separation of regioisomers by SFC gave 90 mg (27% yield, 99% purity) **8c**. Regiochemistry was unambiguously assigned using NOESY (**Figure S7**).

Analytical SFC: ^tR = 4.55 min.

LC-MS (Agilent, acidic conditions): ^tR = 0.90 min. MS (ESI+): *m/z* = 435.2 $[M+H]^+$.

^1H NMR (500 MHz, DMSO-d_6) δ ppm 10.71 (s, 1 H), 8.50 (d, *J* = 2.86 Hz, 1 H), 8.33 - 8.38 (m, 1 H), 8.11 (d, *J* = 1.59 Hz, 1 H), 7.91 (d, *J* = 8.90 Hz, 1 H), 7.79 (d, *J* = 8.27 Hz, 2 H), 7.54 - 7.62 (m, 2 H), 7.43 (d, *J* = 8.27 Hz, 2 H), 3.89 (s, 3 H), 2.72 (q, *J* = 7.63 Hz, 2 H), 1.26 (t, *J* = 7.63 Hz, 3 H).

2-Chloro-N-[2-(4-ethylphenyl)-1-methyl-1H-benzimidazol-6-yl]-5-nitrobenzamide (**8b**)

Separation of regioisomers by SFC gave 128 mg (39% yield, 98% purity) **8b**. Regiochemistry was unambiguously assigned using NOESY (**Figure S8**).

Analytical SFC: ^tR = 3.00 min.

LC-MS (Agilent, acidic conditions): ^tR = 0.9 min. MS (ESI+): *m/z* = 435.2 $[M+H]^+$.

^1H NMR (500 MHz, DMSO-d_6) δ ppm 10.83 (s, 1 H), 8.49 (d, *J* = 2.86 Hz, 1 H), 8.36 (dd, *J* = 8.90, 2.86 Hz, 1 H), 8.18 (d, *J* = 1.91 Hz, 1 H), 7.91 (d, *J* = 8.58 Hz, 1 H), 7.75 - 7.79 (m, 2 H), 7.66 (d, *J* = 8.58 Hz, 1 H), 7.42 (d, *J* = 8.27 Hz, 2 H), 7.37 (dd, *J* = 8.58, 1.91 Hz, 1 H), 3.86 (s, 3 H), 2.72 (q, *J* = 7.63 Hz, 2 H), 1.26 (t, *J* = 7.63 Hz, 3 H).

2-Phenylimidazo[1,2-a]pyridin-6-amine (**8d-1**)

A mixture of 2-(2-chlorophenyl)-6-nitroimidazo[1,2-a]pyridine (5.38 g, 19.7 mmol) and Pd/charcoal in 120 ml MeOH was stirred under H2 atmosphere at r.t. for 6 hours. The reaction mixture was filtrated over celite and evaporated to dryness to give 2.0 g (49% yield) of the title compound which was used without further purification in the next step.

UPLC-MS (Waters Acquity, acidic conditions): ^tR = 0.59 min. MS (ESI+): m/z = 210.4 [M+H]+.

2-Chloro-5-nitro-N-(2-phenylimidazo[1,2-a]pyridin-6-yl)benzamide (**8d**)

A mixture of 2-phenylimidazo[1,2-a]pyridin-6-amine (100 mg, 478 µmol), 1.3 eq. 2-chloro-5-nitrobenzoic acid (125 mg, 621 µmol), 2.0 eq. PyBrop (446 mg, 956 µmol) and 5.0 eq. DIPEA (420 µl, 2.4 mmol) in 3.9 ml DMF was stirred at r.t. until complete conversion. Water and DCM were added, and the phases separated. The aqueous phase was extracted multiple times with DCM. The combined organic phases were evaporated to dryness. The remaining crude material was purified by preparative HPLC (acidic conditions) to give 20 mg 11% yield, 95% purity) of the title compound **8d**.

LC-MS (Agilent, acidic conditions): ^tR = 0.78 min. MS (ESI+): *m/z* = 393.1 $[M+H]^+$.

^1H NMR (400 MHz, DMSO-d_6) δ ppm 10.91 (s, 1 H), 9.34 (dd, *J* = 2.03, 1.01 Hz, 1 H), 8.54 - 8.58 (m, 2 H), 8.37 (dd, *J* = 8.87, 2.79 Hz, 1 H), 7.89 - 7.98 (m, 3 H), 7.63 (d, *J* = 9.63 Hz, 1 H), 7.41 - 7.48 (m, 2 H), 7.25 - 7.35 (m, 2 H).

2-Chloro-N-[2-(4-methylphenyl)-2H-benzotriazol-5-yl]-5-nitrobenzamide (**8e**)

A mixture of 2-(4-methylphenyl)-2H-benzotriazol-5-amine (353 mg, 1.57 mmol), 1.5 eq. 2-chloro-5-nitrobenzoic acid (476 mg, 2.36 mmol, 2.0 eq. HATU (1.20 g, 3.15 mmol) and 5.0 eq. DIPEA (1.4 ml, 7.9 mmol) in 6.9 ml DMF was stirred at r.t. until complete conversion. The remaining crude material was purified by flash chromatography (silica gel, hexane/ ethyl acetate gradient) to give 227 mg (36 % yield, 100% purity) of the title compound **8e**.

UPLC-MS (acidic conditions): ^tR = 1.40 min. MS (ESI+): m/z = 408.3 [M+H]+

^1H NMR (400 MHz, DMSO-d_6) δ ppm 11.06 (s, 1 H), 8.57 (d, *J* = 2.53 Hz, 2 H), 8.38 (dd, *J* = 8.87, 2.79 Hz, 1 H), 8.20 (d, *J* = 8.62 Hz, 2 H), 8.05 (d, *J* = 8.62 Hz, 1 H), 7.93 (d, *J* = 8.87 Hz, 1 H), 7.61 (dd, *J* = 9.25, 1.90 Hz, 1 H), 7.46 (d, *J* = 8.36 Hz, 2 H), 2.42 (s, 3 H).

2-Chloro-N-[2-(4-ethylphenyl)-1,3-benzoxazol-5-yl]-5-nitrobenzene-1-sulfonamide (**8f**)

A mixture of 2-(4-ethylphenyl)-1,3-benzoxazol-5-amine (98.0 mg, 411 µmol), 1.0 eq. 2-chloro-5-nitrobenzene-1-sulfonyl chloride (105 mg, 411 µmol), 1.1 eq. TEA (63 µl, 450 µmol) in 2.0 ml DCM was stirred at r.t. for 17 h. Water and DCM were added, and the phases separated. The aqueous phase was extracted multiple times with DCM. The combined organic phases were evaporated to dryness. The remaining crude material was purified by preparative HPLC (acidic conditions) to give 95.0 mg (50% yield, 100% purity) of the title compound **8f**.

UPLC-MS (Waters Acquity, acidic conditions): ^tR = 1.45 min. MS (ESI+): *m/z* = 458.2 $[M+H]^+$.

^1H NMR (400 MHz, DMSO-d_6) δ ppm 11.06 (s, 1 H), 8.65 (d, *J* = 2.53 Hz, 1 H), 8.40 (dd, *J* = 8.62, 2.79 Hz, 1 H), 8.01 - 8.08 (m, 2 H), 7.97 (d, *J* = 8.87 Hz, 1 H), 7.69 (d, *J* = 8.62 Hz, 1 H), 7.49 (d, *J* = 2.28 Hz, 1 H), 7.43 (d, *J* = 8.36 Hz, 2 H), 7.18 (dd, *J* = 8.74, 2.15 Hz, 1 H), 2.69 (q, *J* = 7.60 Hz, 2 H), 1.21 (t, *J* = 7.60 Hz, 3 H).

4-(5,6-Dimethyl-1,3-benzoxazol-2-yl)aniline (**8g–1**)

Polyphoshoric acid (33 ml, 290 mmol) was heated 180 °C and 1.0 g 4-aminobenzoic acid (7.29 mmol) were added under vigorous stirring. The resulting mixture was stirred at 180 °C for 10 min and 1.0 g 2-amino-4,5-dimethylphenol (7.29 mmol) were added in portions. The resulting mixture was stirred at 180 °C for an additional 2 hours. The mixture was added to ice water. KOH was added and the pH-value adjust to pH: 10. The aqueous phase was extracted multiple times with DCM. The combined organic phases were evaporated to dryness to give 1.1 g crude material of the title compound which was used in the next step without further purification.
UPLC-MS (Waters Acquity, acidic conditions): ^{t}R. = 1.23 min. MS (ESI+): m/z = 239.4 [M+H]+.

2-chloro-N-[4-(5,6-dimethyl-1,3-benzoxazol-2-yl)phenyl]-5-nitrobenzamide (**8g**)

A mixture of 4-(5,6-dimethyl-1,3-benzoxazol-2-yl)aniline (750 mg, 3.15 mmol), 1.3 eq. 2-chloro-5-nitrobenzoic acid (825 mg, 4.09 mmol), 1.5 eq. HATU (1.80 g, 4.72 mmol) and 5.0 eq. TEA (2.2 ml, 16 mmol) in 14 ml DMF was stirred at r.t. until complete conversion. DCM and water were added, and the phases separated. The aqueous phase was extracted multiple times with DCM. The combined organic phases were evaporated to dryness and the remaining crude material purified by flash chromatography (silica gel, hexane/ ethyl acetate gradient) followed by preparative HPLC (acidic conditions) to give 190 mg (14% yield, 100% purity) of the title compound **8g**.

UPLC-MS (Waters Acquity, acidic conditions): ^{t}R = 1.43 min. MS (ESI+): *m/z* = 422.3 $[M+H]^+$

^{1}H NMR (400 MHz, DMSO-d_6) δ ppm 11.07 (s, 1 H), 8.55 (d, *J* = 2.79 Hz, 1 H), 8.37 (dd, *J* = 8.74, 2.66 Hz, 1 H), 8.16 - 8.22 (m, 2 H), 7.90 - 7.96 (m, 3 H), 7.57 (d, *J* = 4.56 Hz, 2 H), 2.36 (s, 3 H), 2.34 (s, 3 H).

2-Chloro-N-[3-(5-ethyl-1,3-benzoxazol-2-yl)phenyl]-5-nitrobenzamide (**8h**) UPLC-MS (Waters Acquity, acidic conditions): ^{t}R = 1.44 min. MS (ESI+): m/z = 422.3 [M+H]+, 100% purity.

^{1}H NMR (400 MHz, DMSO-d_6) δ ppm 11.02 (s, 1 H), 8.74 (t, *J* = 1.77 Hz, 1 H), 8.56 (d, *J* = 2.53 Hz, 1 H), 8.37 (dd, *J* = 8.87, 2.79 Hz, 1 H), 7.95 - 8.00 (m, 1 H), 7.93 (d, *J* = 8.87 Hz, 1 H), 7.80 - 7.86 (m, 1 H), 7.72 (d, *J* = 8.36 Hz, 1 H), 7.59 - 7.68 (m, 2 H), 7.30 (dd, *J* = 8.49, 1.65 Hz, 1 H), 2.71 - 2.79 (m, 2 H), 1.25 (t, *J* = 7.60 Hz, 3 H).

2-Chloro-N-[2-(4-ethylphenyl)-1,3-benzoxazol-5-yl]-5-(trifluoromethyl)benzamide (**9a**)

A mixture of 2-(4-ethylphenyl)-1,3-benzoxazol-5-amine (80.0 mg, 336 µmol), 1.3 eq. 2-chloro-5-(trifluoromethyl)benzoic acid (98.0 mg, 436 µmol), 0.05 eq. DMAP (2.05 mg, 16.8 µmol), 4.0 eq. DIPEA (230 µl, 1.3 mmol) and 2.0 eq. PyBrop (313 mg, 671 µmol) in 1.5 ml DMF was stirred at r.t. until complete conversion. The reaction mixture was evaporated to dryness and the remaining crude material purified by preparative HPLC (acidic conditions) to give 45.0 mg (30% yield, 100% purity) of the title compound **9a**.

UPLC-MS (Waters Acquity, acidic conditions): ^{t}R = 1.48 min. MS (ESI+): *m/z* = 445.0 $[M+H]^+$

^{1}H NMR (400 MHz, DMSO-d_6) δ ppm 10.77 - 10.88 (m, 1 H), 8.23 (d, *J* = 1.77 Hz, 1 H), 8.11 - 8.15 (m, 2 H), 8.09 (d, *J* = 2.28 Hz, 1 H), 7.89 - 7.94 (m, 1 H), 7.83 - 7.89 (m, 1 H), 7.78 (d, *J* = 8.62 Hz, 1 H), 7.65 (dd, *J* = 8.74, 2.15 Hz, 1 H), 7.47 (d, *J* = 8.62 Hz, 2 H), 2.72 (d, *J* = 7.60 Hz, 2 H), 1.24 (t, *J* = 7.60 Hz, 3 H).

tert.-Butyl 2-chloro-5-(methylcarbamoyl)benzoate (**9b–2**)

A mixture of 3-(ter.t.-butoxycarbonyl)-4-chlorobenzoic acid (1.23 g, 4.79 mmol), 2.0 eq. methylamine (4.8 ml, 2.0 M, 9.6 mmol), 2.5 eq. DIPEA (2.1 ml, 12 mmol) and 3.0 eq. HATU (5.47 g, 14.4 mmol) in 289 ml DMF was stirred at r.t. until complete conversion.
Water and DCM were added, and the phases separated. The aqueous phase was extracted multiple times with DCM. The combined organic phases were evaporated to dryness and the remaining crude material purified by flash chromatography (silice gel, hexane/ ethyl acetate gradient) to give 900 mg (70% yeld) of the title compound.

UPLC-MS (Waters Acquity, acidic conditions): ^{t}R = 1.11 min. MS (ESI+): m/z = 270.1 [M+H]+.

2-Chloro-5-(methylcarbamoyl)benzoic acid (**9b–1**)

A mixture of tert.-butyl 2-chloro-5-(methylcarbamoyl)benzoate (900 mg, 3.34 mmol) and 8.3 ml HCl-solution (4N in dioxane, 10 eq.) was stirred at r.t. until complete conversion. DCM and water were added, and the phases separated. The aqueous phase was extracted multiple times with DCM. The aqueous phase was evaporated to dryness to give 700 mg (98% yield) of the title compound.

UPLC-MS (Waters Acquity, acidic conditions): ^{t}R = 0.59 min. MS (ESI+): m/z = 214.0 [M+H]+.

4-Chloro-N^3-[2-(4-ethylphenyl)-1,3-benzoxazol-5-yl]-N^1-methylbenzene-1,3-dicarboxamide (**9b**)

A mixture of 2-chloro-5-(methylcarbamoyl)benzoic acid (300 mg, 1.40 mmol) and 20 eq. thionyl chloride (2.0 ml, 28 mmol) was stirred at 80 °C for 2.5 hours. The mixture was

evaporated to dryness and used immediately in the next step. A mixture of 2-(4-ethylphenyl)-1,3-benzoxazol-5-amine (156 mg, 657 µmol) and 1.05 eq. 2-chloro-5-(methylcarbamoyl)benzoyl chloride (160 mg, 689 µmol) and 3.0 eq. TEA (270 µl, 2.0 mmol) in 4.0 ml THF at r.t. for 12 hours and evaporated to dryness. The crude material was taken up in DCM, stirred at r.t., filtered and evaporated to dryness again to give 150 mg (52 % yield, 98% purity) of the title compound **9b**.

UPLC-MS (Agilent, acidic conditions): ^{t}R = 1.29 min. MS (ESI+): *m*/*z* = 434.1 $[M+H]^+$.

^{1}H NMR (400 MHz, DMSO-d_6) δ ppm 10.87 (s, 1 H), 8.82 (br d, *J* = 4.82 Hz, 1 H), 8.27 (d, *J* = 1.77 Hz, 1 H), 8.09 - 8.16 (m, 3 H), 7.99 (dd, *J* = 8.36, 2.28 Hz, 1 H), 7.75 - 7.80 (m, 1 H), 7.66 - 7.74 (m, 2 H), 7.47 (d, *J* = 8.36 Hz, 2 H), 2.79 (d, *J* = 4.56 Hz, 3 H), 2.72 (d, *J* = 7.60 Hz, 2 H), 1.23 (t, *J* = 7.60 Hz, 3 H).

2-Chloro-N-[2-(4-ethylphenyl)-1,3-benzoxazol-5-yl]pyridine-3-carboxamide (**9c**)

A mixture of 2-(4-ethylphenyl)-1,3-benzoxazol-5-amine (100 mg, 420 µmol), 1.0 eq. 2-chloropyridine-3-carboxylic acid (66.1 mg, 420 µmol), 5.0 eq. TEA (290 µl, 2.1 mmol) and 2.0 eq. HATU (319 mg, 839 µmol) in 6.0 ml THF was stirred at r.t. until complete conversion and evaporated to dryness. Water and DCM were added, and the phases separated.
The organic phase was evaporated to dryness and the remaining crude material purified by preparative HPLC (acidic conditions) to give 14 mg (9% yield, 100% purity) of the title compound **9c**.

UPLC-MS (Waters Acquity, acidic conditions): ^{t}R = 1.27 min. MS (ESI+): *m*/*z* = 378.0 $[M+H]^+$

^{1}H NMR (400 MHz, DMSO-d_6) δ ppm 10.78 - 10.91 (m, 1 H), 8.56 (dd, *J* = 4.82, 1.77 Hz, 1 H), 8.22 (d, *J* = 2.03 Hz, 1 H), 8.10 - 8.16 (m, 3 H), 7.78 (d, *J* = 8.87 Hz, 1 H), 7.65 (dd, *J* = 8.87, 2.03 Hz, 1 H), 7.59 (dd, *J* = 7.60, 4.82 Hz, 1 H), 7.47 (d, *J* = 8.62 Hz, 2 H), 2.68 - 2.76 (m, 2 H), 1.24 (t, *J* = 7.60 Hz, 3 H).

2-Chloro-5-cyano-N-[2-(4-ethylphenyl)-1,3-benzoxazol-5-yl]benzamide (**9d**)

A mixture of 22-(4-ethylphenyl)-1,3-benzoxazol-5-amine (100 mg, 420 µmol), 1.5 eq. 2-chloro-5-cyanobenzoic acid (114 mg, 629 µmol, 5.0 eq. DIPEA (370 µl, 2.1 mmol) and 1.5 eq. HATU (239 mg, 629 µmol) in 2.4 ml DMF was stirred at r.t. until complete conversion and evaporated to dryness. The remaining crude material purified by preparative HPLC (acidic conditions) to give 68 mg (38% yield, 97% purity) of the title compound **9d**.

UPLC-MS (Waters Acquity, acidic conditions): ^{t}R = 1.36 min. MS (ESI+): *m*/*z* = 402.0 $[M+H]^+$.

^{1}H NMR (400 MHz, METHANOL-d_4) δ ppm 8.21 (d, *J* = 1.52 Hz, 1 H), 8.13 - 8.18 (m, 2 H), 8.03 (d, *J* = 2.03 Hz, 1 H), 7.85 (dd, *J* = 8.36, 2.03 Hz, 1 H), 7.74 (d, *J* = 8.36 Hz, 1 H), 7.65 - 7.69 (m, 1 H), 7.59 - 7.64 (m, 1 H), 7.43 (d, *J* = 8.62 Hz, 2 H), 2.76 (q, *J* = 7.60 Hz, 2 H), 1.29 (t, *J* = 7.60 Hz, 3 H).

N-[2-(4-Ethylphenyl)-1,3-benzoxazol-5-yl]-3-nitrobenzamide (**9e**)

A mixture of 2-(4-ethylphenyl)-1,3-benzoxazol-5-amine (90.0 mg, 378 µmol), 1.3 eq. 3-nitrobenzoic acid (82.1 mg, 491 µmol), 2 eq. PyBrop (352 mg, 755 µmol) and 5.0 eq. DIPEA (330 µl, 1.9 mmol) in 3.1 ml DMF was stirred at r.t. until complete conversion. The mixture was extracted multiple times with DCM. The combined organic phases were washed with water and evaporated to dryness. The remaining crude material purified by preparative HPLC (acidic conditions) to give 20 mg (14% yield, 99% purity) of the title compound **9e**.

UPLC-MS (Waters Acquity, basic conditions): ^{t}R = 1.36 min. MS (ESI+): *m*/*z* = 388.0 $[M+H]^+$.

^{1}H NMR (400 MHz, DMSO-d_6) δ ppm 10.77 (s, 1 H), 8.83 (t, *J* = 2.03 Hz, 1 H), 8.42 - 8.49 (m, 2 H), 8.27 (d, *J* = 1.77 Hz, 1 H), 8.09 - 8.16 (m, 2 H), 7.87 (t, *J* = 7.98 Hz, 1 H), 7.72 - 7.82 (m, 2 H), 7.47 (d, *J* = 8.62 Hz, 2 H), 2.68 - 2.77 (m, 2 H), 1.24 (t, *J* = 7.60 Hz, 3 H).

2-Chloro-N-[2-(3-methylphenyl)-1,3-benzoxazol-5-yl]-3-nitrobenzamide (**9f**)

A mixture of 2-(3-methylphenyl)-1,3-benzoxazol-5-amine (99.0 mg, 441 µmol), 1.2 eq. 2-chloro-3-nitrobenzoic acid (107 mg, 530 µmol), 5.0 eq. TEA (310 µl, 2.2 mmol) and 1.2 eq. HATU (201 mg, 530 µmol) in 2.0 ml DMF was stirred at r.t. until complete conversion and evaporated to dryness. The remaining crude material purified by flash chromatography (silica gel, hexane/ ethyl acetate gradient) to give 83 mg (46% yield, 100% purity) of the title compound **9f**.

UPLC-MS (basic conditions): ^{t}R = 1.34 min. MS (ESI+): m/z = 408.2 [M+H]+.

^{1}H NMR (400 MHz, DMSO-d_6) δ ppm 10.91 (s, 1 H), 8.24 (d, *J* = 1.77 Hz, 1 H), 8.18 (dd, *J* = 8.11, 1.52 Hz, 1 H), 7.95 - 8.07 (m, 3 H), 7.72 - 7.83 (m, 2 H), 7.66 (dd, *J* = 8.74, 2.15 Hz, 1 H), 7.44 - 7.55 (m, 2 H), 2.44 (s, 3 H).

N-[2-(4-Ethylphenyl)-1,3-benzoxazol-5-yl]-5-nitrothiophene-2-carboxamide (**9g**)

A mixture of 2-(4-ethylphenyl)-1,3-benzoxazol-5-amine (90.0 mg, 378 µmol), 1.3 eq. 5-nitrothiophene-2-carboxylic acid (85.0 mg, 491 µmol), 2 eq. PyBrop (352 mg, 755 µmol) and 5.0 eq. DIPEA (330 µl, 1.9 mmol) in 3.1 ml DMF was stirred at r.t. until complete conversion. DCM and water were added, and the phases separated. The aqueous phase was extracted multiple times with DCM. The combined organic phases were evaporated to dryness. Methanol was added to the remaining crude material and the solid material filtered off to give 15 mg (10% yield, 100% purity) of the title compound **9g**.

UPLC-MS (Agilent, acidic conditions): ^{t}R = 1.42 min. MS (ESI+): *m/z* = 394.1 [M+H]$^{+}$.

^{1}H NMR (400 MHz, DMSO-d_6) δ ppm 10.81 (s, 1 H), 8.24 (d, *J* = 4.31 Hz, 1 H), 8.20 (d, *J* = 2.03 Hz, 1 H), 8.07 - 8.15 (m, 3 H), 7.80 (d, *J* = 8.87 Hz, 1 H), 7.69 (dd, *J* = 8.87, 2.03 Hz, 1 H), 7.47 (d, *J* = 8.36 Hz, 2 H), 2.72 (q, *J* = 7.60 Hz, 2 H), 1.24 (t, *J* = 7.60 Hz, 3 H.

N-[2-(4-Ethylphenyl)-1,3-benzoxazol-5-yl]prop-2-enamide (**9h**)

A mixture of 2-(4-ethylphenyl)-1,3-benzoxazol-5-amine (99.0 mg, 415 µmol), 1.3 eq. prop-2-enoic acid (38.9 mg, 540 µmol), 4.0 eq. DIPEA (290 µl, 1.7 mmol) and 1.2 eq. HATU (190 mg, 499 µmol) in 1.8 ml DMF was stirred at r.t. until complete conversion. DCM and water were added, and the phases separated. The aqueous phase was extracted multiple times with DCM. The combined organic phases were evaporated to dryness. The remaining crude material purified by preparative HPLC (acidic conditions) to give 26 mg (20% yield, 97% purity) of the title compound **9h**.

UPLC-MS (Waters Acquity, acidic conditions): ^{t}R = 1.21 min. MS (ESI+): *m/z* = 293.0 [M+H]$^{+}$

^{1}H NMR (400 MHz, DMSO-d_6) δ ppm 10.34 (s, 1 H), 8.23 (d, *J* = 2.03 Hz, 1 H), 8.09 - 8.14 (m, 2 H), 7.73 (d, *J* = 8.62 Hz, 1 H), 7.58 (dd, *J* = 8.87, 2.03 Hz, 1 H), 7.46 (d, *J* = 8.36 Hz, 2 H), 6.41 - 6.52 (m, 1 H), 6.26 - 6.34 (m, 1 H), 5.79 (dd, *J* = 9.89, 2.03 Hz, 1 H), 2.72 (q, *J* = 7.60 Hz, 2 H), 1.21 - 1.26 (m, 3 H).

N-[2-(4-Ethylphenyl)-1,3-benzoxazol-5-yl]-2-fluoro-5-nitrobenzamide (**9i**)

A mixture of 2-(4-ethylphenyl)-1,3-benzoxazol-5-amine (80.0 mg, 336 µmol), 1.3 eq. 2-fluoro-5-nitrobenzoic acid (80.8 mg, 436 µmol), 2 eq. PyBrop (313 mg, 671 µmol), 0.05 eq. DMAP (2.05 mg, 16.8 µmol) and 4.0 eq. DIPEA (230 µl, 1.3 mmol) in 1.5 ml DMF was stirred at r.t. until complete conversion. DCM and water were added, and the phases separated. The aqueous phase was extracted multiple times with DCM. The combined organic phases were evaporated to dryness. The remaining crude material was purified by preparative HPLC (acidic conditions) to give 30 mg (22% yield, 100% purity) of the title compound **9i**.

UPLC-MS (Waters Acquity, acidic conditions): ^{t}R = 1.37 min. MS (ESI+): *m/z* = 406.0 [M+H]$^{+}$

^{1}H NMR (400 MHz, DMSO-d_6) δ ppm 10.70 - 10.95 (m, 1 H), 8.59 (dd, *J* = 5.96, 2.91 Hz, 1 H), 8.45 - 8.52 (m, 1 H), 8.23 (d, *J* = 2.03 Hz, 1 H), 8.10 - 8.16 (m, 2 H), 7.79 (d, *J* = 8.62 Hz, 1 H), 7.63 - 7.75 (m, 2 H), 7.47 (d, *J* = 8.36 Hz, 2 H), 2.68 - 2.76 (m, 2 H), 1.24 (t, *J* = 7.60 Hz, 3 H).

2-Bromo-N-[2-(4-ethylphenyl)-1,3-benzoxazol-5-yl]-5-nitrobenzamide (**9j, BAY-4931**)

A mixture of 2-(4-ethylphenyl)-1,3-benzoxazol-5-amine (200 mg, 839 µmol), 1.05 eq. 2-bromo-5-nitrobenzoic acid (217 mg, 881 µmol), 2 eq. PyBrop (783 mg, 1.68 mmol), 0.05 eq. DMAP (5.13 mg, 42.0 µmol) and 4.0 eq. DIPEA (580 µl, 3.4 mmol) in 3.7 ml DMF was stirred at r.t. until complete conversion. DCM and water were added, and the phases separated. The aqueous phase was extracted multiple times with ethyl acetate. The combined organic phases were evaporated to dryness. The remaining crude material was purified by preparative HPLC (acidic conditions) to give 65 mg (16% yield, 100% purity) of the title compound **9j**.

^{1}H NMR (400 MHz, DMSO-d_6) δ ppm 10.88 (s, 1 H), 8.47 (d, J = 2.53 Hz, 1 H), 8.26 (dd, J = 8.87, 2.79 Hz, 1 H), 8.22 (d, J = 2.03 Hz, 1 H), 8.11 - 8.16 (m, 2 H), 8.07 (d, J = 8.87 Hz, 1 H), 7.79 (d, J = 8.87 Hz, 1 H), 7.65 (dd, J = 8.74, 2.15 Hz, 1 H), 7.47 (d, J = 8.62 Hz, 2 H), 2.72 (q, J = 7.60 Hz, 2 H), 1.24 (t, J=7.60 Hz, 3 H). LC-MS (acidic conditions): ^{t}R = 1.43 min. MS (ESI+): m/z = 468.2 [M+H]$^{+}$.

UPLC-MS (Waters Acquity, acidic conditions): ^{t}R = 1.44 min. MS (ESI+): *m/z* = 466.1 [M+H]$^{+}$. Refer to Figure S6 for HPLC trace **9j** (**BAY-0069**)

N-[2-(4-ethylphenyl)-1,3-benzoxazol-5-yl]-2-iodo-5-nitrobenzamide (**9k**)

A mixture of 2-(4-ethylphenyl)-1,3-benzoxazol-5-amine (70.0 mg, 294 µmol), 1.2 eq2-iodo-5-nitrobenzoic acid (103 mg, 353 µmol), 2.4 eq. DIPEA (120 µl, 710 µmol) and 1.2 eq. HATU (134 mg, 353 µmol) in 2.7 ml DMF was stirred at r.t. until complete conversion. The reaction mixture was evaporated to dryness and the remaining crude material purified by preparative HPLC (acidic conditions) to give 37 mg (24% yield, 100% purity) of the title compound **9k**.

UPLC-MS (Agilent, acidic conditions): ^{t}R = 1.42 min. MS (ESI+): *m/z* = 514.0 [M+H]$^{+}$.

^{1}H NMR (400 MHz, DMSO-d_6) δ ppm 10.81 (s, 1 H), 8.34 (d, *J* = 2.79 Hz, 1 H), 8.28 (d, *J* = 8.62 Hz, 1 H), 8.21 (d, *J* = 1.77 Hz, 1 H), 8.13 (d, *J* = 8.11 Hz, 2 H), 8.04 (dd, *J* = 8.62, 2.79 Hz, 1 H), 7.80 (d, *J* = 8.87 Hz, 1 H), 7.66 (dd, *J* = 8.87,

2.03 Hz, 1 H), 7.48 (d, J = 8.62 Hz, 2 H), 2.69 - 2.77 (m, 2 H), 1.24 (t, J = 7.60 Hz, 3 H).

Solubility from DMSO in buffer pH 6.5: Aqueous solubility was determined by an orientating high throughput screening method. Solubility was determined in PBS buffer pH 6.5 containing 1% DMSO. Test compounds were applied as 1 mM DMSO solution. After addition of PBS buffer pH 6.5 solutions were shaken for 24 h at room temperature. Undissolved material was removed by filtration using a MultiScreen Solubility Filter Plate (Millipore) according to the manufacturers' protocol. The compound dissolved in the filtrate was quantified by HPLC-UV. The response was fitted to a one-point standard curve prepared in DMSO.

Stability of compounds versus nucleophilic thiols: (Cysteine, glutathione) at 37°C was performed essentially as described[18]. Stability versus nucleophiles was determined by HPLC-UV. 5 µl of a 10 mM solution of compound in DMSO were solved in 1 ml acetonitrile. Reaction occurred in the presence of a 100-fold excess of thiol. Cysteine and Glutathione, respectively, were dissolved in buffer pH 7.4 to give a 500 µM reaction solution. 100 µl of drug solution were added to 1 ml thiol reaction solution. Injections were made immediately after mixing for time zero injection and then again after 1, 2, 4 and 24 h. Compounds were incubated at 37°C. Degradation rate (recovery in %) was calculated by relating peak areas after 1, 2, 4 and 24 h to the time zero injection.

Mass Spectrometry Covalent Binding Assay: Determination relative covalent binding [%] to PPARγ via denaturing intact mass analysis.

5 µM human recombinant PPARγ were incubated with 5 µM of compound (n=2) at room temperature. Each samples contained 1 % DMSO, incl. the PPARγ control without compound. Reactions was stopped after 1 h via the addition of 2 µl 4 % TFA to 10 µl reaction volume. Samples were measured on a Waters I Class UPLC coupled to a Waters SYNAPT G2-S quadrupole-TOF electrospray instrument operating in ESI+ mode. The Waters I Class UPLC was equipped with a Waters Mass Prep C4, 2.1 x 5mm. The column temperature was set to 65 °C and the flow rate was 100 µL /min. samples were measured with a gradient from 20% buffer B to 80% Buffer in 1.9 min with a total run time of 6 min. Buffer A contained water / 0.1 % formic acid and buffer B MeCN / 0.1 % formic acid. The MS setting was as followed: m/z range: 150 – 2200, Scan time: 1 s, Acquisition mode: Resolution, Acquisition time: 5 min.

PPARγ Biochemical Competitive Binding Assay: Assay performed as described previously[3]. Ultrahigh-throughput screen using LanthaScreen TR-FRET PPAR gamma Competitive Binding Assay. Essentially, epitope tagged PPARγ ligand binding domain (PPARγ-LBD) was tested in a biochemical TR-FRET interaction assay with the non-selective PPARγ ligand, Fluormone using LanthaScreen assay technology a (LanthaScreen TR-FRET PPAR gamma Competitive Binding Assay, ThermoFisher) performed according to the manufacturers protocol with adaptation for miniaturization to 1536-well format.

PPARγ:MED1 TR-FRET Assay: A biochemical interaction assay measuring the ligand-dependent changes in interactions between the PPARγ-LBD and a fluorescent peptide from the co-activator, *MED1* (TRAP220/ DRIP-2), was performed to evaluate ligand-dependent PPARγ : co-activator interactions This assay was performed according to the manufacturers protocol (LanthaScreen TR-FRET PPAR gamma Co-activator Assay, ThermoFisher). 2 hours after compound treatment in dose-response, plates were read using an EnVision Multimode Plate Reader (PerkinElmer) equipped with the LanthaScreen Advanced Dual Label filter set (PerkinElmer).

PPARγ:NCOR1 TR-FRET and PPARγ:NCOR2 CRR TR-FRET Assay: To evaluate inverse-agonist activity in a biochemical activity, we modified the PPARγ:MED1 TR-FRET assay to replace the TRAP220/DRIP-2 co-activator peptide included in the kit was with fluorescent labeled co-repressor peptides from NCOR1 (NCoR ID2), or NCOR2 (Smrt ID2) (ThermoFisher) as described previously[3]. 2 hours after compound treatment in dose-response with each point in duplicate, plates were read using a microplate reader in TR-FRET mode according to the manufacturers' instructions. EC_{50} and E_{max} values for PPARγ:NCOR2 are the mean from at least two experiments.

PPARA Biochemical Competitive Binding Assay: LanthaScreen™ TR-FRET PPAR alpha Competitive Binding Assay Kit (ThermoFisher) was performed according to the manufacturers protocol for evaluating binding to PPARA. 24 hours after compound treatment in dose response, plates were read using a microplate reader in TR-FRET mode according to the manufacturers' instructions.

GAL4-NHR-LBD chimera reporter gene assays: CHO cells expressing the Firefly Luciferase gene under control of an GAL4 promoter (pFR-Luc, Stratagene) were stably transfected with fusion proteins of the GAL4 DNA binding domain (pFC-dbd, Stratagene) and the indicated nuclear hormone receptor ligand binding domain (NHR-LBD). 2500 cells were plated per well in 384-well plates in OptiMEM with 2.5% fetal bovine serum and allowed to adhere prior to adding compounds in ten concentrations, each in quadruplicate. 6 hours after compound treatment, reporter activity was quantified by lysis and addition of the substrate Luciferin. Luminescence activity was detected using a luminescence plate reader. Every measurement was performed in at least 2 independent experiments. Receptors include human PPARγ, PPARA, PPARD, in addition to mouse versions (Pparγ).

Cell Lines: UM-UC-9 cells were purchased from European Collection of Authenticated Cell Cultures (EcACC). PaCaDD-188 and PaCaDD-161 were purchased from DSMZ (Germany). All other cell lines were obtained from the Cancer Cell Line Encyclopedia[19] (Broad Institute, Cambridge, MA), which obtained them from the original source and

performed cell line authentication. Cell lines were tested for mycoplasma contamination.

RT112-FABP4-NLucP reporter gene assay: The PPARγ reporter assay was performed as described[3]. Essentially, the NanoLuciferase gene was engineered into the 3'-UTR of the canonical PPARγ target gene, *FABP4.* in RT112/84 cells using Cas9-guided homology dependent repair. A single cell clone was selected and expanded. 5,000 to10,000 cells were plated per well in 384-well plates in culture media containing 10% fetal bovine serum and allowed to adhere prior to adding compounds in dose response. 20 hours after compound treatment, reporter activity was quantified using Nano-Glo Luciferase Assay System (Promega, Madison, Wisconsin) and luminescence activity was detected using a luminescence plate reader. IC_{50} and E_{max} values were calculated from 4 replicates per concentration and the mean of at least two independent experiments.

Colony Formation Assay and Crystal Violet Staining: Cells were plated in triplicate in 12-well plates in 1mL of media per well and allowed to adhere prior to treatment with vehicle or compound. All cell lines were grown in MEMα containing 10% FBS, with the exception of PaCaDD-188 and PaCaDD-161, which were grown in Dresden media as previously described[19]. Plating density was determined prior in order to achieve approximately 80% confluency in 7-14 days with vehicle treatment. For the experimental plates, cells were treated with DMSO vehicle, neutral antagonist GW9662 (100nM), T0070907 (100nM), or BAY 4931 (100nM) using the HP-D300e Digital Dispenser (Tecan). Every 3-4 days, media and compounds were replenished. When vehicle-treated cells reached approximately 80% confluency, cells were stained with crystal violet as[20] described. Briefly, media was removed and cells were washed with PBS. Cells were fixed using 4% formaldehyde in PBS for 30 min at room temperature. The formaldehyde solution was removed and the cells were stained with crystal violet for another 30 min, after which the stain was removed and washed out. The plates were allowed to dry inverted overnight and imaged the following day using an Epson Perfection 600 scanner.

UM-UC-9 Proliferation: UM-UC-9 were grown in MEM alpha media (Gibco) containing 10% heat-inactivated fetal bovine serum (Sigma). The cells were stably transduced with a lentiviral expression vector encoding the TagGFP-Histone-2B protein (pTagGFP2-H2B, Evrogen). Cells were plated at 500 cells per well in a 384-well view-plate and allowed to attach at 37 C for 2 hours. Plates were dosed with compounds at indicated concentration in duplicate. 7 days after addition of compound, nuclei were counted with the use of an IncuCyte S3 Live Cell Imager. Cell counts were normalized to vehicle control and data reported as IC50, Emax and percent of vehicle control. All compounds reported were also evaluated in an independent experiment read out at day 5 with similar results.

FABP4 RTqPCR: High-throughput RTqPCR was performed using LightCycler 1536 (Roche) instrument according to the manufacturers protocol. RT112/84 cells were incubated the compounds for 24 hours and *FABP4* (primer sequence: TAAACTGGTGGTGG AATGCG, GCGAACTTCAGTCCAGGTCA, TCATGAAA GGCGTCACTTCCACGAGA) expression was measured to monitor the activity of *PPARG*, *RPL30* (primer sequence: GTCCCGCTCCTAAGGCAG, GTTGATCGACT CCAGCGACT, AGATGGTGGCCGCAAAGAAGACGAA) was used as a housekeeper gene. The qPCR was performed as a one-step measurement in cell lysates using LightCycler RNA Virus Master PCR Kit (Roche) according to the manufacturers protocol in a total volume of 1 µl. The data were first normalized to the housekeeping gene and are shown as relative expression compared to vehicle control.

In Vitro Metabolic Stability assay in Rat Hepatocytes: Generation of primary Hepatocyte suspension: Hepatocytes from male Wistar rats were isolated via a two-step perfusion method. After perfusion, the liver was carefully removed from the rat, the liver capsule was opened and the hepatocytes were gently shaken out into a Petri dish with ice-cold Williams' medium E (WME). The resulting cell suspension was filtered through sterile gauze into 50 mL Falcon tubes and centrifuged at 50 × g for 3 min at room temperature. The cell pellet was resuspended in 30 mL of WME and centrifuged through a Percoll gradient two times at 100 × g. The hepatocytes were washed again with WME and resuspend-ed in medium containing 5% FCS. Cell viability was determined by trypan blue exclusion. For the metabolic stability assays, liver cells were distributed in WME containing 5% FCS to glass vials at a density of 1.0 × 106 vital cells/mL.

The test compound was added to a final concentration of 1 µM. Organic solvent in the incubations was limited to ≤0.01% DMSO and ≤1% acetonitrile. During incubation, the hepatocyte suspensions were continuously shaken at 580 rpm and aliquots were taken at 2, 8, 16, 30, 45, and 90 min to which an equal volume of cold acetonitrile was immediately added. Samples were frozen at –20 °C overnight, and subsequently centrifuged at 3000 rpm for 15 min. The supernatant was analyzed with an Agilent 1200 HPLC system with MS/MS detection. The half-life of a test compound was determined from the concentration–time plot. From the half-life, the intrinsic and the in vitro predicted blood clearances were calculated as well as the hepatic extraction ratio EH = (CLb/LBF)·100%, according to the 'well-stirred' liver model[22]. In combination with the standardized liver blood flow (LBF) of 4.2 L/h/kg, a specific liver weight of 32 g/kg body weight and amount of liver cells in vivo (1.1 × 108 cells/g liver) and in vitro (1.0 × 106/mL) the in vitro blood clearance (CLb, in vitro) and the maximal bioavailability (Fmax in % = 1-EH*100%) were calculated.

In Vitro Metabolic Stability in Liver Microsomes:
The in vitro metabolic stability of test compounds was determined by incubation at 1 µM in a suspension of liver microsomes in 100 mM phosphate buffer pH 7.4 (NaH2PO4·H2O + Na2HPO4·2H2O) and at a protein concentration of 0.5 mg/mL at 37 °C. The microsomes were activated by adding a cofactor mix containing 8 mM glucose-6-phosphate, 0.5 mM NADP, and 1 IU/mL glucose-6-phos¬phate dehydrogenase in phosphate buffer pH 7.4. The metabolic assay was started shortly afterwards by adding the test compound to the incubation at a final volume of 1 mL. During incubation, the microsomal suspensions were continuously shaken at 580

rpm and aliquots were taken at 2, 8, 16, 30, 45, and 60 min. Further handling and analysis as per the hepatocyte method described above with the human specific scaling factors of a liver blood flow of 1.32 L/h/kg and a specific liver weight of 21 g/kg body weight.

Estimation of Plasma Protein Binding by Flux Dialysis: Binding of test compounds to plasma proteins is measured by a modification of standard equilibrium dialysis in a 96-well format using HT-Dialysis equipment made of Teflon at 37°C and 5% CO2 atmosphere. The Flux dialysis method is based on the principle that the initial flux rate (R_slope) of a compound is proportional to the product of compound initial concentration, fu and unbound dialysis membrane permeability (P_mem). Therefore, fu can be determined from R_slope when membrane P_mem is known. Common equipment and assay specific P_mem of 75.2x10-6cm/s which was established was used for calculation[23].

In brief, a semipermeable membrane (regenerated cellulose, MWCO 12-14K) separates the plasma donor and plasma receiver side filled with 150 µl plasma each. The test compound is added to the donor side at 1 µM and binds to plasma proteins. The unbound fraction of the test compound passes the membrane and distributes on both sides until equilibrium is reached. The flux rate as the rate of compound appearance into the receiver side is approximated from the time course of the quotient of receiver and donor concentration (R) by non-linear regression including data from an entire time course. For this purpose, samples are taken at different time points (up to 96 h) from the donor and receiver side and the relative compound concentration (peak area ratios analyte/IS) is measured by LC-MSMS analytics. Prior to this both sides are matrix matched (diluted with buffer and plasma to achieve the same matrix of 10% plasma) and subsequently precipitated with a fourfold volume of methanol containing an appropriate internal standard (IS).

Caco-2 Permeability: Caco-2 cells (DSMZ) were seeded at a density of 2.5 × 105 cells/well on 24-well insert plates, 0.4 µm pore size, 0.3 cm2 (Costar) and grown for 13–15 d in DMEM medium supplemented with 10% FCS, 1% GlutaMAX (100 ×, Gibco), 100 U/mL penicillin, 100 µg/mL streptomycin (Gibco), and 1% non-essential amino acids (100 ×). Cells were maintained at 37 °C in a humidified 5% CO2 atmosphere. Medium was changed every 2–3 d.

The bidirectional transport assay for the evaluation of Caco-2 permeability was undertaken in 24-well insert plates using a robotic system (Tecan). Before the assay was run, the culture medium was replaced by transport medium (FCS-free HEPES carbonate transport buffer pH 7.2). For the assessment of monolayer integrity, the transepithelial electrical resistance (TEER) was measured. Only monolayers with a TEER of at least 400 Ω*cm2 were used. Test compounds were predissolved in DMSO and added either to the apical or basolateral compartment at a final concentration of 2 µM. Evaluation was done in triplicate. Before and after incubation for 2 h at 37 °C, samples were taken from both compartments and analyzed, after precipitation with MeOH, by LC-MS/MS. The apparent permeability coefficient (Papp) was calculated both for the apical to basolateral (A→B) and the basolateral to apical (B→A) direction using following equation: Papp = (Vr/P0)(1/S)(P2/t), where Vr is the volume of medium in the receiver chamber, P0 is the measured peak area of the test compound in the donor chamber at t = 0, S is the surface area of the monolayer, P2 is the measured peak area of the test compound in the acceptor chamber after incubation for 2 h, and t is the incubation time. The efflux ratio basolateral (B) to apical (A) was calculated by dividing Papp B–A by Papp A–B.

Metabolite Identification in rat Hepatocytes: The test compound was incubated at 37 °C in a hepatocyte suspension in a round-shaking water bath at 116 rpm containing $1x10^6$ cells/ mL for 1, 2 and 4 h. 5 µM test compound was added from a 0.1 mM stock solution dissolved in acetonitrile. Enzymatic activities of all hepatocyte preparations were measured using a variety of standard substrates. All hepatocytes exhibited good activities. The incubations were terminated by the addition of acetonitrile (approx. 30% (v/v)) and stored at 18 °C until analysis. Prior to analysis the samples were thawed and centrifuged at 12000 rpm for 10 min. Aliquots of 10 µL of the supernatant were used to control the recovery of radioactivity by liquid scintillation counting. Aliquots of the supernatants were transferred into HPLC vials and analyzed by HPLC and on-line MS detection using the Orbitrap Fusion Lumos mass spectrometer and parallel split to UV detection. Exact mass and mass changes in comparison with the parent drug in combination with the fragment pattern from MS/MS were used to propose the structures of metabolites or confirm structures with that of reference compounds.

CYP Inhibition: The inhibitory potency of test compounds towards CYP450-dependent metabolic pathways was determined in pooled human liver microsomes (Xenotech, USA) by applying individual CYP isoform selective standard probes (CYP1A2: phenacetin; CYP2C8: amodiaquine; CYP2C9: diclofenac; CYP2D6: dextromethorphan; CYP3A4: midazolam). Reference inhibitors were included as positive controls. Incubation conditions (protein and substrate concentration, incubation time) were optimized regarding linearity of metabolite formation. Assays were processed in 96-well microtiter plates at 37 °C using a Genesis Workstation (Tecan, Crailsheim, Germany). After protein precipitation, metabolite formation was quantified by LC-MS/MS analysis, followed by inhibition evaluation and IC_{50} calculation.

PXR NOEL Assay: A HepG2 cell line stably-cotransfected with a vector for human PXR and a Luciferase reporter gene under the control of a human CYP3A4 promotor were seeded in a 384 well plate and cultivated at 37 °C/5% CO2 in humidified air. 24h prior read-out the cells were treated with compound in a ten-step serial dilution of 1:3 starting at the highest test concentration of 50 µM and ending at 2 nM. Rifampicin was incubated in the same manner as positive control. In addition, for the normalization of the luminescence signal cells were incubated with Rifampicin at a concentration of 16.7 µM corresponding to 100% activation, as well as DMSO for background luminescence corresponding to 0% activation (n=32 wells each). Cells were lysed and incubated with the Luciferase substrate ONE-Glo™ Reagent (Promega, Madison WI, USA) according to manufacturer's instructions and luminescence signal was detected in a plate

reader. A concentration-dependent increase of the luciferase activity above 10% of Rifampicin control was classified as PXR transactivation.

***In vivo* exposure**: All animal experiments were conducted in accordance with the German Animal Welfare Law and were approved by local authorities. Female NMRI nu/nu mice (Janvier, France) were dosed once at 100 mg/kg orally (p.o., in PEG400/Ethanol 9:1) (n=3 per group), intraperitoneally (i.p., in Solutol/Ethanol/Water 4:1:5), or subcutaneously (s.c. in castoroil/benzylbenzoate 9:1). After 0.5h, 3h, 6h, 14h, 24h and 48h mice were sacrificed by decapitation and blood sampled in Potassium-EDTA tubes (Sarstedt, Germany). 100 µL plasma were used for analysis. Samples were precipitated by immediate administration of ice cold acetonitrile in 1:5 dilution. Samples were frozen at –20 °C overnight, and subsequently centrifuged at 3000 rpm for 15 min. The supernatant was analyzed with an Agilent 1200 HPLC system with MS/MS detection (AB Sciex, Framingham, MA, USA). Obtained exposures were corrected for plasma protein binding and put into relation to the antiproliferative $IC_{50}u$.

Structural Biology

Production of recombinant PPARγ: Codon optimized DNA sequences of human *PPARG* ligand binding domain (LBD), including residues 231-505 (Uniprot: P37231-1/Isoform 2), were synthesized (Gene Art, Life Technologies) and inserted into a modified pET22b vector for the overexpression of His-fusion proteins in Escherichia coli. All plasmids contained a thrombin protease cleavage site between the N-terminal 6xHis tag and the protein of interest. The protein was overexpressed in *Escherichia coli* BL21(DE3) overnight at 17°C after induction by IPTG.

In brief, cell pellets were resuspended in Lysis Buffer (20 mM TRIS pH 8.0, 150 mM NaCl, 1 mM DTT, and 20 mM imidazole with EDTA complete (Roche Applied Science)) and mechanically lysed using a Microfluidizer. Lysates were centrifuged at 30,000×g for 1 h, and clear supernatants were loaded on a 5 mL Protino Ni-NTA FPLC column (Macherey-Nagel GmbH). Bound protein was washed with Buffer A (20 mM TRIS pH 8.0, 150 mM NaCl, 1 mM DTT, and 20 mM imidazole) and high-salt Buffer A (300 mm NaCl). The His-/fusion-tag was removed by thrombin cleavage on the column overnight at 16°C. Cleaved PPARγ LDB and thrombin were eluted with Buffer A. The final step of purification included addition of Benzamidine Sepharose (Cytiva) to remove thrombin and size exclusion chromatography (10 mM TRIS pH 8.0, 100 mM NaCl, 5 mM DTT, and 1 mM EDTA). Purified protein was concentrated and stored at -80 °C.

Crystallization and structure determination: Purified human PPARγ LBD (9-12 mg/mL; frozen stock) was incubated with the peptide and ligand at the indicated protein-to-peptide-to-ligand molar ratios (Table S1) for 1-5 h at room temperature. Small-molecule ligands were dissolved to 100 mM in DMSO and further diluted to 10 mM in ethanol. The NCOR2-ID2 peptide (H2343-W2365: HASTNMGLEAIIRKALMGKYDQW) was purchased (Biosyntan GmbH, Germany) and dissolved without further purification to 10 mM in protein buffer. All crystallization experiments were performed at 20°C as sitting drops by adding equal volumes of sample and reservoir solution (100-300 nL). Crystallization conditions are listed in Supplementary Table 1. Crystals were frozen in liquid nitrogen after cryo-protection with 25% glycerol.

Diffraction data was collected at the Proxima-2 beamline (Soleil synchrotron, Paris, France) and was processed with XDS[24]. See Supplementary Table 1 for data and refinement statistics. The structures were solved using molecular replacement as implemented in Phaser and Dimple[25]. Further refinement of the initial models was accomplished through multiple rounds of refinement in Refmac5[25] and manual fitting and re-building in Coot[27]. Restraints for small-molecule ligands were generated with ProDrg[28] and modified for covalent linkage to a cysteine residue with JLigand[29].

The authors would like to acknowledge for their technical support and helpful discussions; Sven Christian, Clara Lemos, Ulrike Rauh (Sack), Simon Holton, Christian Lechner, Detlev Suelzle, Stefan Kaulfuss, Holger Summer, Ashley Eheim, Timothy Lewis, Steven Ferrara, Samantha Bender, Jennifer Roth, Céline Ronin and Fabrice Ciesielski (Novalix, Structural Biology Team).

ASSOCIATED CONTENT

Supporting Information

The Supporting Information is available free of charge on the ACS Publications website.

Supporting Information.pdf contains methods for RNA sequencing and PRISM multiplexed cell line panel. Supporting chart; Summary of HTS screen. Supporting figures; PRISM profiling, Colony formation assay, RNA sequencing analysis, X-ray structural comparison of binding mode of BAY-4931 and T0070907, pharmacodynamic analysis of tumors treated with compounds, and HPLC traces of *in vivo* compounds. Supporting tables; A table of Xray statistics can be found in Table S1 –Crystallographic data collection and refinement statistics.csv. Detailed PK parameters can be found in Table S2 – Pharmacokinetic Parameters.csv, and a table containing molecular string formula and a summary of the biochemical and cellular data can be found in Table S3 – Molecular String Formula.csv.

Authors will release the atomic coordinates and experimental data upon article publication. PDB ID codes for crystal structures: PPARγ, NCOR2, and **Compound 6a**, 8AQM; PPARγ, NCOR2, and **BAY-4931**, 8AQN.

RNA sequencing data is available from National Center for Biotechnology Gene Expresison Omnibus (NCBI GEO) under accession #: GSE210693.

AUTHOR INFORMATION

Corresponding Author

* Jonathan T. Goldstein, The Broad Institute of MIT and Harvard, 415 Main St, Cambridge, MA 02142. Email:

jgold@broadinstitute.org. https://orcid.org/0000-0003-0730-3891

Present Addresses

†Douglas L. Orsi, C4 Therapeutics, Watertown, MA

Maria Baco, Merck, Cambridge, MA

Samantha Bender, atai Life Sciences, Berlin, Germany

Craig A. Strathdee, Oncorus, Cambridge, MA

Lindsay Westlake, Summit Public Schools, Richmond, CA

Author Contributions

The manuscript was written through contributions of all authors. / All authors have given approval to the final version of the manuscript.

D. Orsi, C. Lemke, C. Strathdee, M. Baco, S Tang, A. Cherniack, L. Westlake, S. Bender, M. Kocak, M. Meyerson, and J. Goldstein declare receiving research support from Bayer.

Elisabeth Pook, Nico Bräuer, Anders Friberg, Philip Lienau, Timo Stellfeld, Ulf Brueggemeier, Vera Pütter, Hanna Meyer, and Knut Eis are current or former employees of Bayer AG.

Dr. Meyerson declares receiving research support from Bayer AG, Ono, and Janssen; having patent licensing royalties from Bayer and LabCorp; and serving as scientific advisory board member and consultant for Interline and Isabl.

Notes

Bayer AG, The Broad Institute of MIT and Harvard, and Dana-Farber Cancer Institute have filed a patent application for aspects of the work described in this paper.

ABBREVIATIONS

ADME, absorption, distribution, metabolism, and excretion; $AUC_{0\text{-tlast}}$, Area under the curve until the last measurable timepoint; CRR, co-repressor recruitment assay; CL_b, Clerance from blood; C_{max}, maximum plamsa concentration; E_{max}, maximal efficacy; GSH, glutathione; MeCN, acetonitrile; NCOR, nuclear receptor co-repressor; NHR-LBD, nuclear hormone receptor ligand-binding domain; RXR, retinoid X receptor.

REFERENCES

1. Rosen, E. D.; Sarraf, P.; Troy, A. E.; Bradwin, G.; Moore, K.; Milstone, D. S.; Spiegelman, B. M.; Mortensen, R. M., PPAR gamma is required for the differentiation of adipose tissue in vivo and in vitro. *Mol Cell* **1999,** *4* (4), 611-7.

2. Wright, M. B.; Bortolini, M.; Tadayyon, M.; Bopst, M., Minireview: Challenges and opportunities in development of PPAR agonists. *Mol Endocrinol* **2014,** *28* (11), 1756-68.

3. Goldstein, J. T.; Berger, A. C.; Shih, J.; Duke, F. F.; Furst, L.; Kwiatkowski, D. J.; Cherniack, A. D.; Meyerson, M.; Strathdee, C. A., Genomic Activation of PPARG Reveals a Candidate Therapeutic Axis in Bladder Cancer. *Cancer Res* **2017,** *77* (24), 6987-6998.

4. Korpal, M.; Puyang, X.; Jeremy Wu, Z.; Seiler, R.; Furman, C.; Oo, H. Z.; Seiler, M.; Irwin, S.; Subramanian, V.; Julie Joshi, J.; Wang, C. K.; Rimkunas, V.; Tortora, D.; Yang, H.; Kumar, N.; Kuznetsov, G.; Matijevic, M.; Chow, J.; Kumar, P.; Zou, J.; Feala, J.; Corson, L.; Henry, R.; Selvaraj, A.; Davis, A.; Bloudoff, K.; Douglas, J.; Kiss, B.; Roberts, M.; Fazli, L.; Black, P. C.; Fekkes, P.; Smith, P. G.; Warmuth, M.; Yu, L.; Hao, M.-H.; Larsen, N.; Daugaard, M.; Zhu, P., Evasion of immunosurveillance by genomic alterations of PPARγ/RXRα in bladder cancer. *Nature Communications* **2017,** *8* (1).

5. Halstead, A. M.; Kapadia, C. D.; Bolzenius, J.; Chu, C. E.; Schriefer, A.; Wartman, L. D.; Bowman, G. R.; Arora, V. K., Bladder-cancer-associated mutations in RXRA activate peroxisome proliferator-activated receptors to drive urothelial proliferation. *Elife* **2017,** *6*.

6. Tsherniak, A.; Vazquez, F.; Montgomery, P. G.; Weir, B. A.; Kryukov, G.; Cowley, G. S.; Gill, S.; Harrington, W. F.; Pantel, S.; Krill-Burger, J. M.; Meyers, R. M.; Ali, L.; Goodale, A.; Lee, Y.; Jiang, G.; Hsiao, J.; Gerath, W. F. J.; Howell, S.; Merkel, E.; Ghandi, M.; Garraway, L. A.; Root, D. E.; Golub, T. R.; Boehm, J. S.; Hahn, W. C., Defining a Cancer Dependency Map. *Cell* **2017,** *170* (3), 564-576 e16.

7. Lubet, R. A.; Fischer, S. M.; Steele, V. E.; Juliana, M. M.; Desmond, R.; Grubbs, C. J.,

Rosiglitazone, a PPAR gamma agonist: potent promoter of hydroxybutyl(butyl)nitrosamine-induced urinary bladder cancers. *Int J Cancer* **2008,** *123* (10), 2254-9.
8. Tannehill-Gregg, S. H.; Sanderson, T. P.; Minnema, D.; Voelker, R.; Ulland, B.; Cohen, S. M.; Arnold, L. L.; Schilling, B. E.; Waites, C. R.; Dominick, M. A., Rodent carcinogenicity profile of the antidiabetic dual PPAR alpha and gamma agonist muraglitazar. *Toxicol Sci* **2007,** *98* (1), 258-70.
9. Tuccori, M.; Filion, K. B.; Yin, H.; Yu, O. H.; Platt, R. W.; Azoulay, L., Pioglitazone use and risk of bladder cancer: population based cohort study. *BMJ* **2016**, i1541.
10. Marciano, D. P.; Kuruvilla, D. S.; Boregowda, S. V.; Asteian, A.; Hughes, T. S.; Garcia-Ordonez, R.; Corzo, C. A.; Khan, T. M.; Novick, S. J.; Park, H.; Kojetin, D. J.; Phinney, D. G.; Bruning, J. B.; Kamenecka, T. M.; Griffin, P. R., Pharmacological repression of PPARgamma promotes osteogenesis. *Nat Commun* **2015,** *6*, 7443.
11. Lee, G.; Elwood, F.; McNally, J.; Weiszmann, J.; Lindstrom, M.; Amaral, K.; Nakamura, M.; Miao, S.; Cao, P.; Learned, R. M.; Chen, J. L.; Li, Y., T0070907, a selective ligand for peroxisome proliferator-activated receptor gamma, functions as an antagonist of biochemical and cellular activities. *J Biol Chem* **2002,** *277* (22), 19649-57.
12. Brown, K. K.; Henke, B. R.; Blanchard, S. G.; Cobb, J. E.; Mook, R.; Kaldor, I.; Kliewer, S. A.; Lehmann, J. M.; Lenhard, J. M.; Harrington, W. W.; Novak, P. J.; Faison, W.; Binz, J. G.; Hashim, M. A.; Oliver, W. O.; Brown, H. R.; Parks, D. J.; Plunket, K. D.; Tong, W. Q.; Menius, J. A.; Adkison, K.; Noble, S. A.; Willson, T. M., A novel N-aryl tyrosine activator of peroxisome proliferator-activated receptor-gamma reverses the diabetic phenotype of the Zucker diabetic fatty rat. *Diabetes* **1999,** *48* (7), 1415-24.
13. Webb, P.; Anderson, C. M.; Valentine, C.; Nguyen, P.; Marimuthu, A.; West, B. L.; Baxter, J. D.; Kushner, P. J., The nuclear receptor co-repressor (N-CoR) contains three isoleucine motifs (I/LXXII) that serve as receptor interaction domains (IDs). *Mol Endocrinol* **2000,** *14* (12), 1976-85.
14. Shang, J.; Mosure, S. A.; Zheng, J.; Brust, R.; Bass, J.; Nichols, A.; Solt, L. A.; Griffin, P. R.; Kojetin, D. J., A molecular switch regulating transcriptional repression and activation of PPARgamma. *Nat Commun* **2020,** *11* (1), 956.
15. Krinke, G., *The laboratory rat*. Academic Press: San Diego, Calif., 2000; p xiv, 756 p.
16. Davies, B.; Morris, T., Physiological parameters in laboratory animals and humans. *Pharm Res* **1993,** *10* (7), 1093-5.
17. Yu, C.; Mannan, A. M.; Yvone, G. M.; Ross, K. N.; Zhang, Y. L.; Marton, M. A.; Taylor, B. R.; Crenshaw, A.; Gould, J. Z.; Tamayo, P.; Weir, B. A.; Tsherniak, A.; Wong, B.; Garraway, L. A.; Shamji, A. F.; Palmer, M. A.; Foley, M. A.; Winckler, W.; Schreiber, S. L.; Kung, A. L.; Golub, T. R., High-throughput identification of genotype-specific cancer vulnerabilities in mixtures of barcoded tumor cell lines. *Nat Biotechnol* **2016,** *34* (4), 419-23.
18. Cee, V. J.; Volak, L. P.; Chen, Y.; Bartberger, M. D.; Tegley, C.; Arvedson, T.; McCarter, J.; Tasker, A. S.; Fotsch, C., Systematic Study of the Glutathione (GSH) Reactivity of N-Arylacrylamides: 1. Effects of Aryl Substitution. *J Med Chem* **2015,** *58* (23), 9171-8.
19. Ruckert, F.; Aust, D.; Bohme, I.; Werner, K.; Brandt, A.; Diamandis, E. P.; Krautz, C.; Hering, S.; Saeger, H. D.; Grutzmann, R.; Pilarsky, C., Five primary human pancreatic adenocarcinoma cell lines established by the outgrowth method. *J Surg Res* **2012,** *172* (1), 29-39.
20. Crowley, L. C.; Christensen, M. E.; Waterhouse, N. J., Measuring Survival of Adherent Cells with the Colony-Forming Assay. *Cold Spring Harb Protoc* **2016,** *2016* (8).
21. Pang, K. S.; Rowland, M., Hepatic clearance of drugs. I. Theoretical considerations of a "well-stirred" model and a "parallel tube" model. Influence of hepatic blood flow, plasma and blood cell binding, and the hepatocellular enzymatic activity on hepatic drug clearance. *J Pharmacokinet Biopharm* **1977,** *5* (6), 625-53.
22. Kalvass, J. C.; Phipps, C.; Jenkins, G. J.; Stuart, P.; Zhang, X.; Heinle, L.; Nijsen, M.; Fischer, V., Mathematical and Experimental Validation of Flux Dialysis Method: An Improved Approach to Measure Unbound

Fraction for Compounds with High Protein Binding and Other Challenging Properties. *Drug Metab Dispos* **2018,** *46* (4), 458-469.
23. Kabsch, W., Xds. *Acta Crystallogr D Biol Crystallogr* **2010,** *66* (Pt 2), 125-32.
24. Wojdyr, M.; Keegan, R.; Winter, G.; Ashton, A., DIMPLE - a pipeline for the rapid generation of difference maps from protein crystals with putatively bound ligands. *Acta Crystallogr A* **2013,** *69*, S299-S299.
25. Murshudov, G. N.; Skubak, P.; Lebedev, A. A.; Pannu, N. S.; Steiner, R. A.; Nicholls, R. A.; Winn, M. D.; Long, F.; Vagin, A. A., REFMAC5 for the refinement of macromolecular crystal structures. *Acta Crystallogr D* **2011,** *67*, 355-367.
26. Emsley, P.; Lohkamp, B.; Scott, W. G.; Cowtan, K., Features and development of Coot. *Acta Crystallographica Section D-Biological Crystallography* **2010,** *66*, 486-501.
27. Schuttelkopf, A. W.; van Aalten, D. M. F., PRODRG: a tool for high-throughput crystallography of protein-ligand complexes. *Acta Crystallogr D* **2004,** *60*, 1355-1363.
28. Lebedev, A. A.; Young, P.; Isupov, M. N.; Moroz, O. V.; Vagin, A. A.; Murshudov, G. N., JLigand: a graphical tool for the CCP4 template-restraint library. *Acta Crystallogr D Biol Crystallogr* **2012,** *68* (Pt 4), 431-40.

TABLE OF CONTENTS GRAPHIC

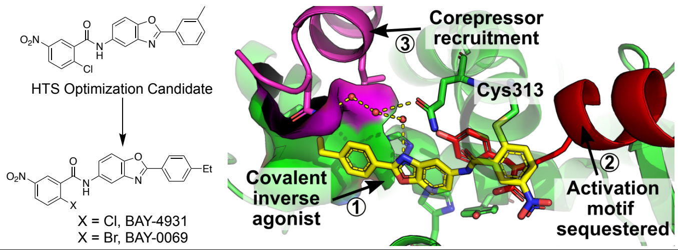

Supplement: Supplementary file 1 — jm2c01379_si_001.pdf [file jm2c01379_si_001.pdf]
