# Supplementary material for: Correction to “Discovery and Structure-Based Design of Potent Covalent PPARγ Inverse-Agonists BAY-4931 and BAY-0069”
Source: J Med Chem. 2022 Dec 21;66(1):1082. doi: 10.1021/acs.jmedchem.2c02002 (PMC9841977; doi:10.1021/acs.jmedchem.2c02002)
Supplement: Supplementary file 1 — jm2c02002_si_001.pdf [file jm2c02002_si_001.pdf]

Supporting Information for

Discovery and Structure-Based Design of Potent Covalent PPARγ Inverse-Agonists BAY-4931 and BAY-0069

Douglas L. Orsi,[1] Elisabeth Pook,[2] Nico Bräuer,[3] Anders Friberg,[3] Philip Lienau,[2] Christopher T. Lemke,[1] Timo Stellfeld,[3] Ulf Brüggemeier,[2] Vera Pütter,[3] Hanna Meyer,[3] Maria Baco,[4] Stephanie Tang,[4] Andrew D. Cherniack,[4,5] Lindsay Westlake,[4] Samantha A. Bender,[4] Mustafa Kocak,[4] Craig A. Strathdee[4], Matthew Meyerson,[4,5,6,7] Knut Eis,[2] and Jonathan T. Goldstein[4,*]

AUTHOR ADDRESS [1]Center for the Development of Therapeutics, Broad Institute of MIT and Harvard, Cambridge, MA, 02142, USA. [2]Research and Development, Pharmaceuticals, Bayer AG, 13353, Berlin, Germany. [3]Nuvisan ICB GmbH, 13353, Berlin, Germany [4]Cancer Program, Broad Institute of MIT and Harvard, Cambridge, MA, 02142, USA. [5]Department of Medical Oncology, Dana-Farber Cancer Institute, Boston, MA, 02215, USA. [6]Center for Cancer Genomics, Dana-Farber Cancer Institute, Boston, MA, 02215, USA. [7]Department of Genetics and Medicine, Harvard Medical School, Boston, MA, 02115, USA.

Corresponding Author

* Jonathan T. Goldstein, The Broad Institute of MIT and Harvard, 415 Main St, Cambridge, MA 02142. Email: jgold@broadinstitute.org

Contents of Supporting Information

Supplementary Methods S2

Chart S1: Summary of HTS screen S4

Figure S1: Cell line panel profiling over 12 days of treatment using PRISM multiplexed cancer cell line platform...S5

Figure S2: Colony formation assay. S6

Figure S3: RNA sequencing analysis of HT-1197 cells treated with PPARG modulators. S7

Figure S4. Comparison of the binding mode of BAY-4931 bound to PPARG/NCOR2 (colored as in Fig 4C) and T0070907 bound to PPARG alone (gray; 6c1i.pdb). S8

Figure S5: Relative expression of *CD36 and PLIN2* from UM-UC-9 xenograft tumors harvested at indicated timepoints after mice treated with a single dose of compound......................................S9

Figure S6: HPLC traces of *in vivo* and key compounds..S10

Figure S7: NOESY profile of compound 8c S11

Figure S8: NOESY profile of compound 8b S12

Supplementary Methods

RNA sequencing

HT-1197 cells were plated in 6-well plates in MEM alpha (Gibco) containing 10% FBS (Sigma). Compounds were added when cells reached approximately 70% confluency, and 24 hours later, cell were harvested for RNA isolation. Treatment includes vehicle (DMSO), rosiglitazone 500nM, SR10221 500nM, T0070907 500nM, BAY-4931 200nM. Samples run in triplicate.

RNA was isolated using RNEasy (Qiagen) according to the manufacturers' suggested protocol.

RNA library preparations and sequencing reactions were conducted at Azenta Life Sciences (South Plainfield, NJ, USA) as follows:

Extracted RNA samples were quantified using Qubit 2.0 Fluorometer (Life Technologies, Carlsbad, CA, USA) and RNA integrity was checked using Agilent TapeStation 4200 (Agilent Technologies, Palo Alto, CA, USA).

The RNA sequencing libraries were prepared using the NEBNext Ultra II RNA Library Prep Kit for Illumina using manufacturer's instructions (New England Biolabs, Ipswich, MA, USA). Briefly, mRNAs were initially enriched with Oligod(T) beads. Enriched mRNAs were fragmented for 15 minutes at 94 °C. First strand and second strand cDNA were subsequently synthesized. cDNA fragments were end repaired and adenylated at 3'ends, and universal adapters were ligated to cDNA fragments, followed by index addition and library enrichment by PCR with limited cycles. The sequencing libraries were validated on the Agilent TapeStation (Agilent Technologies, Palo Alto, CA, USA), and quantified by using Qubit 2.0 Fluorometer (ThermoFisher Scientific, Waltham, MA, USA) as well as by quantitative PCR (KAPA Biosystems, Wilmington, MA, USA). The sequencing libraries were clustered on one flowcell lanes. After clustering, the flowcell was loaded on the Illumina HiSeq instrument (4000 or equivalent) according to manufacturer's instructions. The samples were sequenced using a 2x150bp Paired End (PE) configuration. Image analysis and base calling were conducted by the HiSeq Control Software (HCS). Raw sequence data (.bcl files) generated from Illumina HiSeq was converted into fastq files and de-multiplexed using Illumina's bcl2fastq 2.17 software. One mismatch was allowed for index sequence identification.

Sequence data was analyzed using Desseq pipeline to map transcripts and calculate transcripts per million.

PRISM Multiplexed Cell Line Panel Screening:

Cell Lines

The current PRISM cell set consists of 940 cell lines representing more than 45 lineages including only adherent cell lines. These cell lines largely overlap with and reflect the diversity of the Cancer Cell Line Encyclopedia (CCLE) cell lines (see https://portals.broadinstitute.org/ccle). Cell lines were grown in RPMI 10% FBS without phenol red. Parental cell lines were stably infected with a unique 24-nucleotide DNA barcode via lentiviral transduction and blasticidin selection. After selection, barcoded cell lines were expanded and quality control evaluated by mycoplasma contamination test, a SNP test for confirming cell line identity, and barcode ID confirmation. Passing barcoded lines were then pooled] together and frozen in assay-ready vials.

PRISM Screening

Cell line pools (n=20, containing 22-25 cell lines per pool) were thawed, counted, and mixed together at equal abundance (489 total cell lines). 4e4 cells per well were plated into 6-well plates and cells were allowed to adhere overnight. The following day, one 6-well plate was lysed in DNA lysis buffer (20mM Tris-HCl (pH 8.4), 50mM KCl, 0.45% NP40, 0.45% Tween-20, 10% Proteinase K) at 60°C for 1 hour for baseline cell barcode measurement. Compounds (2 doses each, in triplicate) were added to remaining plates for 12-day treatment and subsequent lysis. PPARG inverse-agonists were treated at the following doses; T0070907 2.5µM, T0070907 250nM, BAY-4931 1000nM, and BAY-4931 100nM. DMSO was used as negative control and bortezomib (20µM) and fulvestrant (100nM) as positive controls. Every 4 days, cells were trypsinized and counted and then re-seeded/re-dosed for a total of 12 days of culture under continuous drug treatment. At each timepoint, 1e6 cells were lysed for controls. Crude lysates were frozen at -80ºC before being used for PCR amplification.

Barcode Amplification and Sequencing

Samples lysed in DNA Lysis Buffer are denatured at 95ºC and amplified with a OneTaq polymerase master mix. Custom primers from IDT allow samples to be dual-indexed for multiplexed Illumina sequencing by directly adding Illumina adapter sequences to the amplicon. Resulting products are quality control checked using gel electrophoresis and then pooled and purified for sequencing using the Xymo Select-a-Size DNA Clean & Concentrator kit. After pooling, the PCR product is quantified using the Qubit 3 Fluorometer. Samples are sequenced using Illumina NovaSeq S2 technology. On the NovaSeq platforms, samples are loaded onto the flow cell at a final concentration of 10pM with a 20% PhiX spike-in due to low diversity. Sequencing is run for 100 cycles, paired-read.

Screened compounds in primary HTS (PPARγ binding TR-FRET):	4,289,975
Primary HTS hits:<br>(hit limit < 49% of max signal; calculated IC_{50}: 4 μM)	**29,107**
Structure filter score:	- 10,291
Frequent hitters in cell-based HTS campaigns:<br>(active in at least 8 cell-based HTS assays)	- 2,679
Frequent hitters in biochemical HTS campaigns:<br>(active in at least 5 biochemical HTS assays; no changes in background signal)	- 2,135
Tested in hit confirmation:	15,152
Confirmed PPARγ binders: (IC_{50} < 10 μM in PPARγ binding assay)	11,421

Chart S1: Summary of HTS screen. Summary of results from the HTS screening campaign for PPARγ binders. An HTS screen of 4,289,975 compounds tested in single dose at 10μM was performed using PPARγ Biochemical Competitive Binding Assay with a signal threshold of >50% loss in signal. 29,107 primary screening hits were identified in the HTS screen. Primary hits were next filtered using proprietary software to remove candidates with undesirable chemical structure properties. Additionally, compounds that are frequent positives hits from historical cell-based and/or biochemical HTS screening assays were also removed, with some overlap in compounds between these filter sets. After filtering, a total of 15,152 primary screening hits remained and were subjected to testing in hit confirmation using PPARγ Biochemical Competitive Binding Assay with compounds in dose-response format. Of these, 11,142 were confirmed as PPARγ binders with IC_{50} in the biochemical competitive binding assay less than 10μM. Subsequently, candidates were evaluated in functional biochemical and cellular assays to evaluate mechanistic effects (eg. agonist, antagonist, inverse-agonist).

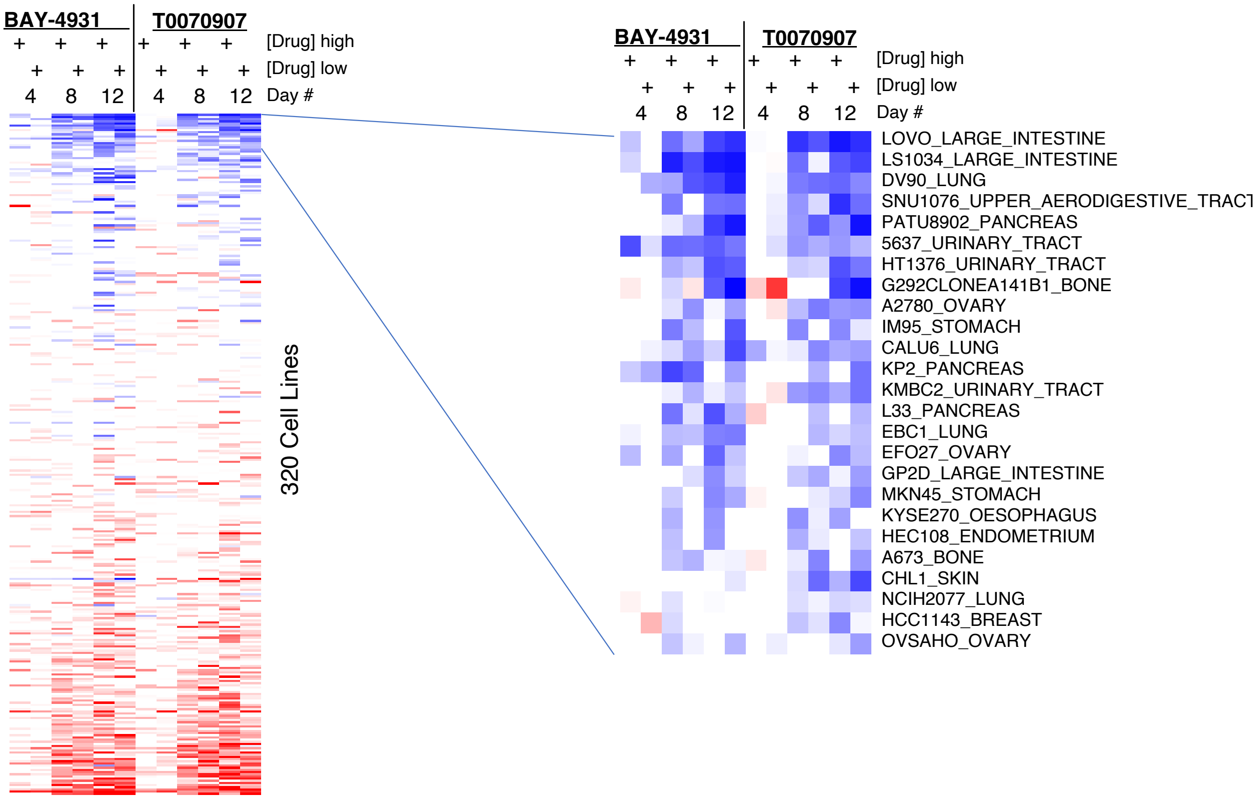

Figure S1: Cell line panel profiling over 12 days of treatment using PRISM multiplexed cancer cell line platform. Heatmap of growth rate inhibition score across entire panel of 320 cell lines which passed quality control (Left). Columns represent mean of three replicates and columns are separated by harvest day (Day #), and concentration of compounds; T0070907 [Drug] high (2.5µM), T0070907 [Drug] low (250nM), BAY-4931[Drug] high (1000nM), and BAY-4931 [Drug] low (100nM). Close-up of top 25 cell lines (right) sorted by average growth rate inhibition of all replicates combined for the day 8 and day 12 time points.

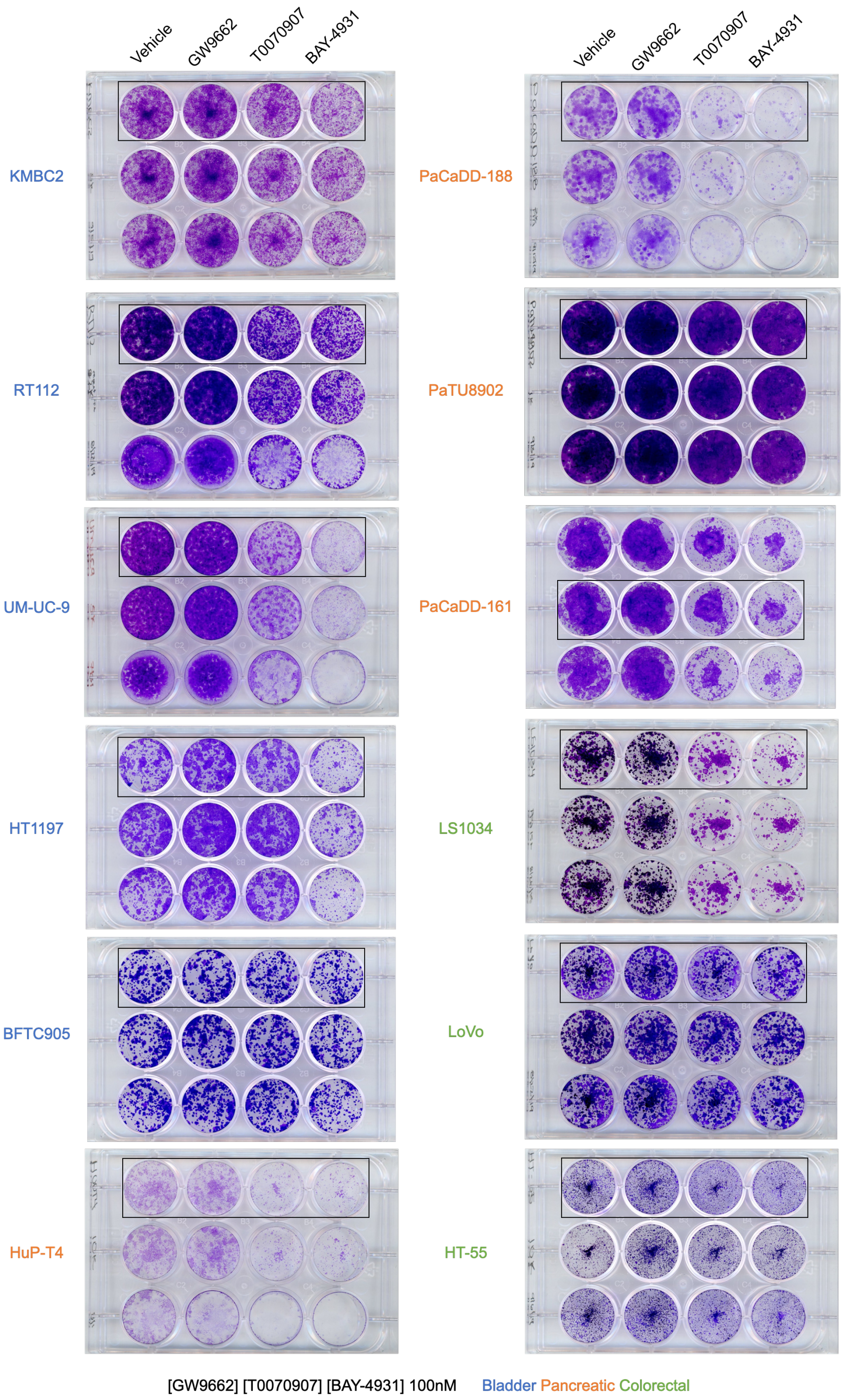

Figure S2: Colony formation assay. Full plate scans represented in Figure 6 by boxed row. Colony formation assays comparing effects of DMSO vehicle, GW9662 (100nM), T0070907 (100nM), or BAY-4931(100nM) in panel of bladder (blue), pancreatic (orange), and colorectal (green) cell lines after 7-14 days of dosing with crystal violet staining. (Addendum to figure 6).

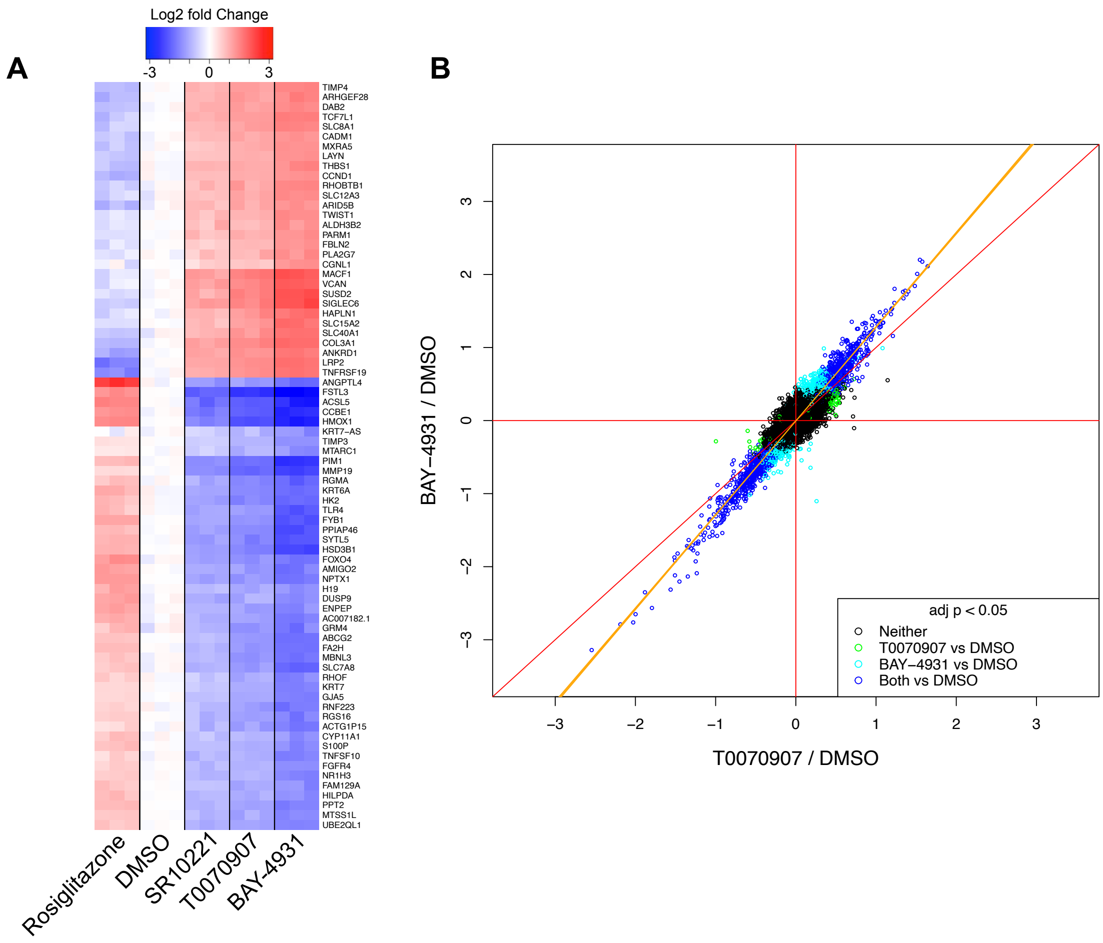

Figure S3: RNA sequencing analysis of HT-1197 cells treated with PPARG modulators. A. Heatmap of the Log2 fold Change in gene expression from RNAseq analysis of HT-1197 cells treated for 24 hours with PPARG modulators relative to DMSO control. Samples include; DMSO, Rosiglitazone (500nM), SR10221 (500nM), T0070907 (500nM), and BAY-4931 (200nM) (n=3) cutoff is 2.5 fold difference between vehicle and BAY-4931 with Adj p <0.01 based on Voom Limma. B. Comparison of gene expression Log2-fold change for T0070907 / DMSO versus BAY-4931 / DMSO. Sequence files are available from National Center for Biotechnology Gene Expression Omnibus (NCBI GEO) under accession #: GSE210693

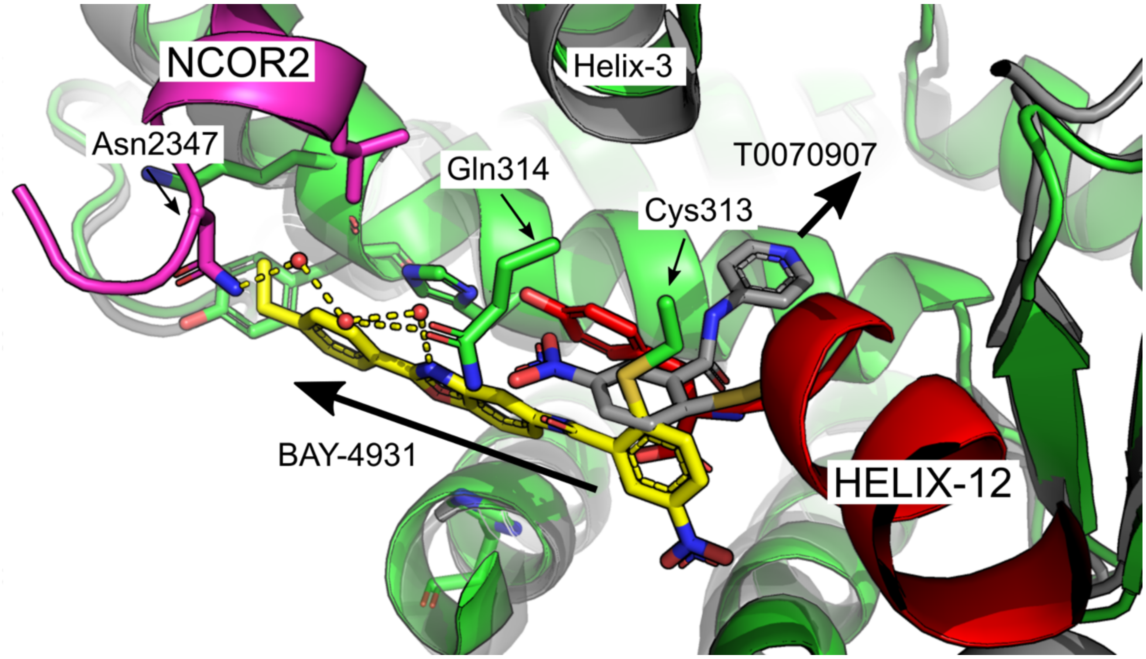

Figure S4. Comparison of the binding mode of BAY-4931 bound to PPARG/NCOR2 (colored as in Fig 4C) and T0070907 bound to PPARG alone (gray; 6c1i.pdb). T0070907 extends in a different direction, away from the NCOR2 binding site, thereby also prohibiting sequestering of Helix-12 (red) into the canonical ligand binding site. Please note, for clarity Helix-3 is not completely shown and only selected side chains are shown and labeled. Also, Helix-12 of the PPARG/T0070907 co-complex is not shown.

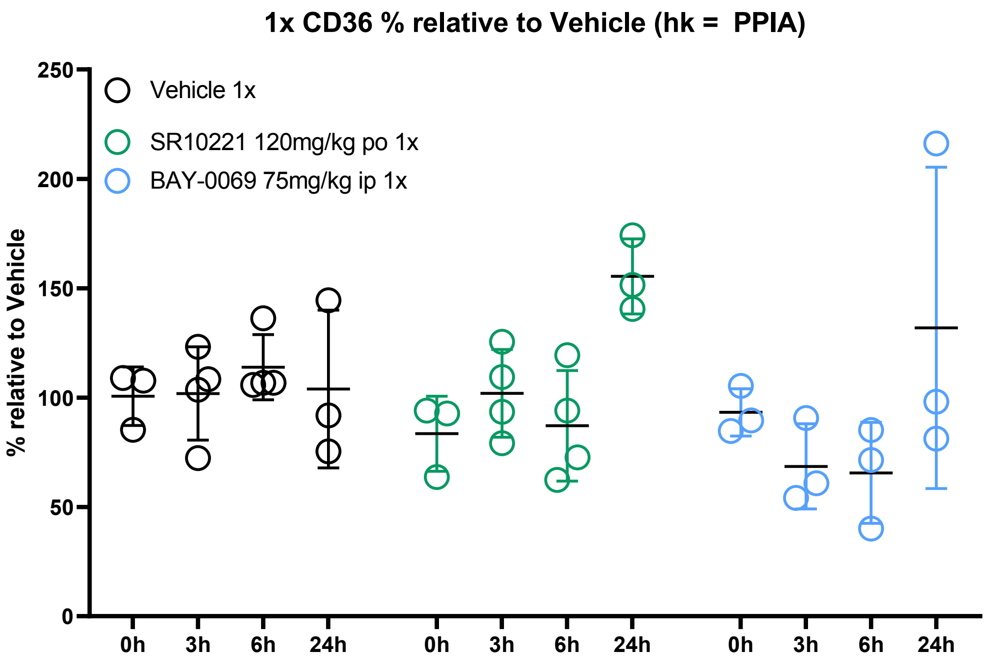

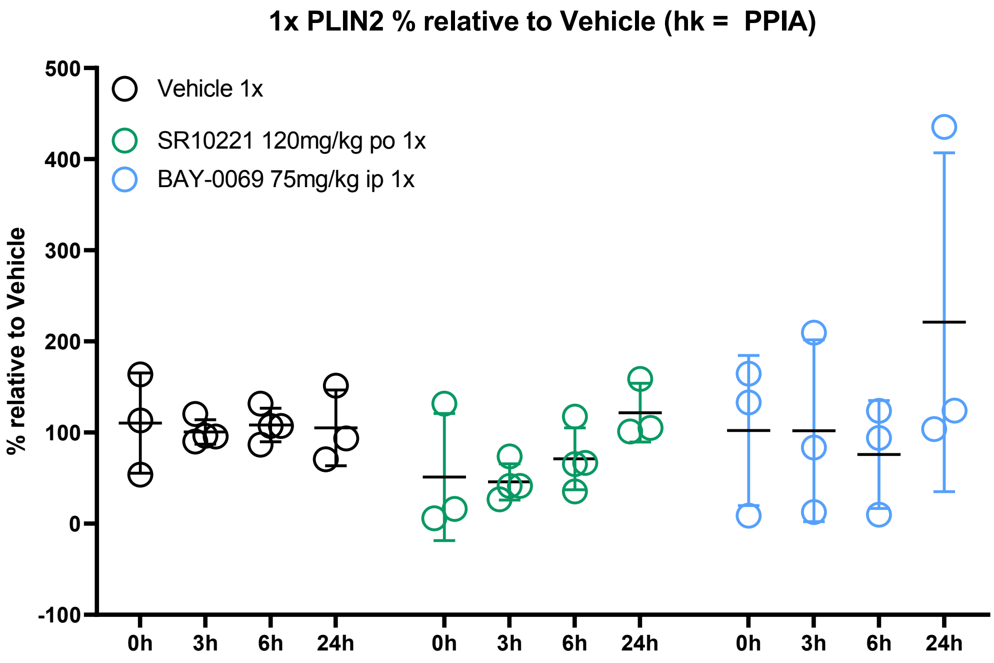

Figure S5: Relative expression of *CD36 and PLIN2* from UM-UC-9 xenograft tumors harvested at indicated timepoints after mice treated with a single dose of compound.

A. BAY-4931

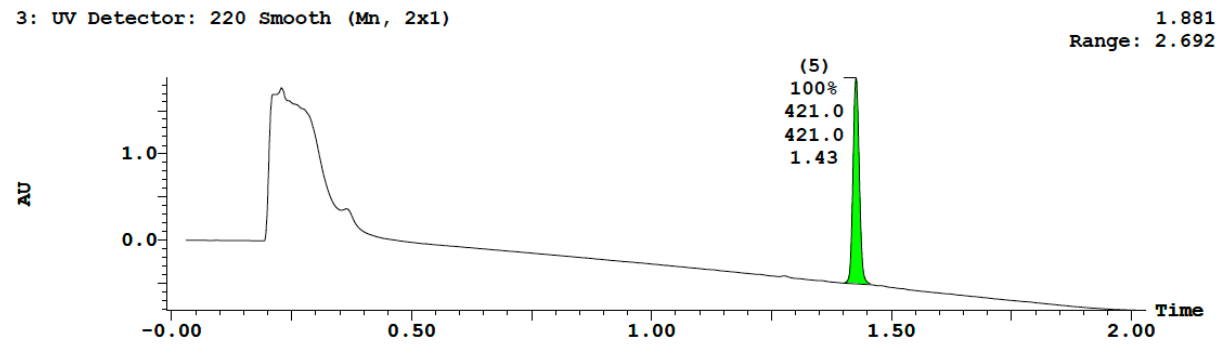

B. BAY-0069

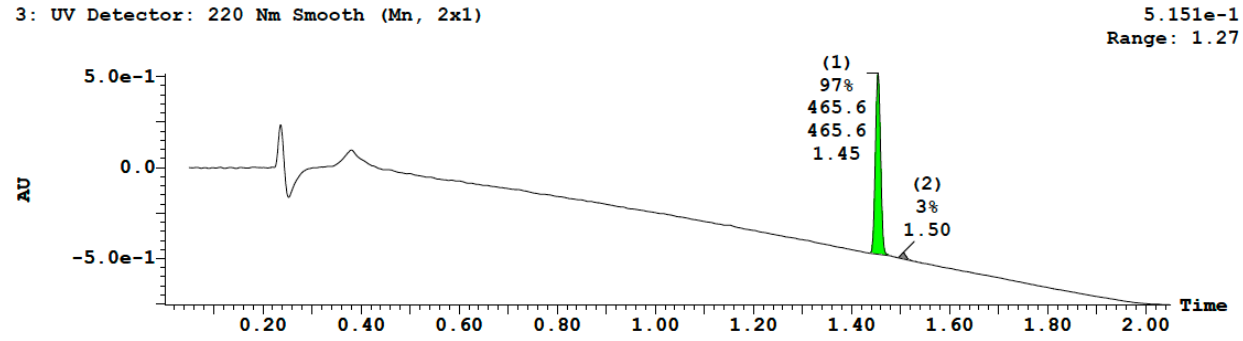

C. SR10221

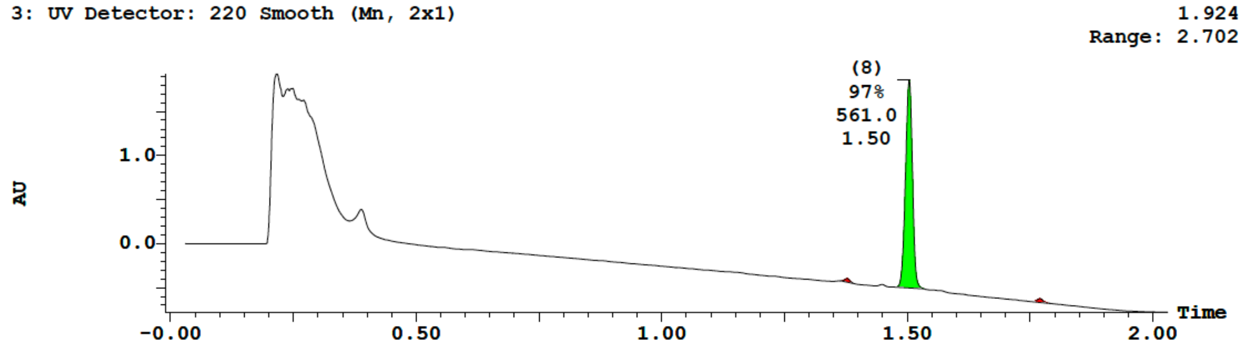

Figure S6: HPLC traces of *in vivo* and key compounds. HPLC traces with detection at UV 220nm for A) BAY-4931, B) BAY-0069, and C) SR10221.

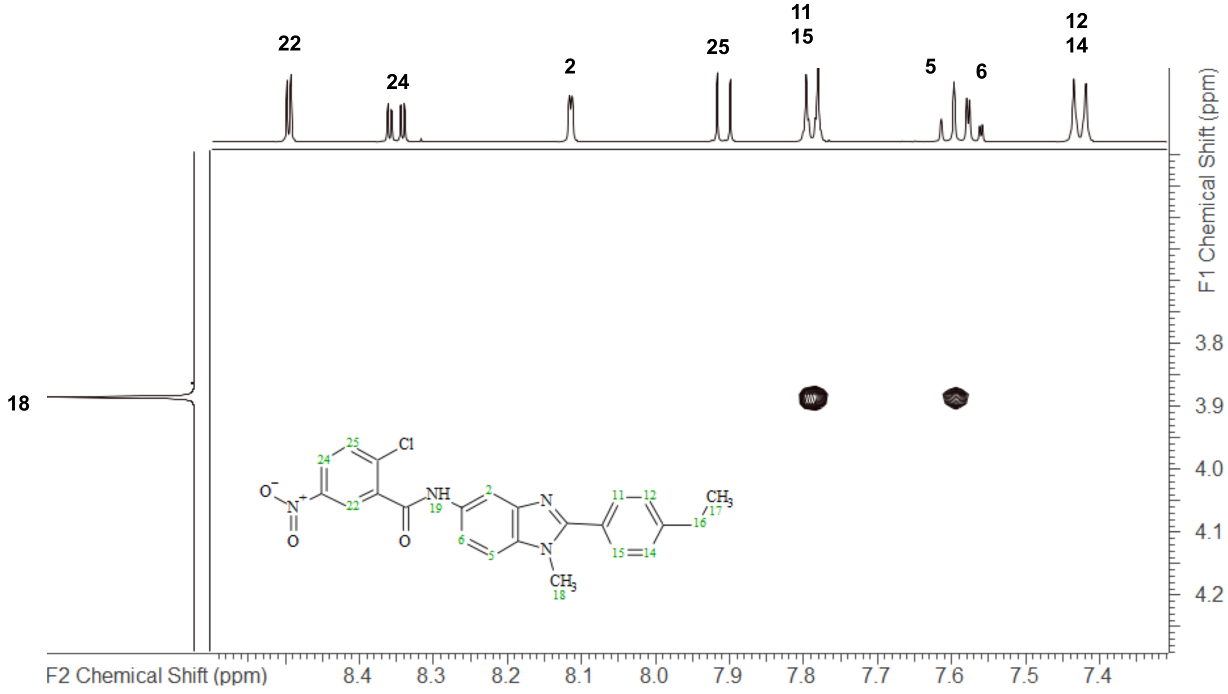

Figure S7: NOESY profile of compound 8c

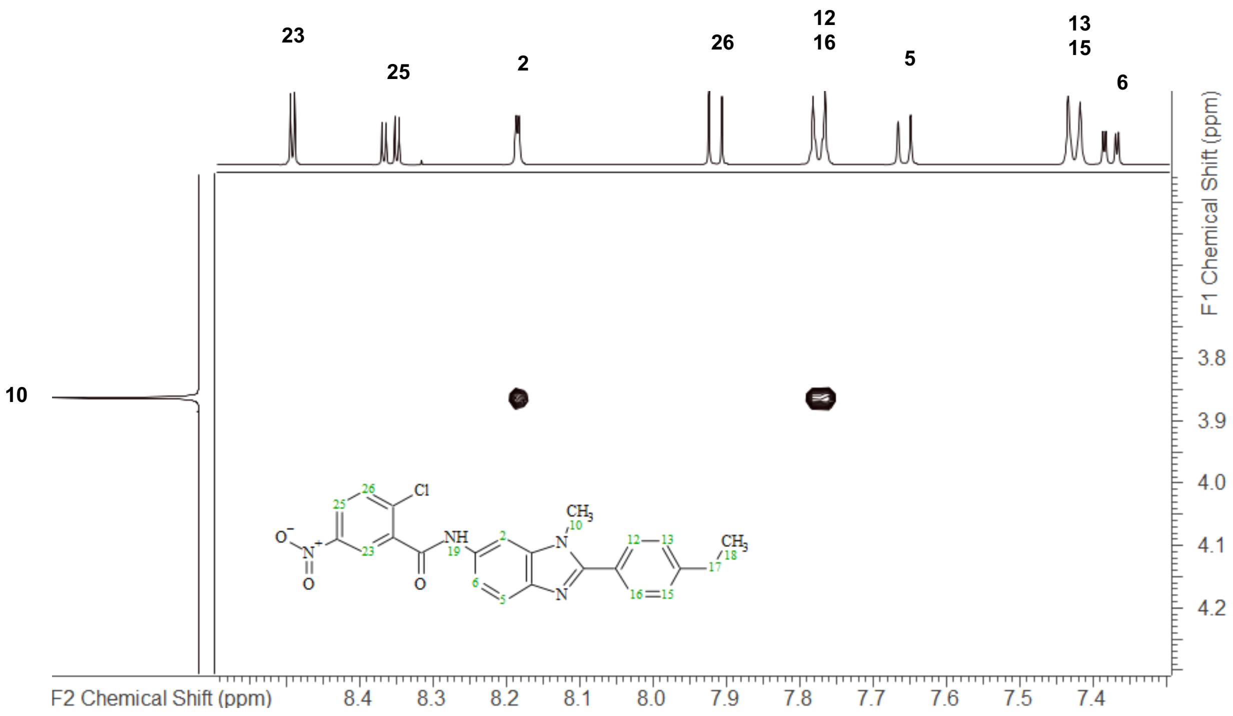

Figure S8: NOESY profile of compound 8b
